# Using Hearing Aids for Music: A UK Survey of Challenges and Strategies

**DOI:** 10.1177/23312165251396517

**Published:** 2026-01-22

**Authors:** Alinka E. Greasley, Amy V. Beeston, Robert J. Fulford, Harriet Crook, Jackie M. Salter, Robin Hake, Brian C. J. Moore

**Affiliations:** 1 School of Music, 171134 University of Leeds, Leeds, UK; 2 Department of Audiological Science, 7318 Sheffield Teaching Hospitals NHS Foundation Trust, Sheffield, UK; 3 School of Education, 228051 University of Leeds, Leeds, UK; 4 Department of Medical Physics and Acoustics, 597451 University of Oldenburg, Oldenburg, Germany; 5 Department of Psychology, 98528 University of Cambridge, Cambridge, UK

**Keywords:** music listening, hearing aids, hearing loss, audiology, technology, live music

## Abstract

Hearing aids, which are primarily designed to improve the intelligibility of speech, can negatively affect the perception and enjoyment of music. This large-scale survey study, conducted between 2016 and 2018, explored hearing aid use and preference behavior in both recorded and live music listening settings, aiming to understand the challenges and strategies used by listeners to improve their experiences, and how these may be affected by level of hearing loss (HL). One thousand five hundred and seven hearing aid users (mean age = 60 years) completed an online survey about their music listening behavior and use of hearing aids. Results showed that whilst hearing aids support engagement in music listening, they also present many issues and overall helpfulness is mixed. The most commonly reported issue was distortion and poor sound quality, particularly in loud or live contexts. The most frequently reported strategy for reducing distortion was to remove hearing aids altogether. Only a third of the sample reported using a music program and effectiveness was mixed, suggesting that manufacturer music programs do not currently provide significant benefits for music listening, and further research into the use, uptake and efficacy of music programs is needed. We call for further research into signal processing strategies for music especially for high sound levels such as live music or concert settings. The positive impact of mindsets supporting proactive behaviors, perseverance, adaptation, and experimentation with different technologies, genres, and listening environments was highlighted, strengthening the evidence base for audiologists to provide music listening guidance in the clinic.

## Introduction

The global prevalence of hearing loss (HL) is increasing. Approximately 1.5 billion people (∼20%) worldwide are currently living with HL and this is set to rise to 2.5 billion by 2050 (World Health Organisation, 2021). The main cause of this is age-related HL. However, an increasing number experience noise-induced HL (NIHL) as a result of exposure to loud sounds/noise, including music (Jiang et al., 2016). HL has many negative consequences, such as difficulties communicating with others, withdrawal from social activities, and loneliness/depression (Arlinger, 2003; Davis et al., 2016). HL also affects musical engagement, perception, and appreciation (Moore, 2016), leading to a reduction in musical activities and its associated health and well-being benefits, and negatively affecting quality of life (Greasley et al., 2020). Thirty-nine percent of musicians are estimated to have some form of NIHL, which is higher than for the general population (Akeroyd & Munro, 2024).

Hearing aids are the primary technology used to treat HL, and the global market is projected to grow from $11.3 billion in 2022 to $21.1 billion by 2030 (EHIMA, 2022), largely due to ageing populations. Hearing aids facilitate verbal communication and improve quality of life (Cox et al., 2014; Ferguson et al., 2019) and the growing market is driving technological advancements including advanced speech processing and wireless technologies (Chung & Zeng, 2024; Kollmeier & Kiessling, 2016). Despite this, hearing aid penetration rates (adoption) globally remain low. In the UK, around 2 million people use hearing aids but 7 million would benefit from them (RNID, 2024). Adoption varies widely by region and country due to variations in access to healthcare, availability of audiologists and affordability (Eurotrak 2025; WHO, 2021). Recent Eurotrak data suggest adoption rates of between 21% and 55% for European countries for those living with HL (Eurotrak, 2025). Another factor is persistent social stigma about wearing hearing aids (David & Werner, 2016; Ruusuvuori et al., 2021), although there is some evidence that this is reducing (Sindi et al., 2023). At a personal level, factors affecting adoption include limited effectiveness in noisy situations, sound quality, and issues with comfort and fit (Franks & Timmer, 2023; McCormack & Fortnum, 2013; Mothemela et al., 2023). A recent meta-analysis confirmed that severity of HL is a strong predictor of hearing aid uptake, alongside self-reported disability, and demographic factors such as access to financial support (Knoetze et al., 2023). Finally, it has been shown that music sound quality can affect the use of hearing aids by musicians, such that some hearing aids are preferred over others, and some hearing aids are not used at all during music listening or performing (Fulford et al., 2015; Greasley et al., 2020).

A likely reason for the limited adoption of hearing aids for music listening is that they are primarily designed for the perception of speech, not music. Music is ubiquitous, so amplification of music by hearing aids is likely to occur both in active participation in music listening and through passive exposure via media (e.g., TV and film) and other everyday environments. However, music typically has far wider dynamic and frequency ranges than speech, and this can result in distortion due to the limited dynamic range of the analog-to-digital converters used in hearing aids and due to the signal processing that is tailored for speech amplification (Chasin & Hockley, 2014; Moore, 2022). The acoustical properties of speech remain relatively homogenous across speakers (Lee et al., 2019), while music is heterogeneous, varying widely by genre (Lesimple et al., 2024). Manufacturers do offer solutions for music listening, typically in the form of music programs. However, evidence for their efficacy is mixed. Experimental work in tightly controlled conditions has shown that music programs are not necessarily preferred to speech programs when listening to music stimuli (Sandgren & Alexander, 2023; Vaisberg et al., 2017). Sandgren and Alexander (2023) found that sound quality ratings were higher for the music versus universal programs for only three of the seven brands tested, and that the manufacturer brand explained more of the variance in sound quality ratings than the programs themselves. One survey study found evidence that music programs made it easier to distinguish between different instruments, but did not improve clarity or tone quality (Madsen & Moore, 2014). Another survey study showed that only a quarter of hearing aid users had access to a music program and, of those who did, two-thirds reported worse sound quality using the music program than using their everyday program (Looi et al., 2019). In a qualitative study with musicians, Vaisberg et al. (2019) found that while a music program improved music balance and brightness for some, others reported no discernible benefit. Together, these findings indicate that music programs are currently neither wholly effective nor widely adopted, in spite of their prominent marketing by manufacturers.

Several studies have explored the experiences and behaviors of hearing aid users in music listening situations. Madsen and Moore's (2014) survey of 523 respondents represents an initial benchmark in the field. Most respondents reported that their hearing aids were helpful in both reproduced (TV/radio/stereo) and live music contexts (76% and 62% respectively), and that individual notes and melodies were “fairly easy” to hear. However, 53% reported distortion at least sometimes, 36% reported experiencing acoustic feedback, and around 25% found it difficult to hear both soft and loud passages, with a further 25% reporting that hearing aids over-amplified loud passages. Folk/country and solo instruments were reported to be easier to listen to than orchestral or rock music. The researchers also examined patterns in reported HL (mild, moderate, severe, and profound). Those with mild HL were more likely to find hearing aids helpful across the full range of musical contexts and genres, and reported less distortion and better audibility of notes and melodies. Those with severe HL were more likely to have difficulty hearing out individual notes and melodic lines and to experience music as too bright or shrill.

Further research has broadly confirmed these findings. Marchand (2019) found that whilst hearing aid users were engaged in music listening, they reported difficulties with understanding lyrics, lack of clarity, and poor tone quality, including sounds being too sharp/shrill or too loud. Interviews with musicians with HL revealed that sentiment around hearing aids is both positive and negative, and may be mediated by hearing aid type (digital or analogue, although the latter are rare nowadays), genres, instrument being played, musical training, location or proximity to the sound source, and history of HL (Fulford et al., 2011, 2015; Swann et al., 2023; Vaisberg et al. 2019). Fulford et al. (2015) found that while some musicians depended on hearing aids for musical participation, satisfaction overall was low, with many reporting distortion and poor sound quality. Some participants preferred older analogue hearing aids over digital, and a few reported removing their hearing aids altogether when performing (Fulford et al., 2015), a behavior also reported by 41% of listeners in the Marchand (2019) survey. Vaisberg et al. (2019) found that while hearing aids helped musicians perceive verbal instructions from the conductor (a key reason for wearing hearing aids in music performance settings), they also complained of poor sound quality and ineffectiveness of music programs. Another interview study with eight musicians (Swann et al., 2023) found that hearing aids made music sound brighter and helped with the perception of high frequencies, but some reported issues with poor tonal balance, dynamics and distortion.

It has been shown that increasing HL is associated with poorer music appreciation (Chern et al., 2022; Looi et al., 2019). Looi et al. (2019) surveyed 111 adult hearing aid users with post-lingual HL and found that those with severe loss were more likely to report reduced enjoyment as a result of their HL and that hearing aids negatively affected the sound of melodic music. However, all participants reported listening to music as much as they had before they experienced HL. Greasley et al. (2020) surveyed 176 patients attending an audiology clinic and found that 67% experienced problems with music listening at least sometimes, and 36% reported losing enjoyment of music. The most commonly reported issues were with pitch perception, volume/dynamics, lyrics, distortion, poor sound quality, and difficulties in live contexts. 13% had stopped listening to music or going to concerts, and many reported associated negative effects, including frustration, depression and feelings of exclusion. It can be inferred from these findings that the majority of hearing aid users continue to listen to music in spite of the reported difficulties. Recently, Chern et al. (2022) surveyed 109 hearing aid users and found that those with more severe hearing losses reported lower music enjoyment. However, this association was no longer significant when using hearing aids, suggesting that there is an overall benefit of using hearing aids for music listening, especially for those with more severe HL.

The reports of both benefits and problems in the use of hearing aids for music listening likely reflect the importance and ubiquity of music listening, with the result that people continue to engage in spite of difficulties experienced. Beyond highlighting problems experienced, more research is needed to understand what drives better adoption and success of hearing aid use in musical contexts. A recent large-scale consumer research study about hearing aid adoption and usage did not include questions about music engagement, but found that “improving music perception” was an emerging theme (Desai et al., 2024). If adoption of music programs remains low, yet a majority continue to use their hearing aids, what strategies do hearing aid users employ to improve their music listening experiences? Qualitative studies highlighting strategies adopted by professional or amateur musicians with HL may not generalize to nonmusicians. Little is known about the strategies and experiences of non-musicians when listening to music. The present research, carried out between 2016 and 2018 as part of the Hearing Aids for Music (HAfM) project, aimed to conduct a deeper analysis of hearing aid use and preference behaviors across different listening settings using a large sample of hearing aid users. Going beyond prior work, the study aimed to compare musical outcomes in recorded versus live settings and also sought to capture novel insights into strategies that are used to improve music listening experiences. It also aimed to provide an evidence-base for creating guidance to support audiologists and patients in conversations in the clinic about hearing aid use for music listening.

## Method

### Survey

An online survey was designed to capture key aspects of music listening behavior, hearing level, and hearing aid usage. The online survey consisted of 70 questions in eight sections (see Table 1 for main variables and Supplementary Materials, SM1 for full questionnaire). All survey questions were interpreted into British sign language (BSL) by a profoundly deaf BSL interpreter, with videos embedded in the survey (see Napier et al., 2018). This ensured accessibility for those for whom sign language would be the most useful mode of communication. Participants were given the opportunity to provide responses in a signed video if they wished.

**Table 1.** Main Survey Variables.

Demographics	Age; Gender; Highest academic qualification; Geographical location
Musical engagement and training	Engagement with music listening (e.g., importance, frequency, self-chosen); Avoidance of music listening; Musical training (e.g., level of education, instrumental proficiency); Working in a musical field; Whether hearing loss affects work in musical field
Musical preferences and uses of music	Preference for 18 genres (e.g., classical (orchestral), rock, pop); Importance of 8 musical features (e.g., lyrics, voice, instrumentation); Functions of music listening (e.g., pleasure, reminiscence, concentration)
Hearing	Unilateral or bilateral; Hearing level (Hs) speech descriptors; Hearing level (Hm) memory of audiologist category; Hearing level (Hd) audiometric data (if submitted); Age of onset (e.g., congenital, sudden change, presbycusis)
Hearing Aids	One/two hearing aids; Duration of wearing hearing aids; Duration wearing current hearing aids; Type of hearing aid; Dome/mold Make/model; Fitting organization; Volume control; Music program; Music program frequency of use
Recorded music contexts (e.g., at home, in the car, personal listening device)	Listening with/out hearing aids; Amount of music listening; Mode of listening; Assistive Listening Devices; Helpfulness of hearing aids for hearing musical elements; Difficulties experienced; Strategies for improving experiences; Ease of listening to musical genres; Overall helpfulness of hearing aids
Live music contexts	Listening with/out hearing aids; Number of live events attended per year; Type of events attended (e.g., acoustic/amplified, performance); Listen with/out hearing aids by type of event attended; Helpfulness of hearing aids for hearing musical elements; Difficulties experienced; Strategies for improving experiences; Ease of listening to musical genres; Overall helpfulness of hearing aids
Discussions with audiologist(s)	Time since last visit; Whether talked about music; Who raised the topic; Whether discussions improved listening experiences

Note: For full survey questions and response options, see SM1.

*Demographics, engagement and preferences.* Participants were asked to provide demographic data (age, gender, and highest academic qualification). They were asked to indicate their level of engagement with music, which included the importance of music, frequency of listening, preference for listening to self-chosen music, having a large music collection, using music streaming services (e.g., Spotify), encouraging others to listen to music they like, and talking to others about music (Greasley, 2008; Greasley & Lamont, 2011). After this, they were asked to indicate whether they had received any musical training, worked in a musical field, and the impact of their HL on musical work. Preference for musical genres and functions of music were assessed using categories derived from prior research (e.g., Bonneville-Roussy et al., 2013; Schafer et al., 2013).

*Hearing.* Participants were asked about their hearing, including level, type, uni-, or bi-lateral, duration and age of onset. There is complexity and diversity of terminology around HL, with different terms preferred and commonly used. HL is used here, as it is the most frequently used term in scientific research papers to address the impact of different levels of hearing on music experience. HL level was measured in three ways. First, participants were asked to send an audiogram if they were willing and able to do so (data = Hd) and these were categorized according to the BSA 5-frequency average (BSA, 2018). Second, participants were asked to indicate how their audiologist had described their HL (memory = Hm). Third, by consulting national charities related to HL (RNID and BATOD) websites and our project advisory board, we derived subjective descriptors based on speech intelligibility in different contexts (e.g., one-to-one conversation, noisy environments) and asked participants to indicate which most accurately represented their experience without hearing aids (speech = Hs).

*Hearing aids and use*. Questions were asked about the make, model, and fitting of the users’ hearing aids (HA), whether they had a volume control or music program, and how often they used these in recorded and live music settings. This section included questions about modes of listening (e.g., through loudspeakers at home, using headphones, in the car), frequency of listening, types of listening events, and the difficulties experienced and strategies adopted to improve listening experiences.

*Audiology.* Participants were asked about discussions with audiologists, including when they last visited, if they had spoken about music listening, and whether this discussion had helped.

### Procedure

*Ethics.* Ethical clearance was granted by the University of Leeds Faculty of Arts, Humanities and Cultures Research Ethics Committee (PVAR 15-039) and the National Health Service (NHS) Research Ethics Committee (17 LO 2007 236452).

*Criteria for participation*. In order to participate in the research, participants were asked to indicate that: (a) they had a HL as identified by an audiologist; (b) they used HAs or Bone Anchored HAs (BAHA) for more than an hour a day; (c) they did not have a cochlear implant; and d) they were aged 18 years old or over.

*Recruitment*. The majority of participants were recruited via local, regional, and national mailing lists including the University of the Third Age, charities and organizations related to HL and deafness, music organizations, academic music departments and concert mailing lists (*n =* 1176). After gaining ethical approval from the Health Research Authority, a further 331 participants were recruited from 37 NHS Trusts across England (see SM2 for a list). Participants were offered the chance to enter a prize draw (with three prizes of GBP75) and to be informed of future studies.

*Data collection.* The survey was conducted between September 2016 and August 2018. A total of 72 participants used the BSL videos provided (6% of respondents).

### Analysis

Quantitative data and Likert scales were analyzed using SPSS28 and reported using descriptive and inferential statistics (ANOVA, non-parametric Kruskal-Wallis tests, chi square with Bonferroni correction). Where Likert scales indicated degree of agreement, scores were centered to highlight both agreement and disagreement (e.g., musical preferences, preferred musical characteristics, uses of music). Some Likert variables were converted to binary responses, comparing “yes” and “at least sometimes” to “no” and “never.”

To examine predictors of hearing-aid-related ratings (difficulties, helpfulness, and strategies), we employed cumulative link mixed models (CLMMs) using the *ordinal* package in R (Christensen, 2019). This approach allowed us to model ordinal responses across listening settings and HL severity groups while simultaneously accounting for stable individual differences via random intercepts for participants, as well as potential confounding factors such as age, gender, and level of musical training. Models were estimated with adaptive Gauss–Hermite quadrature (nAGQ = 5) to ensure accurate parameter estimation, and standard errors were computed from the Hessian matrix. All outcome variables were measured on 5-point ordinal scales, and missing data or “Don’t know” responses were excluded prior to analysis.

For each outcome domain, models were specified sequentially in a stepwise procedure. The baseline model included only listening item type as a fixed effect. HL severity and its interactions with item type were then added, followed by listening setting (recorded versus live) and participant age. Musical training and gender were subsequently added as predictors to evaluate their explanatory power and potential influence on the ratings. Model comparisons were conducted using likelihood-ratio tests, changes in akaike information criterion (AIC) and Bayesian information criterion (BIC), and pseudo-*R*² values (marginal and conditional). The intraclass correlation coefficient (ICC) was calculated to quantify the proportion of variance in ratings attributable to stable between-participant differences (e.g., general response tendencies, stable listening preferences or habits, underlying personal characteristics, or HA characteristics and usage). Only the final best-fitting models, that is, those with the greatest explanatory power while balancing model parsimony, are reported in the Results section. For full details of the stepwise model selection and fit statistics for each domain, please refer to SM3).

A thematic analysis of qualitative data from open-ended responses was conducted using NVivo 12 (QSR International Pty Ltd., 2018), with instances of recurrent themes identified and group differences in prevalence explored. A total of fourteen overarching themes emerged, with numerous sub-themes (see SM4). This paper uses a selection of the qualitative findings to illustrate quantitative findings and key trends. Qualitative theme counts (prevalence) for helpfulness, difficulties and strategies are not always mutually exclusive and may not be applicable to all individual participants.

## Results

### Sample Characteristics

*Demographics*. See Table 2. One thousand five hundred and seven hearing aid users began the survey. One thousand four hundred and twenty-five reached the section on HL measures. One thousand two hundred and eighty completed the entire survey. Of these, 131 people sent us their audiometric data. The age range was 18–95 years and the mean age was 60 (SD = 17) years (see Table 2 and SM5a). There was an even gender balance overall (49.6% female). After removing “Prefer not to say,” there were significantly more males in the 65–74 years and 75 + years age groups, and more females in the 25–44 years and 45–64 years age groups; χ²(4) = 81.37, *p* < .001, *V* = 0.23(see SM5b). Sixty-three percent had an undergraduate degree or higher. Ninety percent were based in the UK and Ireland, with a good spread of counties within England (*n =* 1,235, 29% North, 34% Midlands, and 37% South).

**Table 2.** Sample Demographics (*N =* 1507) Including Self-Reported Hearing Loss by Measure: Hs (Speech Descriptor) *n =* 1425, Hm (Memory) *n =* 1425, and Hd (Audiometric Data) *n =* 131.

Age (years) (*N =* 1507)	Mean (SD)	59.59 (16.97)
	Range	18–95
Gender (*N =* 1507)	Female	748 (49.6%)
	Male	755 (50.1%)
	Prefer not to say	4 (0.3%)
Educational level (*N =* 1507)	No qualification	104 (6.9%)
	GCSEs/O-Levels	179 (11.9%)
	A-Levels/diploma/baccalaureate	236 (15.7%)
	UG degree	519 (34.4%)
	TPG degree	334 (22.2%)
	PhD	103 (6.8%)
	Other	32 (2.1%)
Geographical Location (*n =* 1503)	UK	1365 (90.8%)
	Non-UK	138 (9.2%)
Geographical location (England) (*n =* 1235)	East Midlands	109 (8.8%)
	East of England	117 (9.5%)
	Greater London	136 (11.0%)
	North East England	59 (4.8%)
	North West England	132 (10.7%)
	South East England	197 (15.9%)
	South West England	123 (10.0%)
	West Midlands	194 (15.7%)
	Yorkshire and Humberside	168 (13.6%)
Hearing loss level (self-report speech descriptor) Hs (*n =* 1425)	Mild	313 (22.0%)
	Moderate	593 (41.6%)
	Severe	484 (33.9%)
	Profound	35 (2.5%)
Hearing loss level (memory of audiologist category) Hm (*n =* 1425)	Mild	90 (6.3%)
	Moderate	592 (41.6%)
	Severe	462 (32.4%)
	Profound	161 (11.3%)
	Don’t know	120 (8.4%)
Hearing loss level Audiometric data Hd (*n =* 131)	Normal	9 (6.9%)
	Mild	43 (32.8%)
	Moderate	51 (38.9%)
	Severe	23 (17.6%)
	Profound	5 (3.8%)

**Figure 1.**
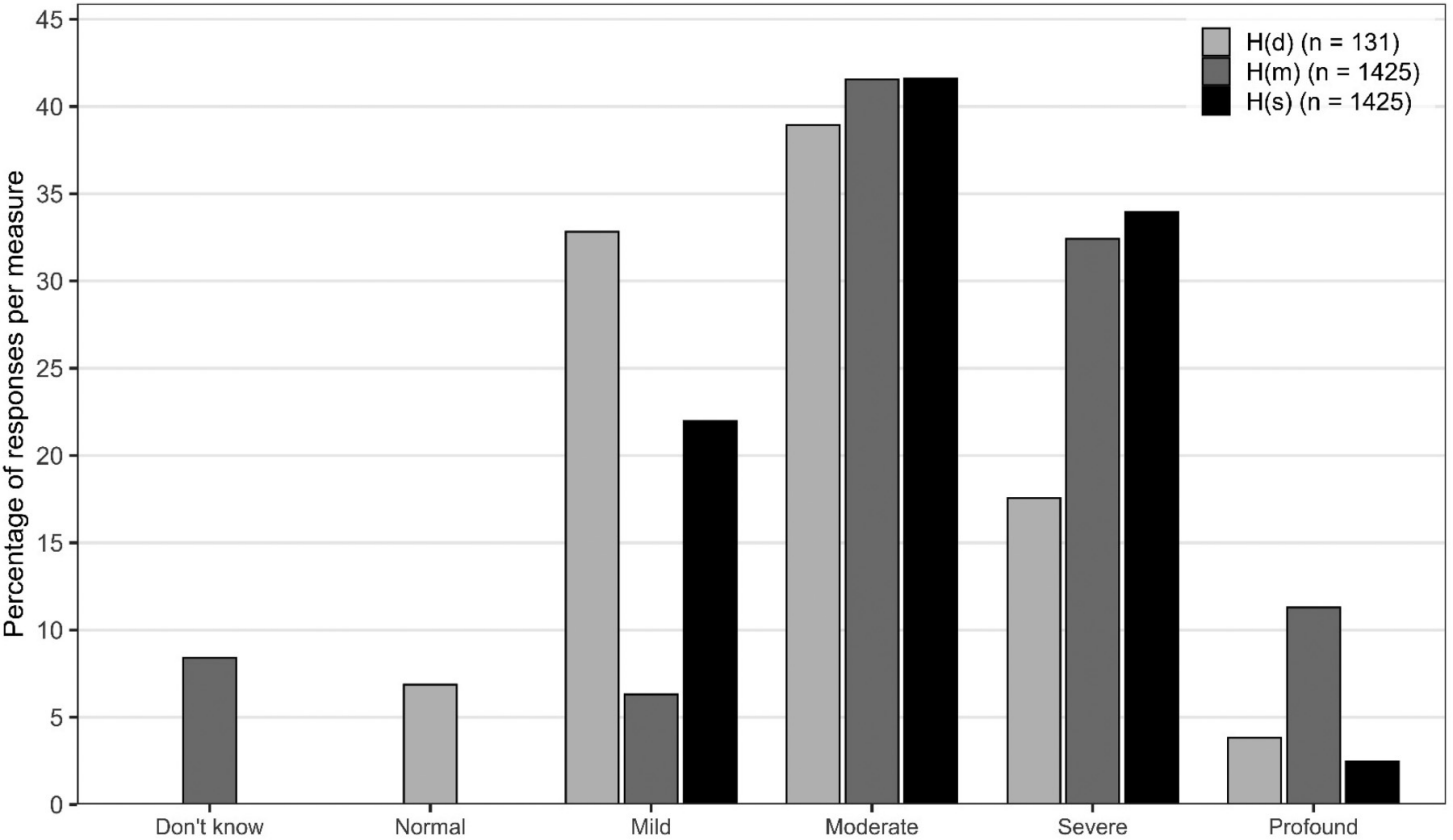
Prevalence of Hearing Loss Level for Each Measure: Audiometric Data Hd (*N =* 131); Memory of Reported Audiological Category, Hm (*N =* 1,425); and Subjective/Experiential/Speech Descriptor, Hs (*N =* 1,425).

*Hearing*. Ninety percent reported bilateral HL, of whom 13% had a HL since birth. Seventeen percent reported a sudden worsening of hearing while 41% reported a gradual deterioration. Twenty-seven percent reported presbycusis as the cause. The majority of participants responded to questions assessing HL in terms of the speech descriptor Hs (*n =* 1425) and their memory of the hearing level described by their audiologist Hm (*n =* 1425). Additionally, for those who submitted an audiogram Hd (*n =* 131), the BSA “best ear” categorization resulted in nine participants being categorized as “normal” due to asymmetrical HL. For those who had provided data for all three HL measures (*n =* 113), agreement between measures was calculated by determining the number of exact category matches, first between Hd and Hs, and then between Hd and Hm, excluding those who reported “Do not know” for Hm. There was 43.4% agreement between Hd and Hm (*n =* 49/113) and 49.6% agreement between Hd and Hs (*n =* 56/113). Therefore Hs (*n =* 1425) was used as the measure for exploring differences according to hearing level. Figure 1 shows prevalence for each hearing level category.

*Musical training*. Three musical training items (musical activities, music educational qualifications, instrumental performance history) were summed to create a scale between 0 and 20. A majority scored between 0 and 4 out of 20, with nearly 20% (*n =* 297) having no musical training at all (see SM6). Around a quarter (24%) had worked in a musical field.

**Figure 2.**
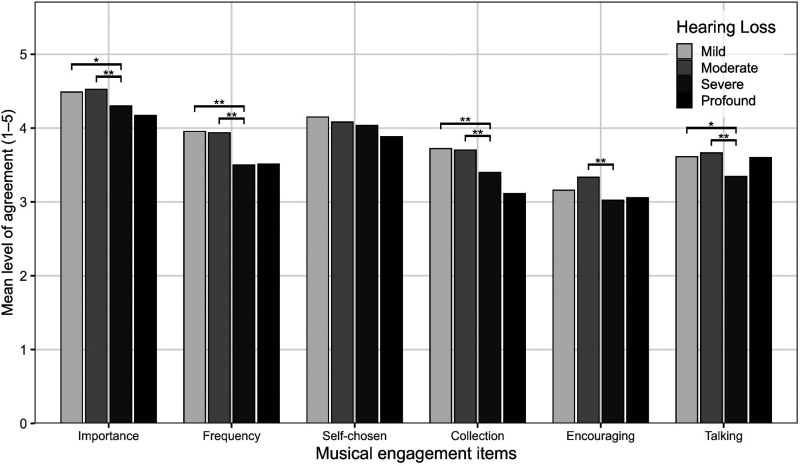
Level of Agreement with Musical Engagement Items for Each Hearing Loss Level (*N* = 1,425, Mild *n* = 313, Moderate *n = 593*, Severe *n* = 484, Profound *n = 35*, **p < .001, *p < .05). Items: *Importance* of Music, *Frequency* of Listening, Preference for s*elf-chosen* Music, Size of Music *collection*, *Encouraging* Others to Listen to Music, *Talking* with Others About Preferred Music.

*Musical engagement*. The vast majority (87%) of participants agreed or strongly agreed that music was very important to them, and most indicated that they listened to music as often as possible (67%) and preferred to listen to self-chosen music (77%) (see SM7a). However, 61% disagreed that they listen through music streaming services (e.g., Spotify) and this item did not load highly on engagement as a factor through a PCA analysis, so this item was not carried through to the overall measure and was not analyzed further. Only 0.007% (*n =* 11) strongly disagreed with all six items (see SM7b). A six-item measure of musical engagement was computed to examine effects of HL level (items scores were summed to give individual overall scores, and then group averages were calculated). Overall, engagement was high for all participants, but those with severe HL were significantly less likely to be engaged with music than those with mild or moderate HL (*H*(3) = 34.72, *p* < 0.001, η^2^ = 0.02, Table 3 §1, Figure 2, SM7c). A third (34%) never avoided listening to music but there was a spread of responses: never 34%; occasionally 18%; sometimes 25%; often 15% and all the time 8%. Those with severe HL were more likely to avoid listening to music (*χ*^2^(12) = 157.15, *p* < .001, *V* *=* 0.19, see Table 3 §2 and SM7d).

**Table 3.** Effects of Hearing Loss (HL) Level on Survey Variables.

§	Variable	Total N	Finding	Statistic	Group differences
1	Musical engagement	*n = *1425	Higher HL less likely to be engaged in music listening.	*H*(3) = 34.724, *p* < .001, η^2^ = 0.02	Sev < Mild, *p < .*001 Sev < Mod, *p < .*001
2	Avoid music listening	*n =* 1425	Higher HL more likely to avoid.	χ^2^(12) = 157.149, *p* < .001, *V* *=* 0.19	Linear association with HL level.
3	Musical preferences (for styles listened to)	*choral n =* 1259 *chamber* *n =* 1275 *orchestral n =* 1303 *opera n =* 1267	Higher HL lower preference for classical styles.	*choral * *F*(3, 1259) = 4.241, *p* < .005, η^2^ = 0.01 *classical (chamber) * *F*(3,1275) = 4.272, *p* < .005, η^2^ = 0.009 *classical (orchestral)* *F*(3, 1303) = 4.989, *p* < .002, η^2^ = 0.01 *opera* *F*(3, 1267) = 2.947, *p* < .032, η^2^ = 0.006	Sev < Mod (*p < .*005) Sev < Mod (*p < .*010) Sev < Mod (*p < .*003), Sev < Mild (*p < .*03) Sev < Mod (*p = .*0495)
4	Importance of musical features	*n =* 1425	Higher HL less likely to rate *voice*, *instrumentation* and *harmony* as important for music appreciation. No significant effects of HL for lyrics, rhythm, tempo, dynamics.	*voice* *H*(3) = 13.956, *p = .*003, η^2^ = 0.006 *instrumentation* *H*(3) = 10.887, *p = .*012, η^2^ = 0.004 *harmony* *H*(3) = 37.121, *p < .*001, η^2^ = 0.02	Sev < Mod *p = .*002 Sev < Mod *p = .*011 Sev + Prof < Mild + Mod (*p < .*05)
5	Uses of music	*n =* 1425	Higher HL less likely to listen to music for this reason. Higher HL more likely to use music to help with tinnitus.	*pleasure* *H*(3) = 9.499, *p = .*023, η^2^ = 0.003 *other people's pleasure* *H*(3) = 8.515, *p = .*036, η^2^ = 0.002 *create right atmosphere* *H*(3) = 10.295, *p = .*016, η^2^ = 0.004 *concentration* *H*(3) = 9.331, *p = .*025, η^2^ = 0.003 *help with work* *H*(3) = 25.576, *p < .*001, η^2^ = 0.01 *to help with tinnitus* *H*(3) = 10.715, *p = .*01, η^2^ = 0.004	Sev < Mod, *p = .*040 Sev < Mod, *p = .*030 Sev < Mod, *p = .*021 Sev < Mod, *p = .*029 Sev < Mod, *p < .*001 Sev > Mild, *p = .*013
6	One or two hearing aids	*n =* 1409	Higher HL more likely to wear 2 HAs.	χ^2^(3) = 26.828, *p < .*001, *V* = 0.14	Mild > Sev to wear 1HA Sev > Mild to wear 2HA
7	Type of HA	*n =* 1392 (excludes don’t know)	No association between HL and type of HA worn.	χ^2^ (12) = 7.346, *p* = .834, *V* = 0.04	
8	HA fitting (open/closed)	*n =* 1332 (excludes don’t know)	Higher HL more likely to wear closed dome fitting.	χ^2^(9) = 319.48, *p < .*001, *V* *=* *0.28*	Mild + Mod > open Prof > closed
9	Duration of HA use	*n =* 1409	Higher HL more likely to be wearing for longer.	χ^2^(15) = 223.85, *p < .*001, *V* *=* *0.23*	Mild < to wear 5 + years Sev + Prof > wear 5 + years
10	Duration of current HA use	*n =* 1409	Higher HL more likely to be wearing for longer.	χ^2^(15) = 47.103, *p < .*001, *V* *=* *0.11*	Mild > less than 3 months Sev + Prof > 5 + years
11	Music program	*n =* 1310 (excludes don’t know)	Higher HL more likely to have music program.	χ^2^(3) = 18.848, *p < .*001, *V* *=* *0.12*	Mild < Mod + Sev + Prof
12	Music program use (those who have a MP)	*n =* 480	No significant effects of HL.	χ²(12) = 21.836, *p = .*039, *V* *=* *0.12* (NB: not sig. with Bonferroni correction)	
13	Overall helpfulness of HAs in REC and LIVE	REC *n =* 1119 LIVE *n =* 931	No significant effects of HL.	REC *H*(3) = 0.761, *p = .*859, η^2^ = -0.004 LIVE *H*(3) = 6.738, *p = .*081, η^2^ = 0.002	Trends towards higher HL levels report HAs less helpful in live settings.
14	Listen to recorded music with HAs	*n =* 1406	Higher HL less likely to listen to recorded music.	χ^2^(9) = 40.222, *p < .*001, *V* *=* *0.09*	Mild + Mod > Sev
15	Time spent listening to recorded music (Recoded 4 groups: 0–2, 3–4, 5–10, 10 + hours)	*n =* 1119	Mild and moderate HL likely to listen longer than severe HL.	χ^2^(9) = 28.623, *p < .*001, *V* *=* *0.09*	Mild + Mod > Severe
16	Mode of listening to recorded music*	*n =* 1119	Higher HL less likely to be listening through *car stereo* or via *headphones*. No effect of HL for *loudspeakers*.	*car stereo* χ^2^(3) = 15.205, *p = *.002, *V* = 0.12 *headphones* χ^2^(3) = 12.259, *p < .*007, *V* = 0.11 *loudspeakers* χ²(3) = 1.130, *p* = .770, *V* = 0.03	*car stereo* Mild + mod > Sev + Prof *headphones* Mild + mod > Sev + Prof
17	Listening to recorded music with HAs*	*car n =* 934 *headphones n =* 699 *loudspeakers n =* 1055 (NB: removing “I don’t listen in this setting”)	Higher HL more likely to wear HAs when listening via *headphones and through loudspeakers.*	*car stereo* χ²(3) = 7.873, *p = .*049, *V* = 0.09 (NB: not sig. with Bonferroni correction) *headphones* χ²(3) = 22.413, *p < .*001, *V* = 0.18 *loudspeakers* χ²(3) = 13.079, *p = .*004, *V* = 0.11	For all: Sev + Prof > Mild + Mod
18	Helpfulness of HAs (REC)	*lyrics n =* 1068 *instruments n =* 1041 (excludes don’t know)	Higher HL less likely to report HAs helpful for *hearing lyrics* and *picking out instruments*. No effects of HL for *hearing the melody*, *bassline* or *singer*.	*hearing lyrics* *H*(3,1068) = 56.745, *p < .*001, η^2^ = 0.05 *picking out instruments* *H*(3, 1041) = 52.997, *p < .*001, η^2^ = 0.05	*hearing lyrics* Mild + Mod > Sev + Prof, *p < .*001 *picking out instruments* Mild + Mod > Sev + Prof, *p < .*001
19	Difficulties (REC)*	*distortion n =* 1049 *too much bass n =* 974 (excludes “Don’t know”)	Higher HL more likely to experience *distortion* and *too much bass*. No effects of HL found for *too much treble*, *feedback*, *sudden changes in loudness*, *discomfort from loud sounds*.	*distortion* χ^2^(3) = 20.386, *p < .*001, *V* = 0.14 *too much bass* χ^2^(3) = 41.867, *p < .*001, *V* = 0.21	*distortion* (At least occasionally) Mild < Mod + Sev + Prof *too much bass* Mild + Mod < Sev + Prof
20	Strategies (REC)*	*adjust volume n =* 1046 *change program n =* 1066 (excludes my HA does automatically) *move away n =* 1119	Higher HL more likely to adopt all strategies.	*adjust volume* χ^2^(3) = 18.718, *p < .*001, *V* = 0.13 *change program* χ^2^(3) = 15.023, *p = .*002, *V* = 0.12 *move away from sound source* χ^2^(3) = 13.797, *p = .*003, *V* = 0.11	*adjust volume* Mild < Sev *change program* Mild + Prof < Sev *move away* Mild < Mod + Sev
21	Attending live events with HAs*	*n =* 1134 (excluding “N/A I don’t attend live events”)	Higher HL more likely to wear HAs when attending live events.	χ²(3) = 17.329, *p < .*001, *V* = 0.12	Mild + Mod < Sev
22	Number of live events attended (recoded into 0–2, 3–6, 7 + per annum)	*n =* 931	No association between HL level and number of live events attended.	χ²(6) = 11.246, *p = .*081, *V* = 0.08	
23	Type of live events attended (recoded into 100% acoustic, mixed and 100% amplified)	*n =* 931	No association between HL and type of live event (acoustic or amplified).	χ² (6) = 6.386, *p = .*381, *V* = 0.06	
24	HA use in live settings*	*acoustic* *n =* 835 *amplified n =* 775 *playing n =* 626 (excluding “N/A”)	Higher HL more likely to wear HAs in amplified settings and when playing an instrument.	*acoustic* χ^2^(3)= 1.336, *p = .*721, *V* = 0.04 *amplified* χ^2^(3) = 10.781, *p = .*013, *V* = 0.12 *playing an instrument* χ^2^(3) = 14.376, *p* = .0002, *V* = 0.15	*amplified* Mod < Sev + Prof *playing an instrument* Mod < Sev
25	Helpfulness of HAs (LIVE)	*melody n =* 888 *singer* *n =* 891 *lyrics* *n =* 892 *instruments n =* 869	Higher HL less likely to report HAs helpful for *hearing the melody, singer and lyrics*. No effects of HL for *hearing the bassline.*	*hearing the melody* *H*(3,888) = 13.790, *p = .*003, η^2^ = 0.009 *hearing the singer* *H*(3,891) = 23.422, *p < .*001, η^2^ = 0.02 *hearing lyrics* *H*(3,892) = 71.797, *p < .*001, η^2^ = 0.07 *picking out instruments* H(3, 869) = 35.083, *p < .*001, η^2^ = 0.03	*hearing the melody* Mild + Mod + Sev > Prof, *p = .*003 *hearing the singer* Mild + Mod + Sev > Prof, *p = .*001 *hearing lyrics* Mild + Mod + Sev > Prof, *p = .*001 *picking out instruments* Mild + Mod > Sev + Prof, *p = .*001
26	Difficulties (LIVE)*	*distortion* *n =* 884 *bass* *n =* 829 *Discomfort* *n =* 895 (excludes “Don’t know”)	Higher HL levels more likely to experience difficulties with *distortion* and *too much bass*. No effects found for difficulties with *too much treble*, *feedback*, *sudden changes in loudness*.	*distortion* χ^2^(3) = 30.044, *p < .*001, *V* *=* 0.18 *too much bass* χ^2^(3) = 29.487, *p < .*001, *V* = 0.19 *discomfort from loud sounds* χ^2^(3) = 10.645, *p = .*013, *V* = 0.11	*distortion* Mild + Mod < Sev + Prof *too much bass* Mild + Mod < Sev + Prof *discomfort from loud sounds* Mild < Mod
27	Strategies (LIVE)*	*adjust volume n =* 857 (excludes my HA does automatically)	Higher HL more likely to *adjust volume.* No effects of HL for *changing program* or *moving away from sound source*.	*adjust volume* χ^2^(3) = 12.936, *p = .*005, *V* = 0.12	*adjust volume* Mild + Mod < Sev
28	Visited audiologist (recoded into within 6 months’ and six months ago or more)	*n =* 1,300	No significant effects of HL on time since last visit to audiologist.	χ^2^(3) = 7.622, *p = .*054, *V* = 0.08	
29	Whether have discussed music with audiologist.	*n =* 1,300	Higher HL more likely to have discussed music.	χ^2^(3) = 14.933, *p = .*002, *V* = 0.11	Mild < Mod + Sev
30	Whether discussion helpful.	*n =* 764	No significant effects of HL on helpfulness of discussion.	χ^2^(9) = 9.613, *p = .*383, *V* = 0.06	

*Note.* Hs descriptor used. Bonferroni correction applied to chi-square analyses.

Asterisk (*) denotes where a binary variable has been used “never” versus “at least sometimes.”

*Musical preferences and functions*. The strongest preferences were for classical orchestral, classical chamber, blues, musical theatre, and rock, while hip-hop/rap and heavy metal were rated the lowest (see SM8a). Those with severe and profound HL reported lower preferences for choral (*F*(3, 1259) = 4.24, *p* < .005, η^2^ = 0.01), classical chamber (*F*(3, 1275) = 4.27, *p* < .005, η^2^ = 0.009), classical orchestral (*F*(3, 1303) = 4.99, *p* < .002, η^2^ = 0.01) and opera (*F*(3, 1267) = 2.95, *p* < .032, η^2^ = .006) styles than those with mild or moderate HL (Table 3 §3 and SM8b). Seven out of eight musical features were rated as important in the enjoyment of music: voice, rhythm, harmony, instrumentation, tempo, lyrics, and dynamics, in order of importance. Synthesizers were not rated as important (see SM8c). Three features were rated as less important by those with higher levels of HL: voice (*H*(3) = 13.96, *p* = .003, η^2^ = 0.006), instrumentation (*H*(3) = 10.89, *p* = .012, η^2^ = 0.004) and harmony *H*(3) = 37.12, *p* < .001, η^2^ = 0.02) (Table 3 §4). Participants reported listening to music mainly for pleasure, relaxation, mood regulation and reminiscence, but mostly not to help with tinnitus or for work. Reports of using music for concentration and exercise were neutral (mid-way between agree and disagree) (Table 3 §5). Those with severe HL used music less for pleasure (*H*(3) = 9.50, *p* = .023, η^2^ = 0.003), other people's pleasure (*H*(3) = 8.52, *p = .*036, η^2^ = 0.002), creating the right atmosphere (*H*(3) = 10.30, *p = .*016, η^2^ = 0.004), concentration (*H*(3) = 9.33, *p = .*025, η^2^ = 0.003) or to help with work (*H*(3) = 25.58, *p* < .001, η^2^ = 0.01) than those with moderate HL. However, those with severe HL were more likely to use music to help with tinnitus than those with mild HL (*H*(3) = 10.72, *p = .*01, η^2^ = 0.004).

*Hearing Aids*. Ninety percent of the sample reported wearing two HAs and, of these, 90% reported wearing BTE HAs, with the remaining 10% split between other types of HAs (e.g., RTE, ITC, CIC, BCHD). Sixty-seven percent had worn HAs for more than five years. There was variability in how long they had used their current HAs, with around a third having worn them for up to 12 months (31%), for 1–2 years (30%) and for three or more years (38%). Sixty-five percent of the sample wore open dome fittings (see SM9). As expected, those with more severe HL were more likely to wear two HAs (χ^2^(3) = 26.83, *p* < .001, *V* = 0.14), to wear a closed (occluded) fitting (χ^2^(9) = 319.48, *p* < .001, *V* = 0.28), and to have been wearing HAs for longer (χ^2^(15) = 223.85, *p* < .001, *V* = 0.23) (Table 3 §6–10). Across all levels of HL, 72% wore HAs manufactured by Phonak or Oticon, reflecting trends in standard NHS provision in England. Remaining participants wore HAs made by Resound, Siemens, Widex, or Starkey, and a further nine smaller manufacturers. Roughly a third (34%) reported that they had a music program available on their HA (*n =* 484). Of those who did have a music program, when listening to music 10% used the program “all the time,” 35% “often,” 25% occasionally, 16% sometimes and 14% never (see SM10). Those with more severe HL were more likely to have a music program (χ^2^(3) = 18.85, *p* < .001, *V* = 0.12). However, there was no significant association between HL level and music program use when Bonferroni correction was applied (χ²(12) = 21.84, *p = .*039, *V* = 0.12)(Table 3 §11–12).

### Usage of Hearing Aids in Recorded and Live Music Settings

#### Recorded Music Settings

A majority (84%) reported using HAs for recorded music at least sometimes, while 16% either did not listen at all (4%) or did not wear HAs when listening (12%) (see Table 4 and SM11). Those with mild and moderate HL were more likely to listen to recorded music with HAs than those with severe HL (χ^2^(9) = 40.22, *p* < .001, *V* *=* 0.09). There was a spread in the amount of time spent listening to music, from <3 to 10 + hours per week. Those with mild and moderate HL were more likely to listen for longer periods than those with severe HL (χ^2^(9) = 28.62, *p* < .001, *V* = 0.09) (Table 3 §14–15). Participants commonly reported listening to recorded music via loudspeakers (88%) or car stereo (85%), while fewer listened via headphones (61%). Almost all wore HAs when listening via loudspeakers (97%) or in a car (94%), but only 55% wore them with headphones, most likely for practical reasons. Those with severe and profound HL were more likely to listen with their HAs when listening through loudspeakers (χ²(3) = 13.08, *p = .*004, *V* = 0.11) and using headphones (χ²(3) = 22.41, *p* < .001, *V* = 0.18) (see Table 3 §16–17).

**Table 4.** Use of Hearing Aids for Listening to Recorded Music.

Use of HAs for recorded music (*n =* 1406)	Yes	878 (62.5%)
	Sometimes	297 (21.1%)
	No	173 (12.3%)
	N/A, don’t listen to recorded music	58 (4.1%)
Hours listening to recorded music (*n =* 1119)	None or 1–2 h	314 (30.5%)
	3–4 h	279 (24.9%)
	5–10 h	256 (22.9%)
	More than 10 h	243 (21.7%)
Listening with hearing aids (*n =* 1119)		
…via loudspeakers	Never	140 (12.5%)
	Occasionally	176 (15.7%)
	Sometimes	226 (20.2%)
	Frequently	379 (33.9%)
	All the time	198 (17.7%)
…via headphones	Never	440 (39.3%)
	Occasionally	202 (18.1%)
	Sometimes	194 (17.3%)
	Frequently	215 (19.2%)
	All the time	68 (6.1%)
…via car stereo	Never	174 (15.6%)
	Occasionally	191 (17.1%)
	Sometimes	217 (19.4%)
	Frequently	395 (35.3%)
	All the time	142 (12.6%)

#### Live Music Settings

Almost three-quarters of the sample (72%) reported attending live music events at least sometimes (see Table 5 and SM12). There was no association between frequency of attendance and HL level (χ²(6) = 11.25, *p = .*081, *V* = 0.08), but those with greater HL were more likely to wear HAs when attending live events (χ²(3) = 17.33, *p* < .001, *V* = 0.12)(see Table 3 §21–27). Roughly half of respondents (47%) reported listening to both unamplified and amplified live music, while the remainder split relatively evenly between listening to unamplified (25%) and amplified (28%) music. The remainder (28%) either did not listen to live music at all (16%) or did not use their HAs when doing so (12%). There was no significant association between HL level and type of live event attended (χ²(6) = 6.39, *p = .*381, *V* = 0.059). Of those who did attend live events (*n =* 931), the majority reported using their HAs at least sometimes at live unamplified events (98%), live amplified events (93%) and when playing an instrument/singing (93%). For example, of the 30% (*n =* 598) who currently play an instrument, 93% of those played an instrument whilst wearing HAs at least sometimes. Those with greater HL were more likely to wear HAs in amplified settings (χ^2^(3) = 10.78, *p = .*013, *V* = 0.12) and when playing an instrument/singing (χ^2^(3) = 14.38, *p = .*002, *V* = 0.15).

**Table 5.** Use of Hearing Aids in Live Music Settings.

Wear hearing aids at live events		Overall (*n =* 1345)
	Yes	746 (55.5%)
	Sometimes	222 (16.5%)
	No	166 (12.3%)
	N/A	211 (15.7%)
Number live events in last 12 months		Overall (*n =* 931)
	0, 1 or 2	319 (34.3%)
	3 to 6	304 (32.6%)
	7 or more	308 (33.1%)
Type of live events		Overall (*n =* 931)
	100% acoustic	231 (24.8%)
	100% amplified	260 (27.9%)
	Both	440 (47.3%)
Listen…		
to *live acoustic music*		Overall (*n =* 837)
	With HAs	648 (77.4%)
	Sometimes with HAs	175 (20.9%)
	Without HAs	14 (1.7%)
to *live amplified music*		Overall (*n =* 777)
	With HAs	516 (66.4%)
	Sometimes with HAs	210 (27.0%)
	Without HAs	51 (6.6%)
*when playing an instrument/singing*		Overall (*n =* 628)
	With HAs	451 (71.8%)
	Sometimes with HAs	134 (21.3%)
	Without HAs	43 (6.9%)

### Helpfulness of Hearing Aids in Recorded and Live Settings

We analyzed rated helpfulness of HAs across different listening dimensions (hearing the bass line, melody, singer, lyrics, picking out instruments) in recorded and live settings (see SM13–14 for descriptive and inferential statistics). Participants most often rated their HAs as “Very helpful” (modal response) across all listening dimensions in recorded settings. Ratings were also high for live settings with the modal responses being “Very helpful” or “Fairly helpful” for most listening dimensions, with the exception of Lyrics, where the modal response was only “Somewhat helpful.” However, the *overall* rated helpfulness of HAs in live music settings (mean score 6.5/10) was slightly but significantly less than for recorded settings (mean score 6.8/10) (see SM13).

The CLMM for helpfulness ratings included items for five listening dimensions (Melody, Bass, Singer, Lyrics, Instruments), combining responses from recorded and live music listening settings. The final sample yielded 877 respondents contributing 8,245 item-level observations. The inclusion of age (ΔAIC ≈  + 12, χ²(1) = 0.13, *p = .*72), gender (ΔAIC ≈ + 2, χ²(2) = 3.72, *p = .*29), or musical training (ΔAIC ≈ + 2, χ²(2) = 2.63, *p = .*27) did not improve the model fit (see SM3 Table 1a for details on the model selection). Accordingly, the final model (H3) included item type, HL severity and its interaction with item type (ΔAIC ≈ −239, χ²(15) = 268.82, *p* < .001), and listening setting (ΔAIC ≈ −67, χ²(1) = 69.25, *p* < .001). This model (see SM3 Table 1b for model specifications) explained 65.2% of the total variance in helpfulness ratings (marginal *R*² = 0.049), with an ICC of 0.63, indicating that a substantial proportion of the variance was attributable to individual differences.

**Figure 3.**
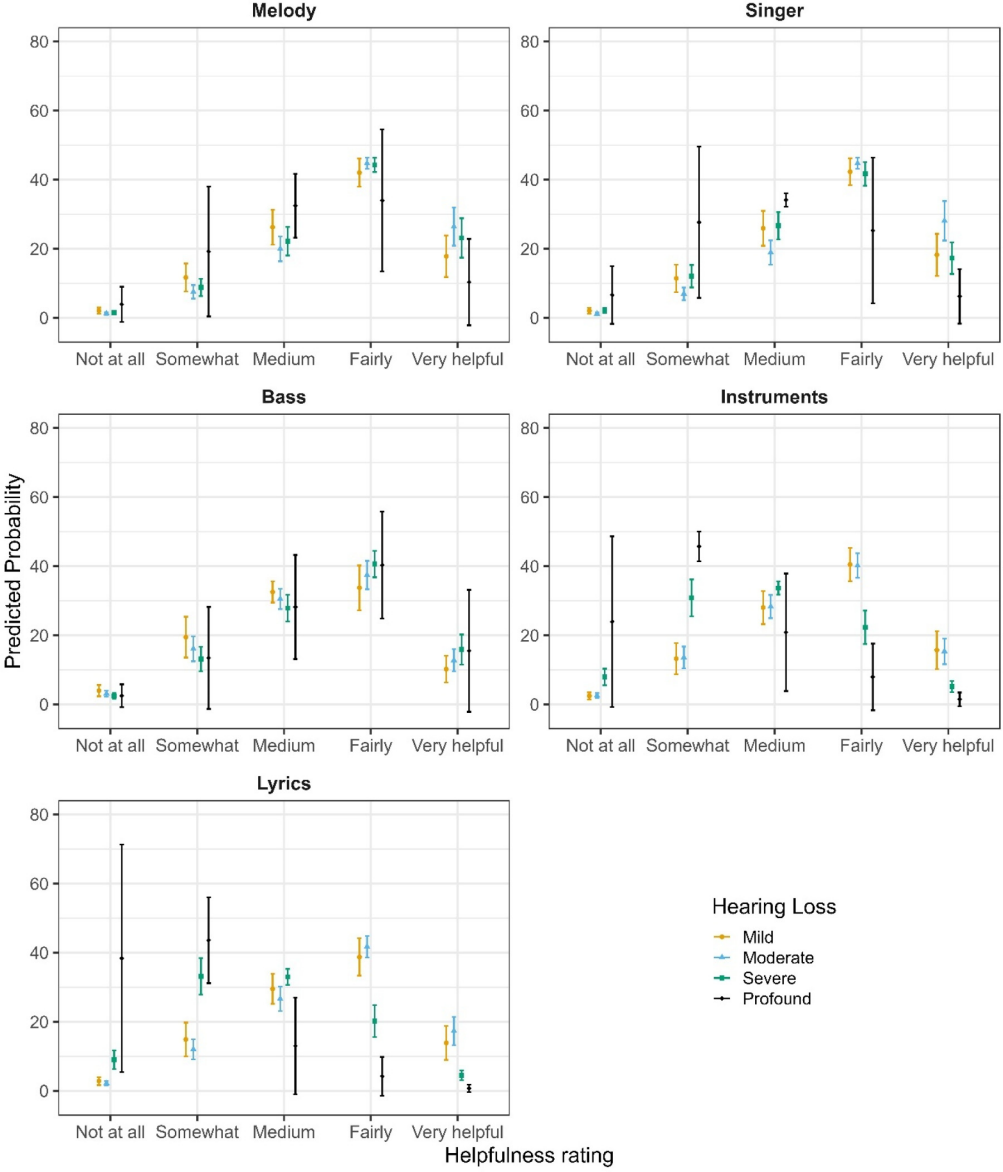
Predicted Probabilities of Self-Reported Helpfulness Ratings for All Items for Each Category of Hearing Loss.

**Figure 4.**
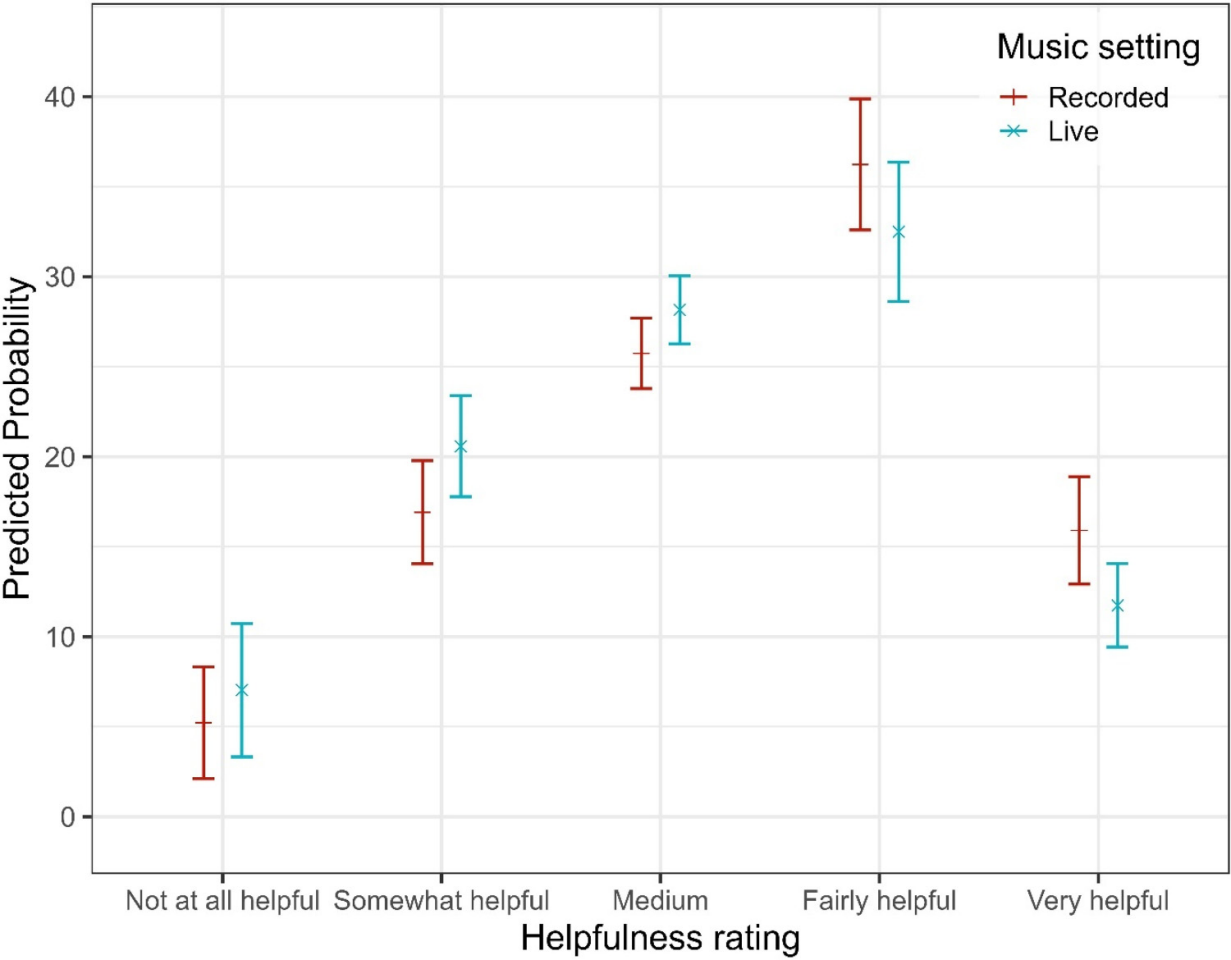
Predicted Probabilities of Self-Reported Helpfulness Ratings for Each Listening Setting.

In the final model, notable differences emerged between items: relative to the reference category (Bass), participants reported significantly lower helpfulness for “Hearing out instruments” (OR = 0.47, 95% CI [0.36, 0.61], *p* < .001) and for Lyrics (OR = 0.38, 95% CI [0.29, 0.50], *p* < .001). Melody was perceived as more helpful than Bass (OR = 1.48, 95% CI [1.14, 1.91], *p = .*003), while Singer did not differ significantly from Bass (OR = 1.21, 95% CI [0.93, 1.57], *p = .*158). The effect of HL severity on perceived helpfulness differed markedly across items. Pairwise comparisons of estimated marginal means (averaged over items and listening settings) revealed significant overall effects of HL severity. Compared to participants with mild HL, those with moderate (estimate = −0.47, SE = 0.15, z = −3.08, *p = .*011) and severe HL (estimate = −0.74, SE = 0.16, z = −4.67, *p* < .001) reported significantly lower helpfulness overall. No significant differences were observed for any other contrasts (*p* > .14). However, the effect of HL on helpfulness ratings was highly dependent on the listening dimension (see Figure 3). While a modest linear HL effect was observed for Bass (OR = 1.46, 95% CI [0.56, 3.80], *p = .*435), the helpfulness gain associated with increasing HL severity was substantially attenuated for Instruments (OR = 0.06, 95% CI [0.05, 0.19], *p* < .001) and Lyrics (OR = 0.06, 95% CI [0.03, 0.12], *p* < .001), corresponding to reductions of approximately 90–94% in OR relative to Bass. Melody (OR = 0.43, 95% CI [0.22, 0.83], *p* = .013) and Singer (OR = 0.26, 95% CI [0.13, 0.52], *p* < .001) also showed significantly reduced HL effects, though to a lesser degree. In addition to these linear effects, several non-linear HL trends were observed. Significant quadratic (curvilinear) HL effects emerged for Lyrics (OR = 0.40, 95% CI [0.23, 0.70], *p = .*001), Melody (OR = 0.55, 95% CI [0.33, 0.93], *p = .*026), and Singer (OR = 0.43, 95% CI [0.22, 0.84], *p = .*013), indicating that helpfulness ratings tended to plateau or even decline at higher degrees of HL severity. For Lyrics, a significant cubic HL effect (OR = 1.46, 95% CI [1.06, 2.01], *p = .*020) suggested further non-linear variation across the severity spectrum. No significant quadratic or cubic effects were observed for Bass, and higher-order effects for melody and singer were nonsignificant. Beyond item- and HL-related effects, listening setting showed a consistent main effect: live music was associated with a 31% reduction in the odds of reporting higher helpfulness ratings compared to recorded listening (OR = 0.69, 95% CI [0.63, 0.75], *p* < .001), indicating that participants perceived their HAs as less helpful in live music contexts (see Figure 4).

Predicted Probabilities of Self-Reported Helpfulness Ratings, Estimated From a CLMM of Model H3, Holding Age Constant at the Sample Mean and Averaging Across Listening Settings. Hearing Loss (HL) Severity Groups Are Displayed With Different Colors and Symbols: Mild (Orange Circles), Moderate (Blue Triangles), Severe (Green Squares), and Profound (Black Diamonds). Helpfulness Ratings Ranged from *Not at all helpful* to *Very helpful*. Error Bars Indicate 95% Confidence Intervals.

Predicted Probabilities of Self-Reported Helpfulness Ratings, Estimated From a CLMM of Model H3, Holding Age Constant at the Sample Mean and Averaging Across Hearing Loss Groups and Items. Music Settings Are Displayed with Different Colors and Symbols: Recorded (red Pluses) and Live (Blue Crosses). Helpfulness Ratings Ranged from *Not at all helpful* to *Very helpful*. Error Bars Indicate 95% Confidence Intervals.

#### Qualitative Data on Helpfulness

A total of 180 qualitative responses were coded for the helpfulness of HAs for music across listening settings (see Table 6 and SM4). Many (62) were general statements, but some (30) related to music programs, or picking out musical elements such as higher notes or specific instruments (14). Some (14) reported that they would not be able to listen to music at all without HAs. A few (12) referred to the helpful use of telecoil-loops and adjustments by audiologists.

**Table 6.** Helpfulness of Hearing Aids for Music (Qualitative Theme).

Theme	Count	Illustrative quotations
Generally helpful	62	*“Overall my hearing aids are helpful; I am aware of hearing a wider spectrum of sound.”*
Music program helpful	30	*“The music programme added to my hearing aid has made all the difference to my enjoyment of live music and participation in choral singing.”*
Cannot hear music without HAs	14	*“Without hearing aids I wouldn't be able to listen to music at all.”*
Hear out musical elements	14	*“Can hear higher notes, and individual voices/instruments much more clearly with aids.”*
Helpful if combined with T Loop	12	*“Venues often have a loop which is a great help.”*
Helpful with additional adjustments by audiologist	12	*“My audiologist altered the settings on multiple occasions on the music setting on my hearing aids until a satisfactory sound was arrived at.”*
No difficulties	12	*“No difficulties at all.”*
Streaming and phone app helpful	9	*“I was fitted with wireless hearing aids and bought a streaming device. This helped.”*
Helpful as long as music not too loud	7	*“Hearing aid is fine as long as it is not overloaded.”*
Helpful for performance	7	*“As I play in a brass band I have to ensure that I am playing the right music at the right time. My hearing aids help with this.”*
Helpful if music is not complex	1	*“One person with no musical accompaniment is absolutely fine using my hearing aids.”*

### Difficulties Experienced in Recorded and Live Music Settings

We explored difficulties experienced in recorded and live settings (see SM13 for descriptives). In recorded music settings, the modal (most frequent) response for five of six difficulties queried was that they were “Never” experienced: Too much bass, Too much treble, Feedback, Sudden changes in loudness and Discomfort from loud sounds. However, 80% reported experiencing Distortion at least occasionally. The modal response across all difficulties in live settings was also that participants “Never” experienced difficulties. However, Distortion was most frequently reported as “Sometimes” (see SM13).

To assess how participants experienced HA-related difficulties, the CLMM included six listening dimensions (Bass, Distortion, Feedback, Treble, Volume Changes, Discomfort). The CLMM analysis included a final sample of 879 respondents and 9,736 item-level observations. Likelihood-ratio testing indicated that the inclusion of HL severity and its interaction with item type (ΔAIC ≈ −88, χ²(18) = 124.06, *p* < 0.001), music listening setting (ΔAIC ≈ −26, χ²(1) = 28.23, *p* < 0.001), and age (ΔAIC ≈ −25, χ²(1) = 26.97, *p* < 0.001) significantly improved model fit; whereas adding gender (ΔAIC ≈ −1, χ²(2) = 4.60, *p = .*10) or musical training (ΔAIC ≈  + 1, χ²(1) = 0.22, *p = .*64) did not (see SM3 Table 2a for details on model selection). Accordingly, the final model (D4) included item type, HL severity and its interaction with item type, listening setting, and age. This model explained 46.6% of the variance in difficulty ratings (marginal *R*² = .075). The moderately high ICC (ICC = 0.42) indicated that a substantial proportion of the variance was attributable to stable between-participant differences.

**Figure 5.**
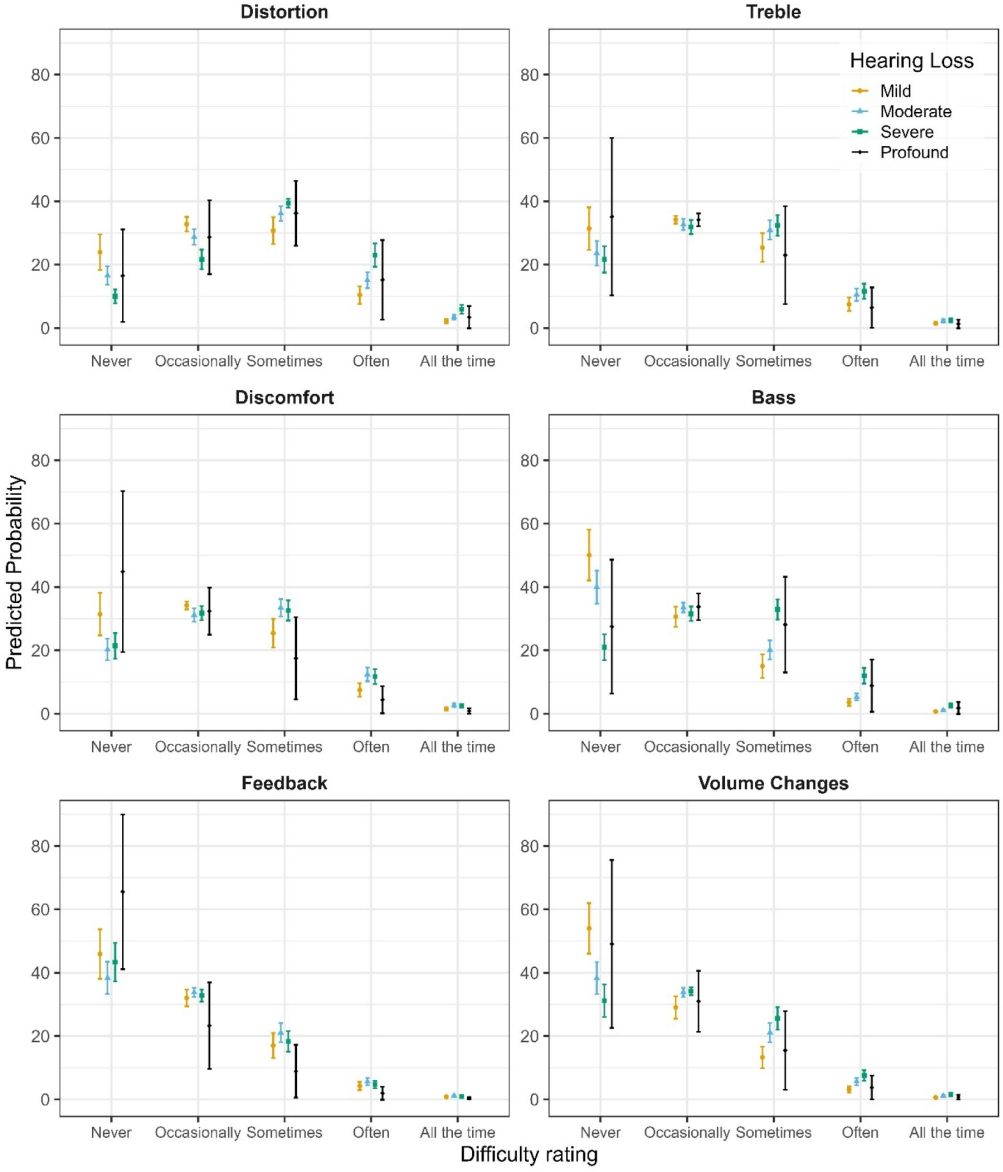
Predicted Probabilities of Experiencing Difficulties for All Items for Each Category of Hearing Loss.

**Figure 6.**
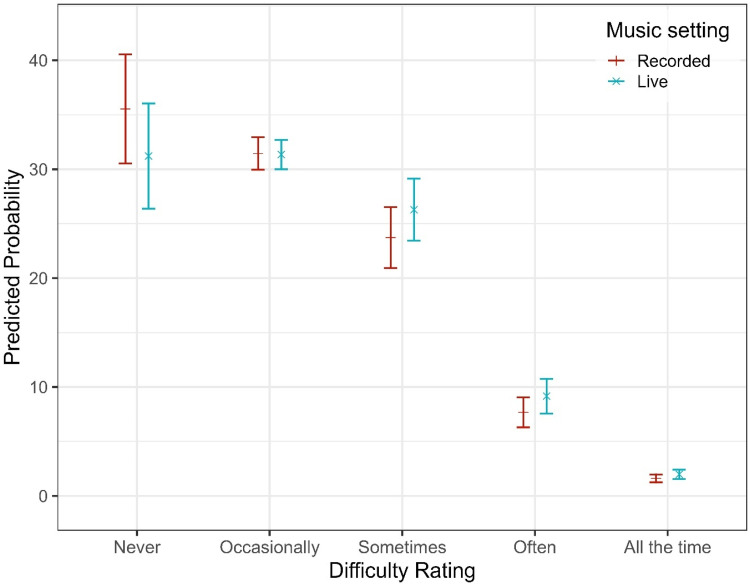
Predicted Probabilities of Experiencing Difficulties for Each Listening Setting.

**Figure 7.**
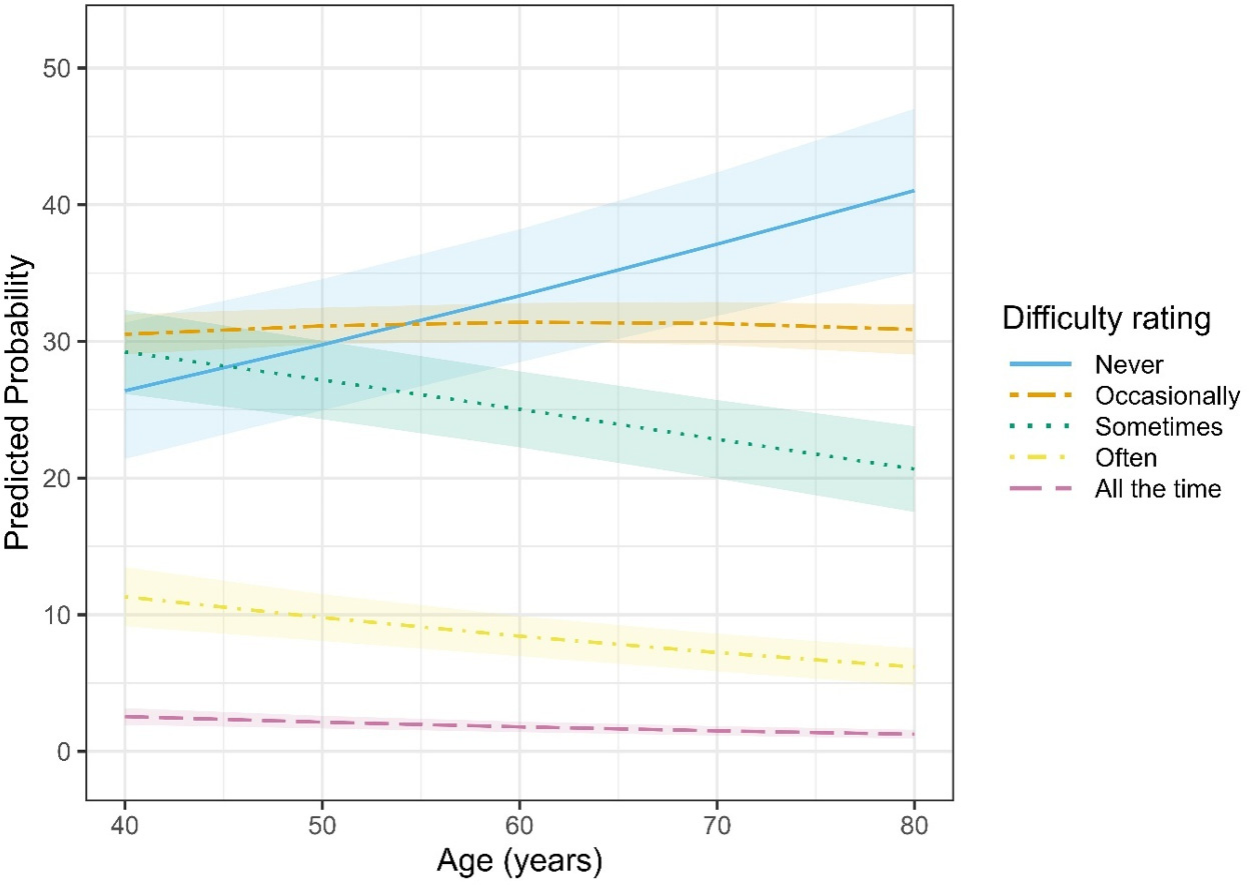
Predicted Probabilities of Experiencing Difficulties as a Function of Age.

Model D4 indicated clear differences in reported difficulties between items (for the complete model output see SM3, Table 2b). After accounting for age, distortion had the highest difficulty rating, reflecting a 165% increase (OR = 2.65, *p* < .001) in odds relative to Bass. Moderate increases in odds were also found for Treble (OR = 1.34, *p = .*045), whereas Discomfort (OR = 1.27, *p = .*085) was not significantly different from Bass. In contrast, lower odds of reporting difficulties were observed for Feedback (OR = 0.54, *p* < .001; 46% lower) and Volume Changes (OR = 0.68, *p* = .007; 32% lower). Despite this, a strong linear increase in reported difficulty with worsening HL severity was observed. Specifically, for the reference item (Bass), each incremental step up the ordinal HL severity scale (mild to moderate to severe to profound) was associated with a 136% increase in the odds of greater difficulty (OR = 2.36, *p = .*012). However, the magnitude of this HL effect varied significantly between items (see Figure 5). For Feedback, Discomfort, and Treble, the linear HL effect was substantially attenuated compared to Bass, being approximately 77% weaker (OR = 0.23, *p* < .001), 72% weaker (OR = 0.28, *p* < .001), and 61% weaker (OR = 0.39, *p = .*011), respectively. In contrast, interactions involving Distortion (OR = 0.66, *p = .*254) and Volume Changes (OR = 0.52, *p = .*078) were not significantly different from Bass. Additional nonlinear (cubic) HL trends indicated subtle curvilinear patterns across difficulty dimensions (OR = 0.67, *p = .*018), suggesting some complexity in how difficulty ratings scale with higher HL severity. Although significant, these effects were considerably smaller than the linear trends. Estimated marginal means (averaged across items and listening settings, with age held constant at the sample mean) further confirmed the overall impact of HL on reported difficulty. Difficulty ratings increased significantly from mild to moderate HL (estimate = −0.47, SE = 0.15, *p = .*011) and from mild to severe HL (estimate = −0.74, SE = 0.16, *p* < .001). No other pairwise comparisons between adjacent severity levels reached significance (*ps* > 0.14). Despite this, across all items and HL severities, live listening settings were consistently rated as more difficult, associated with a 24% increase in the odds compared to the recorded scenario (OR = 1.24, *p* < .001). This indicates a robust main effect independent of item type or HL severity (see Figure 6). Similarly, a significant age effect emerged (OR = 0.98, *p* < .001): Each additional year of age decreased the odds of reporting higher difficulty ratings by approximately 2%, revealing that older participants consistently reported fewer difficulties after accounting for HL, item, and listening setting (see Figure 7). To contextualize this finding, complementary analyses with age treated as standardized predictor were conducted in which HL was coded as a numerical variable. Results indicated that an increase of one HL severity level (e.g., from Mild to Moderate) exerted an effect size comparable to approximately ∼37 years of ageing, underscoring that HL had a substantially stronger impact on difficulty ratings than age.

Predicted Probabilities of Self-Reported Difficulty Ratings for All Items, Estimated from a CLMM of Model D4, Holding Age Constant at the Sample Mean and Averaging Across Listening Settings. Hearing Loss Severity (HL) Groups Are Displayed with Different Colors and Symbols: Mild (Orange Circles), Moderate (Blue Triangles), Severe (Green Squares), and Profound (Black Diamonds). Difficulty Ratings Ranged from *Never* to *All the time*. Error Bars Indicate 95% Confidence Intervals.

Predicted Probabilities of Self-Reported Difficulty Ratings, Estimated From a CLMM of Model D4, Holding Age Constant at the Sample Mean and Averaging Across Hearing Loss Groups and Items. Music Settings Are Displayed With Different Colors and Symbols: Recorded (Red Pluses) and Live (Blue Crosses). Difficulty Ratings Ranged From *Never* to *All the time*. Error Bars Indicate 95% Confidence Intervals.

Predicted Probabilities of Self-Reported Difficulty Ratings as a Function of Age, Estimated From a CLMM of Model D4. Predictions Are Averaged Across Items, Listening Settings, and Hearing Loss Levels. Difficulty Ratings Ranged From *Never* to *All the time*. Shaded Areas Indicate 95% Confidence Intervals.

#### Qualitative Data on Difficulties

The following sections summarize qualitative data themes around difficulties experienced in all settings. Sub-themes included HA-related, environment-related, and music-related difficulties (see Table 7 and SM4). The largest sub-theme was HA technology related difficulties which, reflecting findings for live and recorded music listening, included key themes of Distortion (313), of which difficulties with hearing aids around Dynamics/Loudness (212) were most prevalent. These included music suddenly sounding too loud (sometimes painfully loud, particularly in live contexts), too quiet, or HAs not coping with sudden changes in volume. Difficulties reported often related to HAs making adjustments automatically due to noise-reduction settings or feedback limiters, without the ability to control these, for example, experiencing the limiter suddenly stopping music getting louder, or struggling to find a good volume level even when users had a volume control (see Strategies). Another distortion difficulty was related to timbre perception (164), for example, at higher frequencies and volumes in live contexts. Participants reported music sounding “unnatural” and “artificial” using HAs, experiencing too much treble (113) (described as “tinny,” “harsh,” “shrill” or “scratchy”), and overall poor sound quality (89) including “burbling,” “crackling,” “hissing,” “rattling” and “warbling.” Whether listening or performing, “whistling” feedback sounds were a source of frustration and negatively affected the enjoyment of music.

**Table 7.** Difficulties Reported Listening With Hearing Aids – Summary of Most Numerous Qualitative Themes Coded (Count = Total Number of Times Coded).

Difficulty	Theme	Count	Illustrative quotations
*Hearing aid technology-related*	Distortion	313	*“Too much distortion and picking up of extraneous sound. Hearing aids do not amplify very loud live music in any way that can enhance it and it is easier to not put them in.”*
	Dynamics/loudness/compression	212	*“There are moments where I have to turn the volume up high and moments where the sound is so loud I need to mute the aids. It's a constant battle and struggle and is frustrating. It seriously hinders my enjoyment of music, which is a huge pity as music is my life.”*
	Frequency/pitch perception	164	*“Am still working on getting aids adjusted for high pitch distortion with soprano recorders.”*
	Too much treble	113	*“Trebles can sometimes be harsh and unpleasant”.*
	Sound quality	89	*“H/As certainly allow me to hear more than I would otherwise. But the quality is poor”*
	Feedback	78	*“I find that high pitch soprano, violin solo and woodwinds often give a ‘ringing screech’ type of feedback. This only occasionally happens in live concert performances.”*
*Music-related*	Frequency domain (spectral)	238	*“I have great difficulty in making out the melodic and harmonic detail.”*
	Live contexts too loud	209	*“Live gigs are extremely difficult, I end up taking my hearing aids out or using my remote control to mute them.”*
	Hearing lyrics	161	*“It's all a mush! I only see the benefit of wearing HAs if I could hear lyrics. Wearing the HAs can distort the sound and if there's no added benefit of hearing lyrics I might as well listen without and have a softer, less tinny sound.”*
	Loud dynamics	123	*“My hearing aids get overwhelmed in a loud environment.”*
	Music sounds unnatural/wrong	112	*“Music often sounds “inaccurate”. E.g. music sounds off. Music I am familiar with, sounds unfamiliar.”*
*Environmental-related*	Second amplification	93	*“The reason I choose not to wear HA in some music venues is because the sound amplification is already so loud (often at indie, rock, pop, rap gigs) that the HAs can sometimes make it uncomfortably loud / sound can distort - especially if I end up in a crowd near the speakers. The volume is getting louder in many venues but the clarity is not necessarily better. As much as I love going to gigs (it is a huge part of my life), it can be very tiring listening to live music with (& without) HA, and as a result I've found myself going to less gigs than I would like to.”*
	Source signals	75	*“Person on mixing desk often have music too loud for the size of venue causing distortions and resonance.”*
	Background noise	57	*“The main problem I have when listening to live music is with the crowd. Background noise (particularly on open air stages, primarily at festivals) sometimes makes it very difficult for me to distinguish the actual music from everything going on around me.”*
	Musical stages	46	*“The acoustics in the hall are crucial. I've given up trying to listen to recitals in churches and similar environments, while somewhere like the Royal Festival Hall is excellent.”*
	In the car	35	*“When I am in the car- the noise of the car and the traffic outside affects my listening to the music. I often have to turn volume up, which doesn't necessarily improve the quality of the music.”*
*Reports of self*	Negative mental states	227	*“I recognise very little music now and am unable to sing in tune, so for me it's been like a bereavement.”*
	Reduced pleasure/enjoyment	178	*“My enjoyment of classical music is spoilt by the current hearing aid.”*
*Reports of others*	Audiologist lacked knowledge	53	*“My audiologist appears to have no knowledge of the subject. Music is never tested.”*
	Audiologist unhelpful/uninterested	45	*“My audiologist attempted to change the settings to improve things for me, when I returned to complain and explain she said it was the best it was going to be and there's nothing more that can be done. She was not at all interested that I have a musical background and not understanding of how not being able to enjoy music has deeply affected me.”*

A second key area of difficulties included responses and attributions about music itself. Given the overall high usage of HAs and helpfulness reported, it is likely these responses also relate to experiences while using HAs. The biggest sub-theme was Frequency/ Spectral-related difficulties (238), which included problems with music sounding out of tune, hearing different pitches in the two ears or not being able to hear harmonies or chord progressions. It also included difficulties picking out particular instruments, particularly those with notes in the same range or in complex (often classical) music. Participants reported music sounding like “a wall of sound,” “mush,” or “muddled.” Difficulties with Loudness in live contexts (209) were frequently reported. Difficulties Hearing lyrics (161), particularly with unfamiliar music but also with familiar music, reduced enjoyment and sometimes led to reduced listening overall. Even familiar music was described as “cacophonous”, “unidentifiable” or “unrecognisable,” or at odds with memories of how music used to sound. Similarly, Music sounding unnatural/wrong (112) included descriptions of music as “inaccurate,” “muddy,” or “strange,” often resulting in the removal of HAs for music listening (see Strategies).

A third key area was Environmental-related difficulties. Amplification in addition to that provided by hearing aids was a sizable theme here (93), along with related difficulties around source signals, which included experiences of HAs amplifying music that was already amplified in live contexts, poor mixes, or bad quality sound systems causing distortion and resonance. Some participants reported that these difficulties stopped them from attending live events altogether. Background noise (57), such as noisy crowds and talking, was also found to be a challenge, with some reporting that talking can be louder than the music at live gigs. The noise of traffic while listening to music in the car (35) was also reported as a challenge. Particular venues, locations or acoustics created worse listening experiences than others.

Reports of Self were a large theme centered around positive or negative affect. It included Negative mental states (227) and Reduced pleasure and enjoyment (178). Many of these responses appeared to be a direct result of experiencing poor sound quality for music using HAs, causing annoyance, frustration, tiredness and exhaustion. For some, this extended to sadness, sorrow, anxiety or depression, whose effects were reported to have been experienced over longer time periods. Some music performers reported that their listening difficulties negatively affected their self-esteem and confidence as a musician, or reported experiencing sadness when watching other people enjoy music.

### Strategies Used in Recorded and Live Settings

The use of certain strategies in recorded music listening was mixed; most reported “Never” adjusting volume, changing program or moving away from the sound source to improve listening (SM13). In live music settings, again, the modal response was that participants “Never” adopted one of the three strategies, adjusting volume, changing program and moving away from the sound source, although a larger proportion did “at least occasionally” adopt one of these strategies. Participants were more likely to adjust volume in live than recorded settings (see SM13). Open-ended, qualitative responses reinforced this finding.

Another CLMM was run to examine predictors of strategy use in music listening across three dimensions: adjusting volume, adjusting programs, and adjusting position (i.e., moving away from the sound source). The final sample comprised 884 respondents with 4,963 item-level observations. Likelihood-ratio testing indicated that the inclusion of HL severity and its interaction with item type significantly improved model fit compared to the baseline model (ΔAIC ≈ −25, χ²(9) = 43.20, *p* < .001). Adding listening setting (ΔAIC ≈ −13, χ²(1) = 15.51, *p* < .001), age (ΔAIC ≈ −12, χ²(1) = 14.05, *p* < .001), and musical training (ΔAIC ≈ −31, χ²(1) = 33.12, *p* < .001) each provided significant improvements, whereas gender did not (ΔAIC ≈ + 3, χ²(2) = 0.19, *p = .*91; see SM3 Table 3a for details on model selection). Accordingly, the final model (S6) included item type, HL severity and its interaction with item type, listening setting, age, and musical training. This model explained 40.8% of the total variance in strategy ratings (marginal *R*² = 0.070), with an ICC of 0.36.

**Figure 8.**
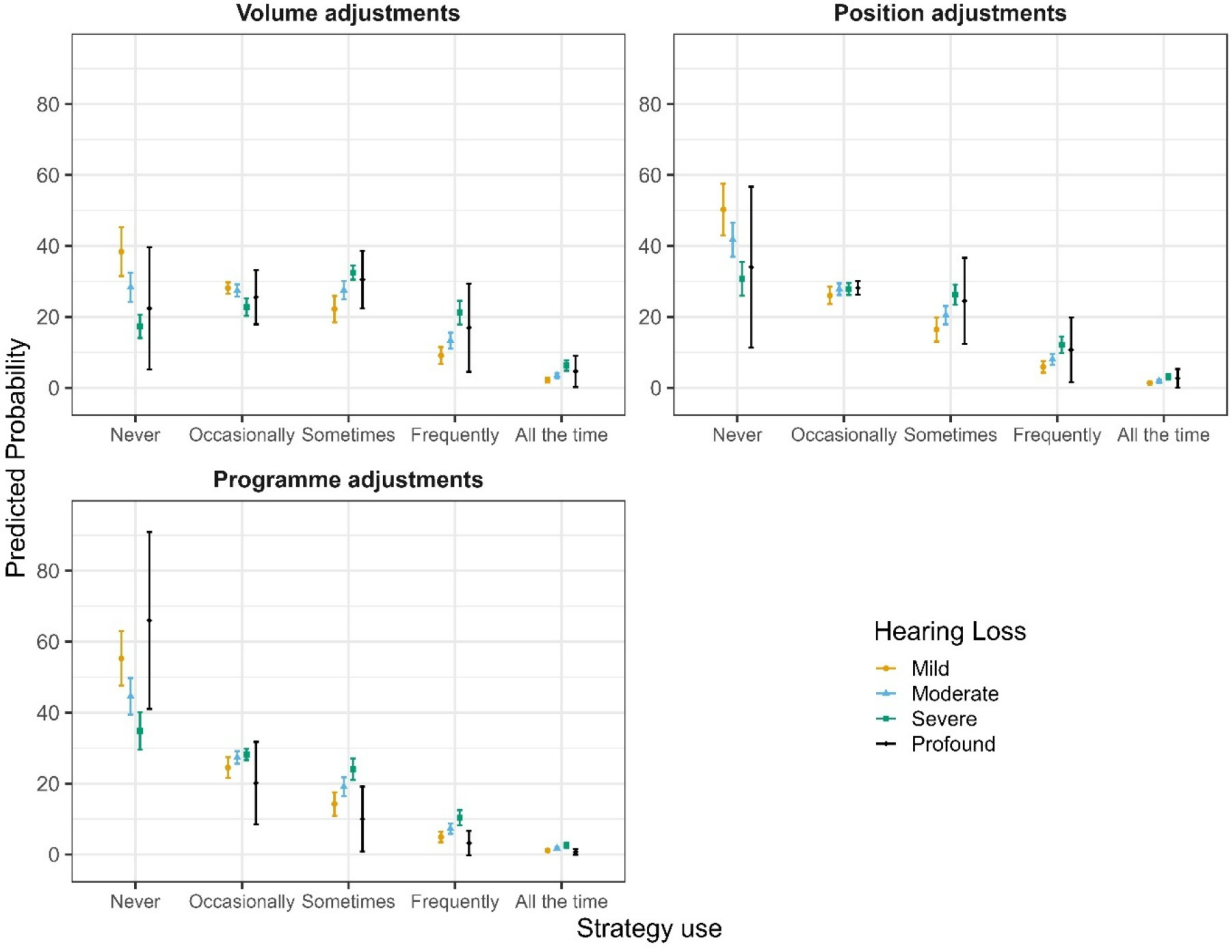
Predicted Probabilities of Strategy Use for All Items for Each Category of Hearing Loss.

**Figure 9.**
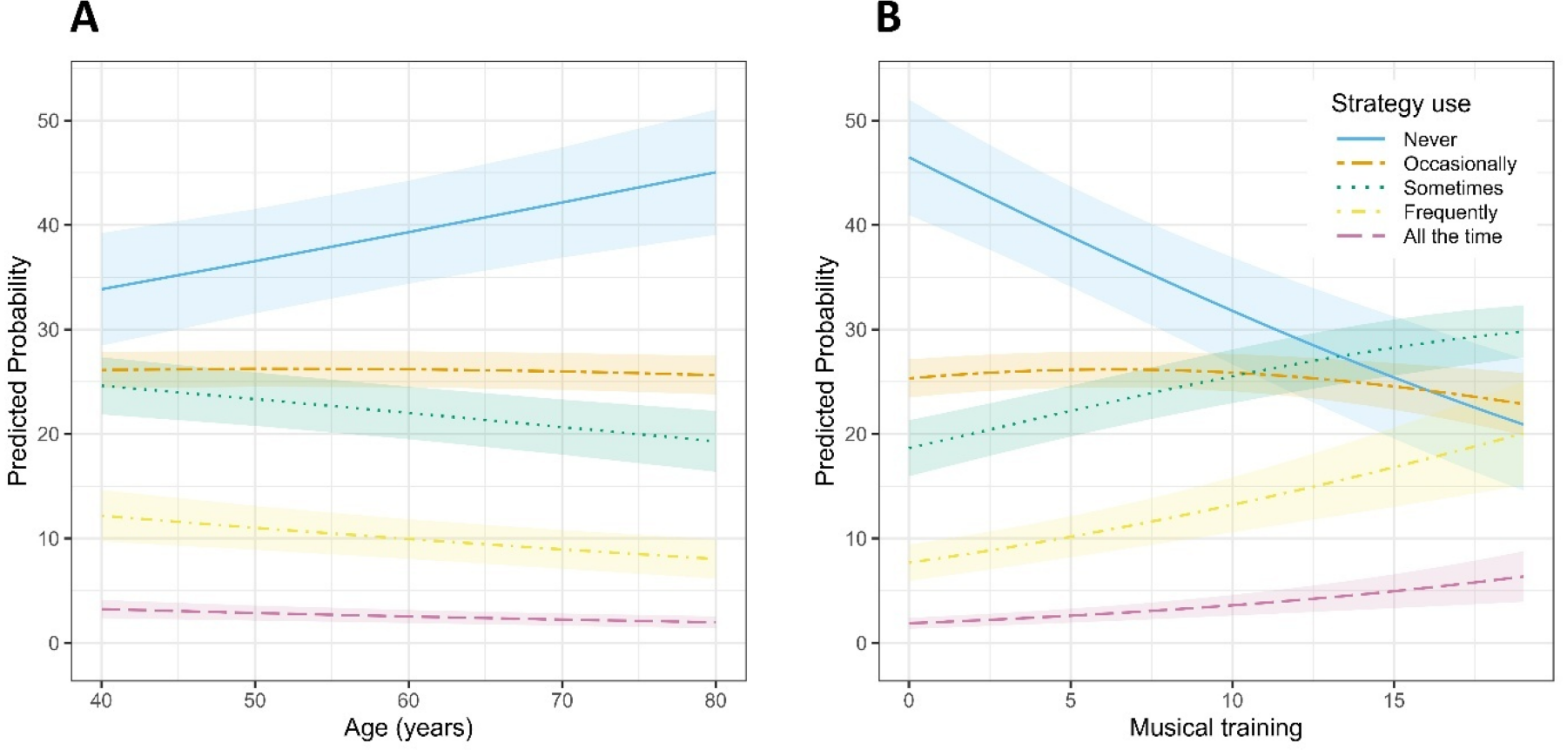
Predicted Probabilities of Strategy Use for All Items as a Function of (A) age and (B) Musical Training.

**Figure 10.**
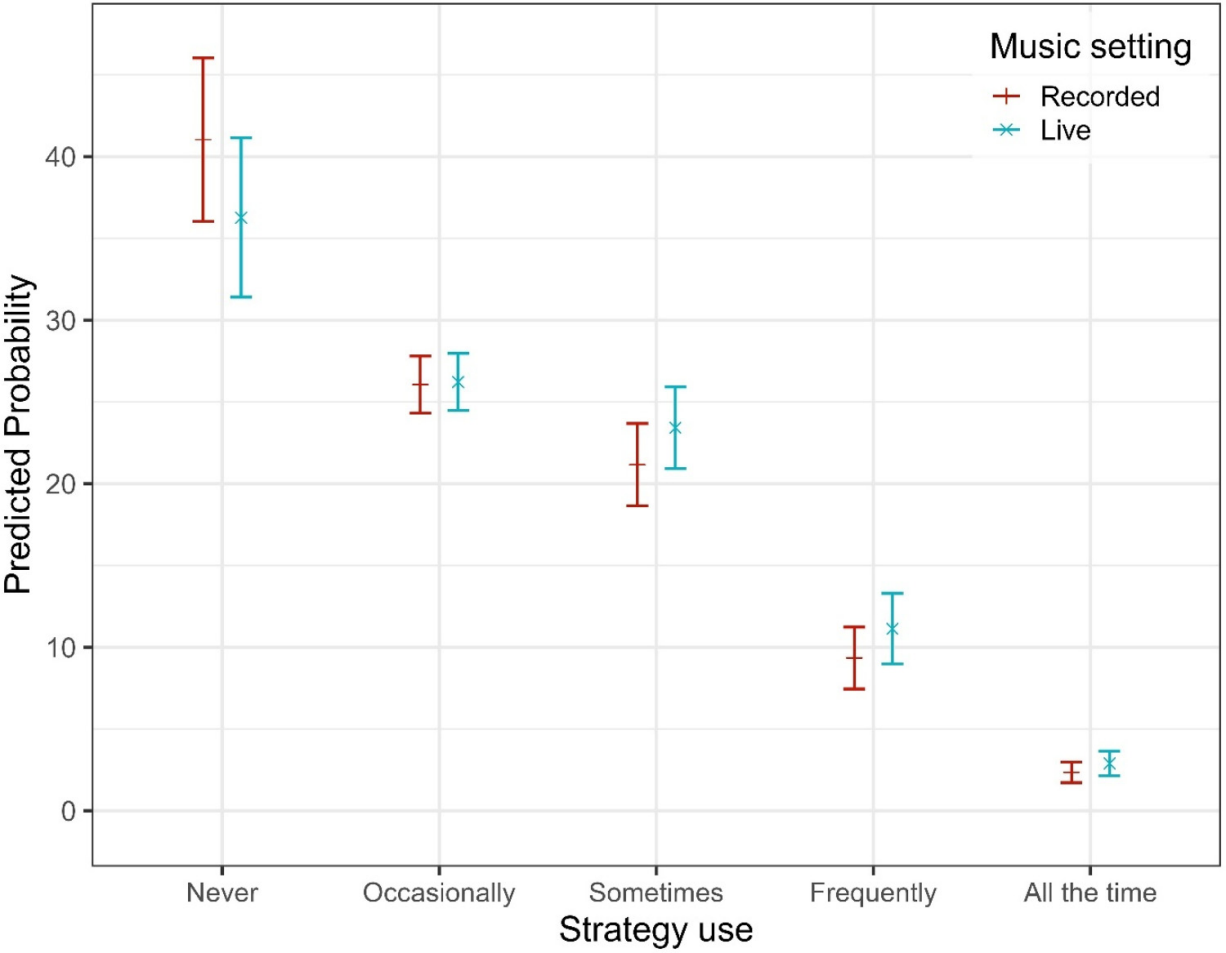
Predicted Probabilities of Strategies Use for Each Music Setting.

Participants reported various strategies to improve music listening. Adjusting the volume on HAs was the most commonly used approach, with significantly higher odds of adopting this strategy compared to moving away from the sound source (OR = 1.83, 95% CI [1.39, 2.41], *p* < .001; see SM3 Table 3b). Changing the HA program (e.g., switching to a music setting) was used less often, with significantly lower odds of endorsement than moving away (OR = 0.63, 95% CI [0.47, 0.86], *p = .*003). Strategy use was further shaped by individual and contextual factors. Pairwise comparisons of estimated marginal means (averaged over items, listening setting, and when both age and musical training level were held constant at sample mean) revealed significant differences in reported strategy use across HL severity groups. Compared to participants with mild HL, those with moderate (estimate = −0.44, SE = 0.15, *z* = −2.96, 95% CI [−0.75, −0.12], *p = .*016) and severe HL (estimate = −0.86, SE = 0.16, *z* = −5.48, 95% CI [−1.17, −0.55], *p* < .001) reported significantly more frequent strategy use. Severe HL was also associated with greater strategy use than moderate HL (estimate = −0.42, SE = 0.13, *z* = −3.28, 95% CI [−0.69, −0.15], *p = .*006). However, the effect of HL varied across strategy types (see Figure 8). Specifically, program adjustment showed a reduced linear HL effect relative to the reference item (adjusting position), as indicated by a lower odds ratio (OR = 0.46, 95% CI [0.21, 1.03], *p = .*058) and a significant quadratic trend (OR = 0.54, 95% CI [0.29, 1.00], *p = .*050). In contrast, the effect of HL on volume adjustment did not differ significantly from the reference, with no significant linear (OR = 1.10, 95% CI [0.54, 2.26], *p = .*786), quadratic (OR = 0.87, 95% CI [0.50, 1.52], *p = .*618), or cubic trends (OR = 0.92, 95% CI [0.68, 1.26], *p = .*605). Age had a consistent negative influence, with each additional year reducing the odds of reporting higher strategy use by about 1% (OR = 0.99, 95% CI [0.98, 0.99], *p* < .001; see Figure 9a). Standardized analyses showed that the impact of a one-level increase in HL severity (e.g., from mild to moderate) was comparable to roughly 27 years of ageing. Musical training further amplified this tendency: each additional point on the training scale [0–19] was linked to a 7% increase in the odds of reporting more frequent strategy use (OR = 1.07, 95% CI [1.05, 1.09], *p* < .001; see Figure 9b). Beyond these factors, listening context also mattered: participants reported more frequent strategy use in live than in recorded settings (OR = 1.24, 95% CI [1.11, 1.39], *p* < .001; see Figure 10).

Predicted Probabilities of Self-Reported Strategy Use for Improving Music Listening, Estimated From a CLMM of Model S6, Holding Age and Degree of Musical Training Constant at the Sample Mean and Averaging Across Listening Settings. Hearing Loss Severity (HL) Groups Are Displayed With Different Colors and Symbols: Mild (Orange Circles), Moderate (Blue Triangles), Severe (Green Squares), and Profound (Black Diamonds). Strategy Use Ratings Ranged from *Never* to *All the time*. Error Bars Indicate 95% Confidence Intervals.

Predicted Probabilities of Self-Reported Strategy Use as a Function of (A) Age and (B) Musical Training, Estimated From the Final CLMM (Model S6). Predictions Are Averaged Across Items, Listening Settings, and Hearing-Loss Severity. in Panel A, Musical Training is Held at the Sample Mean; in Panel B, Age Is Held at the Sample Mean. Strategy Use Ratings Ranged From *Never* to *All the time*. Shaded Areas Indicate 95% Confidence Intervals.

Predicted Probabilities of Self-Reported Strategy Use for Improving Music Listening, Estimated From a CLMM of Model S6, Holding Age and Degree of Musical Training Constant at the Sample Mean and Averaging Across Hearing Loss Groups and Items. Music Settings Are Displayed With Different Colors and Symbols: Recorded (red Pluses) and Live (Blue Crosses). Strategy Use Ratings Ranged From *Never* to *All the time*. Error Bars Indicate 95% Confidence Intervals.

#### Qualitative Data on Strategies

While a large number of participant responses related to difficulties, a similar number of responses were coded to general “strategies” involving active and positive behavior by HA users to support their music listening (see Table 8 and SM4). The largest number of these was technology-related, including experimenting with and adjusting HA settings, programs, and other related technologies.

**Table 8.** Strategies Adopted When Listening to Music with Hearing Aids – Summary of Most Numerous Qualitative Themes Coded (Count = Total Number of Times Coded). Strategies may be Mixed in Valence Either Positive, Neutral or Negative.

Strategy	Theme	Count	Illustrative quotations
*Technology-related*	Remove hearing aids	453	*“I enjoy live music more without my aids because of the distortion and sudden high volume. When I play the piano myself I take my aids out or turn them off.”*
	Hearing aid use or model selection	332	*“When I got my current aids, I remember enjoying the broader spectrum it provided - like wearing a pair of glasses that let you see a more subtle range of colours.”*
	Music program on	233	*“In the normal speech mode the missing upper frequencies such as sibilants, plosives etc. are ‘re-directed’ or ‘folded back’ into my hearing range as a faux-hiss or faux-click. This somewhat restores the impression of ‘normal’ speech. This mode plays havoc with music and renders it difficult to listen to, making it harsh, ‘hissy’ and generally unpleasant. However the music and singing settings, while not able to replace the missing frequencies, do go some way to helping with distinguishing flute, clarinet and oboe in their upper registers.”*
	Use headphones	126	*“I listen casually to recorded music with my hearing aids when doing the dishes, say. But for serious listening, I take them out and use Sennheiser 650 headphones or similar good pro or consumer headphone technology.”*
	Use ALD	119	*“I use wireless connection to my hearing aid a lot. The wireless system distorts the sounds, limits the bandwidth and in general makes the sounds kind of brittle or harsh. Still it is useful and often provides enough clarity that I can learn a new song without disturbing others in my home.”*
	Use volume control	111	*“About 10 years ago hearing aids did not have volume controls (perhaps early digital aids - maybe so called automatic volume levelling) - I believe it was a “fad” introduced by well-meaning technicians/hearing aid designers, but in reality was a real problem: no ability to respond to low volumes - quiet orchestral sections for example. The wider the range of volume control the better as far as I'm concerned.”*
	Use hearing protection	96	*“The music is too loud at live events for me to comfortably wear my aids. I protect my ears with plugs more often than not.”*
	Hearing aid adjustments	93	*“Audiologist made some changes to program settings that improved the music listening experience.”*
*Music-related*	Avoid loud music	171	*“Music is distorted so I usually avoid live music. Even if it is not cacophonous I no longer ever hear the intricacies of harmony that I used to enjoy. I would only go to an event with live music if there were some other reason for attending.”*
	Selecting venues/position	97	“*When listening to orchestral music or musical theatre I have to sit with my hearing away from the strings or they are too loud and the sound is distorted, I now always sit on the left of the auditorium.”*
	Select familiar music	85	*“I have a difficult time hearing and learning new “pop” music from recordings. I rely on listening to music that I know, because my brain fills in the gaps of what I remember it should sound like.”*
	Select instrumentation/ensemble	76	*“I am profoundly deaf in higher and middle frequencies. I played flute to Grade 8, double bass to Grade 5 and violin to Grade 7. I had to give up the violin when my hearing got worse. I moved to the viola but then it got worse again so I took up the double bass.”*
	Select music features/genre/repertoire	73	*“I have changed to listening to more classical or instrumental music as I don't find it so frustrating and the instruments are clearer. Either that or heavy rock/dubstep/drum and bass - I can hear (or feel) that!”*
	Multi-Modal Cues	47	“*Ballet is when I hear most music. This has visible rhythm, and “clues” to the musical content. It takes me only 5 or so minutes to “tune in” to the music, then I am on “the right wave length” for the rest of the performance. I can use one sense to back-up another.”*
*Strategy did not fix problem*	Strategy did not fix problem	279	*“They gave me a music program but I tried it and didn't like it, and then never used it again!”*
*Reports of self*	Perseverance	156	*“Music has been part of the discussion with my consultant at the hospital since I first lost my hearing 9 years ago. It is always on the agenda but I do not expect her to be able to ‘fix’ it for me. It is my ongoing challenge to fix it for myself.”*
	Passion for music	103	*“I really miss music. I used to play in bands and enjoy listening to a wide range of music. It is not really a pleasant pastime any more. I'm filling up writing this. It's the saddest part of being deaf for me. It's taken away one of my real pleasures in life.”*
*Reports of others*			
	Audiologist helpful or interested	69	*“My audiologist recognized that music was important to me and recommended adding the music programme which has made all the difference to my life experience.”*

A large number of responses referred to HA or model selection (322), such as trying out HAs in different situations or trying newer or older models. A very small sub-set reported preferring their older analogue HAs for music listening. A key strategy was use of the Music program (233). For some, this was beneficial, helping them to distinguish instruments more clearly and easily, especially where additional adjustments had been made by audiologists to the gain, compression and microphone directionality. Others, however, reported that the music program made no difference or occasionally made things worse. In settings where participants listened to recorded music at home or on public transport, many reported removing their HAs to Use headphones (126) instead. This was reported to improve clarity, sound quality, and tonal balance. Some reported use and exploration of Assistive listening devices (119) including T-loop, inductive ear hook, Bluetooth, remote microphones, and phone apps, the effects of which ranged from “beneficial” and “revolutionary” to inconvenient. Bluetooth streaming to HAs and phone apps which allowed the HA user to control volume and frequency balance were mentioned most often as having a positive impact, improving the experience of music and in some cases increasing engagement in music listening. Loop systems were found to be helpful in live settings but were not always available or working well. Other reported issues were switching between music and speech, and a lack of technology compatibility (e.g., proprietary equipment), as well as distortion and poor sound quality. There were some reports of accessibility issues, such as lack of sign language interpretation of performance, especially by those with profound HL. Benefits of the use of Volume control (111) were mixed. Many appreciated the ability to adjust the momentary volume, particularly with a phone app, but many also struggled to achieve the “right” volume, especially in live settings, and ended up adjusting constantly. Many loved having a mute button for the two HAs independently, and in general, greater control was well received. The Use of hearing protection (96) as a strategy was often connected with reported knowledge about the dangers of loud music, protecting against further damage and, occasionally, regret at not having worn it earlier in life.

Finally, a very large proportion of comments referred to Removing HAs altogether for music listening (453). This strategy was more frequently reported by those with mild or moderate HL (reflecting the quantitative result) and was often reported for live or amplified music settings, depending on the genre. For many, switching HAs for the greater attenuation provided by earplugs resulted in more pleasant or natural sounding music, even if details were missing. Cross-tabulation analysis of coded frequencies (see Table 9) revealed that the difficulties most strongly associated with removing HAs were difficulties with live or loud music (108) and distortion (62). HA removal was also associated with strategies, most frequently: using earplugs or hearing protection (63) and using headphones (55).

**Table 9.** Associations for Removing Hearing Aids When Listening to Music (Theme Count = 453).

Theme	Sub-theme	Cross-tabulated theme count
Difficulties	Difficulties with distortion	62
	Difficulties with frequency range or pitch	14
	Difficulties with dynamics, loudness	54
	Difficulties with live or loud	108
	Difficulties with dynamics – loud	48
	Difficulties with sound quality	31
	Difficulties with feedback	13
	Difficulties with phantom sounds	10
Strategies	Avoiding live music	10
	Using headphones	55
	Using earplugs or hearing protection	63
	Avoiding loud music	12

A second main group of strategies was coded around Music-related strategies, the most common of which was Avoid loud music (171). Participants reported that loud music sounded like “noise”, “cacophony”, or “distorted”, with live settings in particular linked to reports of tiredness, headaches, hyperacusis, or fear of hearing damage. Participants avoided settings where this occurred and listened in more controllable situations. For those who did attend loud music events, there were reports of selecting venues/positions (97), to exert control over where they sat in relation to performers or the sound source (e.g., front or back, left or right). Some were reconciled with attending such events only to accompany others or support friends and family, not for their own enjoyment. This was related to reports of selecting to attend or listen only to certain instrumentation/ensemble (76) combinations and Genres (73), where this would result in a better listening experience. For example, some with high-frequency losses would avoid higher-register instruments such as violins or flutes, preferring lower-register cellos and basses instead. A few reported preferring less complex music as their HL increased over time. Selecting familiar music (85) was reported by many as a way to mitigate difficulties in perceiving music, since memories of familiar music helped “fill the gaps.” This was related to reports that it could be difficult to engage with new songs/pieces or hear new lyrics, for example, requiring many repeated listens to gain familiarity before going to see a new artist or performer. Some reported they would seek out multi-modal cues (47), for example, reading lyrics or live captions while listening, or watching the bow of a string player or the lip-pattern of a singer, and this was reported to add to the enjoyment of the musical experience.

The active decision-making and involvement of participants with music-listening situations was reflected in many responses, especially in a theme coded Perseverance (156) under Reports of Self. Many reported the need to “keep trying” with music, in spite of the difficulties experienced. This was linked to sentiment around Passion for music (103), which likely supported regular listening and engagement behavior over time. Perseverance also included taking time to acclimatize to HAs and engage in listening practice to help perceive musical elements, making repeated trips to the clinic to adjust existing HAs or trial new devices. However, 279 responses were coded as Strategy did not fix problem, further highlighting the mixed success of all strategies, whether initiated by the user or by an audiologist.

#### Audiologist Involvement

Participants were asked when they last visited their audiologist, whether they had ever discussed music with the audiologist, and, if yes, who had brought up the topic. They were also asked whether the discussion improved their experiences, and were encouraged to report more in an open-ended response. Of 1,300 respondents, 63% had visited their audiologist within the last six months, and 37% six months ago or more. There was no significant effect of HL level on how often or when an audiologist had been visited (Table 3 §28–30). Over half (58%) had discussed music while 42% had not. Those with moderate and severe HL were more likely to have discussed music than those with mild HL (χ^2^(3) = 14.93, *p = .*002, *V* = 0.11). In discussions about music, the vast majority (88%) reported that they brought up the topic rather than the audiologist (8%) (4% could not remember).

**Figure 11.**
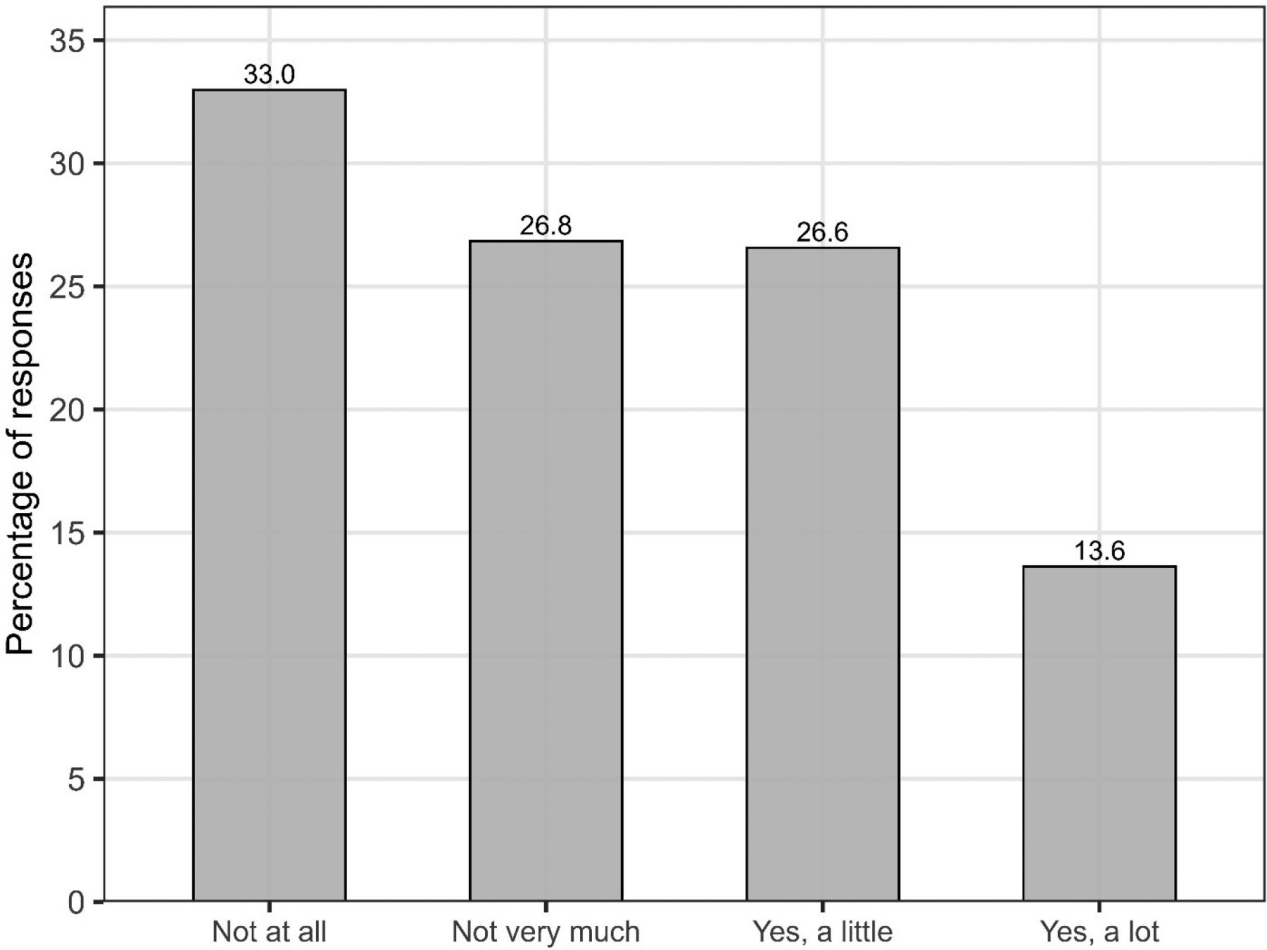
How Often Discussing Music With an Audiologist Improved Music Listening Experiences (*N =* 749).

There were mixed responses about whether discussions with audiologists had improved music listening experiences: 33% reported “Not at all”; 54% “Not very much” or “Yes, a little” while only 14% reported “Yes, a lot” (Figure 11). There was no association with HL level (χ^2^(9) = 9.61, *p = .*383, *V* = 0.06). Qualitative responses reflected this mixed picture, with 69 responses coded as examples of “Audiologist helpful or interested in Music-related strategies” (see Table 8). This helpfulness came in the form of adjustments to HA settings, disabling automatic functions (feedback or noise reduction), or other advice, which sometimes led to improved outcomes, but sometimes not. A small number proactively sought help from audiologists with expertise in music. Negative affect states were reported relating to perceptions that their Audiologist lacked knowledge (53), or was Unhelpful/uninterested (45), and perhaps too time-pressured to be able to resolve music-related issues in the clinic (see Table 8). Some were frustrated that they were not given hearing tests using music stimuli to help adjust their HAs. Others were told that there was nothing more to be done and that they should accept the limits of the technology with respect to music.

## Discussion

Irrespective of HL, the participants in the survey engaged with music for pleasure, relaxation, health and wellbeing, consistent with prior studies on everyday musical engagement among the general population (Groarke & Hogan, 2016; IFPI, 2023; Lamont et al., 2016; Schafer et al., 2013). A reassuring aspect of the present study was the high level of musical engagement found in the sample, including 6% of respondents representing the BSL community, often under-represented in academic research. In spite of the many difficulties reported about music listening with HL and HAs, 87% agreed that music was very important to them, 84% still listened to recorded music and 66% attended live performances. The measures of engagement reported here are comparable with statistics in previous research and industry reports. For example, Krause et al. (2013) found that 83% of adults agreed or strongly agreed that music was important, and a recent industry report showed that adults on average attend four live events/annum (WiFi Talents, 2025). These data support the idea that music listening can and should form part of clinical discussions in audiology, given its known cognitive, emotional, and social benefits (Cohen et al., 2006) and connections to general health and well-being (MacDonald et al., 2012), especially for older adults (Viola et al., 2023).

While engagement with music was high, the helpfulness of HAs was mixed. While modal scores for both live and recorded settings were high (good) (8/10), the distribution of scores had a large negative skew (mean scores 6/10 for both), reflecting some unhelpful aspects and difficulties using HAs for music listening. Across all participants, HAs were reported as less helpful for hearing lyrics and picking out instruments than for hearing the bass line, melody, and singer. There was a main effect of HL, such that those with higher HL severity reported lower levels of helpfulness of HAs across listening dimensions, with significant linear trends for hearing lyrics and picking out instruments. This is likely due to greater challenges in source separation among those with higher HL severity (Hake et al., 2023; Moore, 2016). Non-linear trends also emerged for hearing lyrics and the singer, with helpfulness ratings tending to plateau for these listening dimensions. This is likely due to the characteristics of the Profound HL group who showed consistently greater variation in responses to items than the other groups. This group is highly dependent on their HAs, so may be more likely to report HAs as helpful, irrespective of different listening dimensions. There was also a main effect of the listening setting. The live music setting was associated with a 31% reduction in the odds of reporting higher helpfulness ratings compared to recorded settings, and the overall helpfulness rating was slightly but significantly lower in live music settings (average 6.5/10 vs. 6.8/10). Given that HAs are primarily designed to improve the intelligibility of speech, difficulties are likely to occur when the input signal has a very high sound level and or large frequency range, as occurs in live settings. This is especially the case for those with more severe HL. Challenges with wide dynamic range and high peak intensities in live settings have been identified in prior research (Chasin & Hockley, 2014) and may help explain the experiences described by HA users in live or loud music settings.

Difficulties were reported more often for Distortion and Too much treble than for Feedback and Sudden changes in loudness. This provides support for prior research which has shown that 53% of HA users experience distortion at least sometimes (Madsen & Moore, 2014) and that distortion is a recurring issue (Greasley et al., 2020; Swann et al., 2023). Previous work has also shown that HAs can make music sound “too sharp/shrill” (Marchand, 2019) and “too bright/shrill” (Madsen & Moore, 2014), so it was perhaps unsurprising that participants often reported Too much treble. There was a strong linear trend with HL; those with higher HL severity were more likely to experience difficulties frequently, with significant differences between the mild and moderate, and mild and severe groups. Previous research has suggested a link between higher HL severity and reduced music enjoyment (e.g., Chern et al., 2022; Looi et al., 2019), music sounding distorted (Madsen & Moore, 2014) and having worse tone quality (Looi et al., 2019; Madsen & Moore, 2014). However, unlike the present work, prior work has not identified experiencing Too much bass as a specific issue for those with severe and profound HL. One explanation for this could be that those with more severe HL are likely to have closed ear molds which allow high levels of HA gain at low frequencies, which can introduce vibrotactile sensations that can be unpleasant, even for experienced HA users. Another explanation could be the increased loudness recruitment among these HL groups, such that bass sounds appear disproportionately loud once the hearing threshold is exceeded. These issues with tonal balance suggest that a starting point for the development of new algorithms could be collecting user preference data for equalization strategies for different musical genres. This would also capture some key differences needed for acoustic versus amplified music.

A less intuitive finding, given the positive correlation between age and HL severity (Akeroyd & Munro, 2024), was that older participants tended to report fewer difficulties than younger participants. This could be due to older participants wearing HAs for longer, acclimatizing to them and experiencing fewer difficulties as a result. Additionally, lower expectations of the technology may have affected reporting, such that fewer gave responses for the higher categories (“All the time”).

Distortion was the most commonly reported problem in the study, for both live and recorded settings. Given that distortion can cover a range of perceptual effects, it may be that more specific descriptors (e.g., sudden changes in loudness, fuzziness, noisiness, extraneous sounds) would have enabled participants to better differentiate between difficulties experienced across the two settings. Distortion effects and artefacts for music can arise due to the signal processing used in HAs (Chasin & Russo, 2004) and sometimes due to effects occurring within the auditory system of the individual. An example of the former is that sudden changes in loudness may occur if the automatic gain control (AGC) does not react sufficiently quickly to sudden changes in input sound level. Conversely, if the threshold for peak limiting is set too low, a crescendo in the music may not lead to a corresponding increase in loudness, and may be associated with nonlinear distortion (Moore, 2024). Generally, research has shown that slow-acting AGC is preferred over fast-acting AGC for listening to music (Croghan et al., 2014; Hake et al., 2025; Madsen et al., 2015; Uys et al., 2012) but the results are mixed and were mostly obtained using music covering a relatively small range of input sound levels. The results may not apply to the large range of sound levels, including very high levels, encountered at live music events.

The most common single strategy emerging from qualitative data for reducing the experience of distortion in live or loud settings was removing HAs altogether. This behavior has been documented in prior research (Chasin, 2012; Fulford et al., 2015) and elicited more qualitative comment here than any other strategy (453 responses). This is especially true for those with mild or moderate HL levels. In contrast, other removal behavior could be described as proactive (rather than reactive), including the use of headphones, earplugs or hearing protection.

Within our quantitative data, adjusting the volume of HAs was the most frequently used strategy, although volume control features were judged as a mixed blessing, providing immediate control, but requiring constant adjustment. In contrast, participants were least likely to report changing their HA program. This may be because they do not perceive an added benefit of switching programs (borne out in some of our qualitative data). It may also be the case that participants were less likely to have multiple programs on their HAs at the time when these data were collected. Research based on large-scale real-world data showed that 58% of users had HAs pre-programmed with situational listening programs (e.g., speech in noise, Music, Comfort) (Pasta et al., 2022). Given the increasing likelihood that participants will be wearing HAs with Bluetooth connectivity and using phone apps which facilitate easy switching between programs, changing program as a behavioral strategy may be significantly more prevalent now than was the case when the survey was conducted.

Only 34% of the sample reported having a music program and, of these, less than half used it frequently, in line with prior research showing low to moderate adoption, low reliance and mixed sentiment about the effectiveness of such programs (Looi et al., 2019; Madsen & Moore, 2014; Vaisberg et al., 2019). This highlights the need for further research and development. Qualitative evidence suggests that successful use of a music program requires extra support from an audiologist to make adjustments, e.g., by removing adaptive functions, which highlights the need for appropriate counselling and fitting for music listening, and enhanced guidance to support clinical practice.

The CLMM results showed that strategy use was further shaped by individual and contextual factors. Those in the moderate and severe HL groups were more likely to adopt strategies than those with mild HL. Since those with mild HL are less likely to report difficulties, they may not feel the need for extra steps such as moving away from the sound source or changing program, whereas those experiencing more difficulties are more motivated to try to improve their experiences. Age exerted a negative influence, as older participants were less likely to report using strategies. This may reflect the finding that older participants were less likely to report experiencing difficulties, or of them having worn HAs for longer and having acclimatized to HA performance in different music settings. Musical training was found to increase the likelihood of strategy use; those who were very highly musically trained were significantly more likely to implement strategies frequently. It is likely that musicians are more proactive due to higher demands on listening and sound quality in rehearsal and performance, and are therefore more aware of strategies that work. Finally, there was a significant effect of listening context; participants reported more frequent strategy use in live than in recorded music settings, which is likely due to the finding that participants experience more difficulties (especially distortion) in live settings.

The removal of HAs in live settings highlights a tension between amplification, attenuation and technology use. It is likely that removing HAs altogether provides a simple solution to excessive loudness and exposure to potentially damaging sound levels, but the simultaneous need for protection and amplification suggests that future technology should attempt to combine these features in one device, removing the need to swap devices. In principle, HAs can attenuate high-level sounds. However, effective attenuation is only achieved if a closed fitting is used (as opposed to an open fitting, where the earpiece has one or more openings or vents). Finally, the use of assistive listening devices (ALDs), hearing protection and connectivity settings add complexity to technology use for music listening.

The use of HA technologies exists in a wider context of overall music listening preferences and engagement. Here, classical music was the most preferred genre, reflecting research exploring age-related changes in musical preferences (Bonneville-Roussy et al., 2013). However, those with severe or profound HL had lower preferences for complex musical styles, e.g., classical, orchestral, chamber or choral music, and were less likely to use music for pleasure or enjoyment, supporting prior research linking increased HL with increased dissatisfaction and reduced engagement (Looi et al., 2019; Madsen & Moore, 2014). Perceptual factors such as reduced cochlear frequency selectivity may negatively affect the ability to recognize musical instruments and to perceptually separate instruments or groups of instruments in complex music (Hake et al., 2023; Moore, 2016; Siedenburg et al., 2020). This may affect music listening behavior independently of the adoption of hearing technologies. For example, many participants reported avoiding new music, preferring to listen to familiar music, using their memory of the music to help “fill in the gaps.” While older adults listen to familiar music more than younger adults (Davies et al., 2022), this may be more important for those with HL. Others reported shifting their preference towards less complex styles and choosing pieces that fitted well with residual hearing levels and dynamic ranges. The intelligibility of lyrics is known to be crucial for many in their music appreciation (Greasley et al., 2013), and the reliance on visual cues from performers and captions to augment listening experiences was also reported by participants in this study.

The psychological factors affecting music listening found in this study, such as memory and preferences for musical genre, underline the crucial role of clinical advice in supporting music listening and engagement using HAs, challenging any assumption that HL prevents music engagement (Holmes, 2017). The present study offers large-scale data to underpin such advice. For example, high levels of musical engagement can be assumed for those with mild or moderate HL, and clinicians may advise that removing HAs for music listening may increase enjoyment (Chasin, 2022), especially for live performances. For those with severe or profound HL, for whom reliance on HAs is greater, clinicians may advise on the use of the volume control, music programs and ALDs to maximize musical engagement and enjoyment. The tension between lower perceived helpfulness of HAs for music (relative to speech) but greater reliance on HA technology should be addressed with discussion around expectation management, and the need for persistence, adaptability, and patience when it comes to the enjoyment of music. For example, highlighting situational aspects (live vs recorded listening) rather than HL level may help listeners adopt strategies more positively and proactively. Promoting awareness of the functionalities of technology such as Bluetooth, alternative programs, or even ALDs will likely increase adoption and musical engagement.

## Strengths and Limitations

This study is the largest known to date assessing HA use and music listening behaviors among people with HL who use HAs and bone-conduction HAs. It has sought to disentangle the effects of HL level, listening setting and individual differences variables (e.g., age, gender, musical training) on music experiences. While prior research has highlighted the effect of HL level on musical outcomes (Chern et al., 2022; Looi et al., 2019; Madsen & Moore, 2014), and has shown that HAs are less helpful in live music contexts (Madsen & Moore, 2014), the present study highlights the importance of considering the HA user's age and musical background alongside their HL level and preferred music settings. This study is also the first to systematically investigate the strategies people use to improve their listening experiences, most notably, removing HAs altogether.

### Sampling

While offering the survey in BSL increased accessibility, the number of participants with profound hearing impairment was relatively small (*n =* 35). However, an almost identical equal gender split was achieved, creating a more accurate representation of the population (typically women tend to participate more in research studies than men). The sample mean age was 60 years, roughly similar to prior survey research with HL populations (Looi et al., 2019, mean age = 67 years; Madsen & Moore, 2014, most prevalent age bracket was 60‒70 years), supporting comparisons with these studies. However, the sample was more highly educated than the UK average population (63% vs. 40%, see Statista, 2024) and future research should attempt to achieve a more representative distribution of educational levels. Assessment of differences according to HL level was made on the basis of participants’ self-report of their hearing difficulties. This measure most accurately aligned with the pure-tone audiometric data (*n =* 131 with audiograms) and facilitated analyses based on speech descriptors, which focus on experience/functional hearing. However, even this measure only agreed with the corresponding audiogram half the time (i.e., there was only a 50% match between the HL speech descriptor and the PTA). Nonetheless, the patterns in the dataset are based on large numbers and we feel confident they can be considered indicative of the problems experienced among the different HL groups. Another limitation was that participants were not able to report their estimated degree of HL for each ear separately, so it was not possible to assess the effects of asymmetrical HL. Future research allowing for this would enable exploration of challenges and strategies with music listening for those with highly asymmetrical HL.

There were limitations relating to live versus recorded settings. While certain examples were given (e.g., listening in a car or listening via an MP3 player), other possible situations were not accounted for, such as listening to recorded music in a live venue. It was also not possible to elicit reliable data about the impact of room acoustics on listening experiences. These factors may have contributed to broadly similar difficulties being reported for live and recorded music settings. Data about the degree to which individuals with a music program used it in recorded versus live settings may also have been influenced by ambiguity about the exact listening situation. Finally, categorical responses reflecting frequency of occurrence (“not at all,” “occasionally,” “sometimes,” “often,” and “all the time”) may not evenly reflect the underlying distribution of frequencies, with the result that responses were often rolled up into the category “at least occasionally.” Future research should more precisely indicate frequency of occurrence (e.g., “approximately 1 in 4 times”).

### Age of Data

Survey data were collected between 2016 and 2018, and so the present findings may not reflect advances in technology that have happened since then. For example, Bluetooth functionalities are now standard, facilitating wider use of streaming, and the integration of Artificial Intelligence/Machine Learning (AI/ML) is a new frontier. While the pace of technology change is increasing, however, such changes have been largely focused on improving the intelligibility of speech in noise, rather than on music listening. The supply chain framework for England has not added any additional manufacturers, reflecting the mainstream use of Oticon and Phonak aids observed in this study. It also remains typical for HA users to get new HAs after about five years of use in high-income countries (Bisgaard et al., 2022). It has also been reported that 76% of audiology practices experienced reduced attendance during the pandemic (2020–2022, Manchaiah et al., 2021), which may have reduced the degree to which HAs were replaced during this time.

## Future Research

There is a step-change in the rapid pace of technology development resulting from AI and other emerging forms of technology. Many HA manufacturers are incorporating AI/ML in their devices, and the range and capability of listening devices on the market is evolving rapidly. Portable Bluetooth loudspeakers and wireless headphones and earphones allow greater mobility for music listening in the home or outdoors, and large numbers of people spend long portions of the day using in-ear headphones. Noise-cancelling and hear-through features in these technologies allow listeners to control how connected they remain with the auditory world, and whether or not to reduce the audibility of the sounds of voices, home appliances, traffic or music in order to hear their chosen auditory input. While amplification technology is increasingly being incorporated in personal listening devices, music listening should remain part of HA development, especially given the ubiquity of music listening and engagement across all hearing levels.

Trends in Eurotrak data (between 2012 and 2025) show that for those countries that rate music listening as important, satisfaction with HAs in noisy settings has increased by about 17%, but satisfaction with HAs for music listening has only increased by about 6%. The issue of distortion when listening to high-level music must remain a priority for research investigations, especially given that such distortion often results in users removing their HAs altogether when they would otherwise choose to wear them. A typical paradigm for digital signal processing research, however, is to conduct controlled experiments using recorded stimuli covering a limited range of levels.

Future work on music and HA usage should ask participants to provide the make and model of HAs and more detailed questions about music program use and settings. Researchers should aim to recruit participants who use a wider range of types of HA, such as RTE, ITC, CIC, and BCHDs, to enable comparison between HA types (over 90% in the current sample were BTE) and dome type (open/closed). Research should include evaluations of signal processing methods in live music or concert settings, with larger samples of HA user groups, perhaps utilizing experience-sampling methodology to support ecologically valid data collection at scale. It should also broaden scope to include a range of ALDs and Bluetooth use cases in musical settings. Bluetooth streaming is now standard in many HAs but there is limited data on uptake and use. Some studies have reported poor music quality via HA streaming (e.g., Bennett et al., 2021; Desai et al., 2024; Murdin et al., 2022) and this requires further research. Data logging from HAs is also a viable research tool to capture listening environments, duration of listening, user preferences for programs and volume settings.

Emotional responses to music depend on a range of acoustic cues, such as fluctuations in fundamental frequency and level, harmony, tempo and rhythm (Juslin & Timmers, 2010; Moore, 2024). More research is needed to show how HAs may support perception of these elements and in turn increase positive emotional experiences and HA adoption. Future research may also benefit from including sound quality measures developed specifically for people with HL (Bannister et al., 2024).

There is also an opportunity to gather more systematic data about use and uptake of music programs, especially in live and high-level settings, including the extent to which users are happy to adjust settings “on the go,” perhaps via a phone app. Research may also clarify the degree to which general versus personalized approaches apply, depending on the individual and their technology use. It would be useful to develop more refined tests of music perception with HAs or even to develop a standard musical test signal, akin to the International Speech Test Signal (Holube et al., 2010). Whilst this would be challenging due to the diverse acoustic properties of different genres (Lesimple et al. 2024), a test that includes culturally diverse genres and stimuli, with different spectral/dynamic ranges and source segregation demands, which could be tailored to patients’ preferences, would be a beneficial tool for fitting HAs for music. Bringing a musical instrument into the clinic may be a useful approach for musicians.

Finally, this study highlights the role of audiology practitioners in maximizing HA use and adoption via successful outcomes for music listening. Emerging data suggest that audiologists’ confidence in providing advice around fitting for music remains low, due to a lack of time, training, resources and incentives related to the topic (Greasley et al., 2020; Smith et al., 2023). The present HA user data indicating that discussions with audiologists were generally not very or not at all helpful for music listening, support this need. While specialist guidance does exist (AAA, 2020; Chasin, 2022; Crook et al., 2018), there is a lack of theoretical framework to guide the provision, adoption, uptake and success of such advice at scale. Research should strengthen the empirical basis for clinical advice with sufficiently large samples to confirm guidance strategies for general listening and personalized fitting (e.g., musicians or where engagement with music affects quality of life). Beyond technical guidance, research should seek to link psychological aspects of engagement (e.g., expectations, persistence, growth mindsets) with successful music listening outcomes, further supporting audiologists’ confidence with music listening conversations and supporting increased adoption of HAs for music.

## Supplemental Material

sj-docx-1-tia-10.1177_23312165251396517 - Supplemental material for Using Hearing Aids for Music: A UK Survey of Challenges and Strategies Supplemental material, sj-docx-1-tia-10.1177_23312165251396517 for Using Hearing Aids for Music: A UK Survey of Challenges and Strategies by Alinka E. Greasley, Amy V. Beeston, Robert J. Fulford, Harriet Crook, Jackie M. Salter, Robin Hake and Brian C. J. Moore in Trends in Hearing

## Abbreviations

AAA: American Academy of Audiology
AI: artificial intelligence
AIC: akaike information criterion
ALD: assistive listening devices
ANOVA: analysis of variance
BAHA: bone-anchored hearing aids
BATOD: British Association of Teachers of the Deaf
BCHI: bone conduction hearing instruments
BCHD: bone conduction hearing devices
BIC: Bayesian information criterion
BSA: British Society of Audiology
BSL: British sign language
CI: confidence interval
CIC: completely in the canal
CLMM: cumulative link mixed models
EIHMA: European Hearing Instrument Manufacturers Association
HA: hearing aid
HAfM: hearing aids for music
Hd: hearing loss *data* (audiometric data)
Hm: hearing loss *memory* (memory of how audiologist categorized hearing)
Hs: hearing loss *speech* (speech intelligibility in everyday contexts without hearing aids)
HL: hearing loss
ICC: intraclass correlation coefficient
IFPI: International Federation of the Phonographic Industry
ITC: in-the-canal
ML: machine learning
nAGQ: number of adaptive Gauss-Hermite quadrature points
NIHL: noise-induced hearing loss
NHS: national health service
OR: odds ratio
PCA: principal components analysis
PTA: pure tone audiometry
PVAR: performance, visual arts and communications
RNID: Royal National Institute for the Deaf
RTE: receiver-in-the-ear
SE: standard error
SM: supplementary materials
WHO: World Health Organisation
