# Supplementary material for: Using Hearing Aids for Music: A UK Survey of Challenges and Strategies
Source: Trends Hear. 2026 Jan 22;30:23312165251396517. doi: 10.1177/23312165251396517 (PMC12833179; doi:10.1177/23312165251396517)
Supplement: sj-docx-1-tia-10.1177_23312165251396517 - Supplemental material for Using Hearing Aids for Music: A UK Survey of Challenges and Strategies [file sj-docx-1-tia-10.1177_23312165251396517.docx]

### Supplementary materials – Using hearing aids for music

### Table of Contents

[Table of Contents 1](#_Toc211461314)

[SM1. Questionnaire 3](#_Toc211461315)

[SM2. List of participating NHS Trusts (N=37) 23](#_Toc211461316)

[SM3 Model fit statistics for cumulative link mixed models 24](#_Toc211461317)

[SM4 List of themes and codes in NVivo stemming from thematic analysis of open-ended responses (N=1,507. Code: Diffw = Difficulties with. Aud = Audiologist, HA = Hearing Aids, ALD = Assistive Listening Devices, HL = Hearing Loss, vln = violin, vc = violincello) 30](#_Toc211461318)

[SM5a. Age distribution (N=1,507) 31](#_Toc211461319)

[SM5b. Age x Gender distribution (n=1,503, *removing ‘Prefer not to say’ n=4*) 32](#_Toc211461320)

[SM6. Musical training score (Min 0, Max 20, n=1,507)(reflects total score where musical activities maximum score = 7, music educational qualifications maximum score = 6, and instrumental performance history maximum score = 7) 33](#_Toc211461321)

[SM7a. Musical engagement scores (N=1,507) showing rated agreement with seven engagement items. 34](#_Toc211461322)

[SM7b. Distribution of scores on a 6-item engagement scale (NB: using music streaming removed) where -12 indicates strong disagreement with all items, 0 is neutral, and +12 indicates strong agreement with all items (N=1,507). 35](#_Toc211461323)

[SM7c. Overall musical engagement score for each hearing loss level (N=1,425, max score = 30, **p<.001). 36](#_Toc211461324)

[SM7d. Likelihood of avoiding music listening for each hearing loss level (N=1,425) 37](#_Toc211461325)

[SM8a Preference ratings for 18 styles, where ‘0’ is neither disagree nor agree liking (NB: N varies across styles as participants were given the option not to rate styles they did not know or listen to, minimum score = -0.85, maximum score = 0.98). 38](#_Toc211461326)

[SM8b. Preference ratings for each HL level for four of the 18 styles, selected because those with more severe HL reported lower preferences (**p*<.05, ***p*<.01). 39](#_Toc211461327)

[SM8c. Importance of musical features for music appreciation (N=1,434) where ‘0’ is neither disagree nor agree liking (NB: N varies across styles as participants were given the option not to rate styles they did not know or listen to, minimum score = -0.16, maximum score = 1.09). 40](#_Toc211461328)

[SM9. Dome type statistics 41](#_Toc211461329)

[SM10. Music program statistics. A) Do you have a music program? B) How often do you use your music program? 42](#_Toc211461330)

[SM11. Listening to recorded music descriptives overall and for each hearing loss level. 43](#_Toc211461331)

[SM12. Listening to live music descriptives overall and for each hearing loss level. 44](#_Toc211461332)

[SM13. Descriptive data and paired comparisons for Helpfulness, Difficulties and Strategies in recorded and live music contexts. 45](#_Toc211461333)

[SM14 Helpfulness of hearing aids, difficulties experienced and strategies in recording and live music settings – inferential tests summary 50](#_Toc211461334)

### SM1. Questionnaire

Note: All questions had the option to Show BSL video.

**Inclusion/exclusion criteria and consent**

1. Do you have a hearing loss identified by an audiologist?

Yes

No: You are not able to take part in the survey. Thank you for your time.

2.Do you use hearing aids or Bone Anchored Hearing Aids for more than an hour a

day?

Yes

No: You are not able to take part in the survey. Thank you for your time.

3.Do you have a cochlear implant?

No

Yes. We are aware that some cochlear implant users wear a hearing aid in their other

ear but the survey is about people who only use hearing aids. If you would like to be

involved in future surveys about music that include cochlear implants please email

[email address removed]. Thank you very much for your time.

4.Are you aged 18 or over?

Yes

No: You are not able to take part in the survey. Thank you for your time.

5.'I am happy to take part in the project and for my answers to be used for this project. I

know that my answers will be discussed within the research team but that I will not be

identified in any publications and my answers will be confidential'

Yes (This will take you to Section 4, the start of the survey).

**About you**

1. How old are you?

2. What gender are you?

Female / Male / Prefer not to say

3. Please indicate the highest academic qualification you are currently studying or have achieved.

No qualification GCSEs/O­Levels

A­Levels/Diploma/Baccalaureate/any other final school exam

Undergraduate degree (e.g. BA/BMus/BSc)

Postgraduate degree (e.g. MA/MPhil/MMus, PhD/DPhil, any other doctorate)

Other (Please Specify)

4. Where do you live?

East Midlands

East of England

Greater London

North East England

North West England

South East England

South West England

West Midlands

Yorkshire and the Humber

Scotland

Wales

Northern Ireland

Other (Please Specify)

Your experience of music.

**This section asks you about your engagement with music and any musical training you may have had.**

It has 9 questions.

5. How much do you agree with the following general statements about your engagement with music?

|  | Strongly disagree | Disagree | Neither disagree nor agree | Agree | Strongly agree |
| --- | --- | --- | --- | --- | --- |
| 1. Music is very important to me |  |  |  |  |  |
| 2. I listen to music as often as possible |  |  |  |  |  |
| 3. I prefer listening to music I have chosen myself |  |  |  |  |  |
| 4. I have a large music collection (e.g. hundreds of MP3s, CDs, vinyl) |  |  |  |  |  |
| 5. I regularly stream music (e.g. Spotify, LastFM) |  |  |  |  |  |
| 6. I like encouraging others to listen to the music that I like |  |  |  |  |  |
| 7. I like having conversations with others about the music that I like |  |  |  |  |  |

6. Do you avoid listening to music because of your hearing loss and/or hearing aids?

Not at all

Occasionally

Sometimes

Often

All the time

7. Have you ever done any of the following? (tick all that apply)

Learned to play one or more instruments

Had singing lessons

Improvised music

Composed music

Conducted music

Played or sung in a musical ensemble (e.g. chamber music, orchestra, choir)

Mixed on decks (i.e. as a Disc Jockey)

Signed song performances

None of the above

8.What is the highest level of music education you have (or are currently completing)?

No training

GCSE music

A­Level music

Grade 8 or equivalent (e.g. ABRSM, Trinity)

Undergraduate­level diploma/Degree

Postgraduate­level diploma/Degree

Doctorate in Music/Doctorate in Musical Arts/Any other

Other (Please Specify)

9. Which of the following statements best describes you? (tick one)

I have never played an instrument or sung

I used to play an instrument (or sing) years ago

I currently play one instrument (or sing) to a basic level

I currently play one instrument (or sing) to a moderate level (e.g Grade 6­7)

I currently play one instrument (or sing) to a high level (e.g. minimum Grade 8)

I currently play two or more instruments (or sing) to a basic level

I currently play two or more instruments (or sing) to a moderate level (e.g Grade 6­7)

I currently play two or more instruments (or sing) to a high level (e.g. minimum Grade 8)

10. If you would like to give us further details, please do: *(For example, if you play one or more instruments, what are they, how long have you played them for, and what standard did you reach? If you used to play, please indicate what, how long for, and how long it is since you played).*

11. Have you ever worked in a musical field (e.g. performance, education, composition, technology, production, community music, therapy)?

Yes

No

12. If yes, has your hearing loss affected your musical work?

Yes

No

13. Please give us more detail about how your hearing loss affected your musical work if you would like to.

**This section asks about your musical preferences and uses of music**

This section asks you about your enjoyment of different styles of music and why you listen to music. It has 3 questions.

14. Please rate your liking for the styles of music you listen to:

|  | Dislike greatly | Dislike | Neutral | Like | Like greatly |
| --- | --- | --- | --- | --- | --- |
| 1. Blues |  |  |  |  |  |
| 2. Choral music |  |  |  |  |  |
| 3. Classical (chamber) |  |  |  |  |  |
| 4. Classical (orchestral) |  |  |  |  |  |
| 5. Country |  |  |  |  |  |
| 6. Electronic/dance music |  |  |  |  |  |
| 7. Folk |  |  |  |  |  |
| 8. Heavy metal |  |  |  |  |  |
| 9. Hip­hop/Rap |  |  |  |  |  |
| 10. Hymns/church music |  |  |  |  |  |
| 11. Indie |  |  |  |  |  |
| 12. Jazz |  |  |  |  |  |
| 13. Musical theatre |  |  |  |  |  |
| 14. Opera |  |  |  |  |  |
| 15. Pop |  |  |  |  |  |
| 16. Rock |  |  |  |  |  |
| 17. Soul/R&B |  |  |  |  |  |
| 18. Signed song |  |  |  |  |  |

15. Are the following musical features important for you to enjoy music?

|  | Strongly disagree | Disagree | Neither disagree nor agree | Agree | Strongly agree |
| --- | --- | --- | --- | --- | --- |
| 1. Lyrics |  |  |  |  |  |
| 2. Voice/ singing |  |  |  |  |  |
| 3. Type of instrument(s) |  |  |  |  |  |
| 4. Synthesisers (electronic instruments) |  |  |  |  |  |
| 5. Harmony (how two or more notes played at the same time sound together) |  |  |  |  |  |
| 6. Rhythm/beat |  |  |  |  |  |
| 7. Tempo/speed |  |  |  |  |  |
| 8. Loudness/ dynamics |  |  |  |  |  |

16. Do you listen to music for the following reasons?

|  | Strongly disagree | Disagree | Neither disagree nor agree | Agree | Strongly agree |
| --- | --- | --- | --- | --- | --- |
| 1. To help me think/concentrate |  |  |  |  |  |
| 2. To distract me |  |  |  |  |  |
| 3. To help me relax |  |  |  |  |  |
| 4. To help me carry out daily activities (e.g. cooking, cleaning) |  |  |  |  |  |
| 5. In my work (e.g. teaching, forthcoming performance) |  |  |  |  |  |
| 6. To help me exercise |  |  |  |  |  |
| 7. To create the right atmosphere |  |  |  |  |  |
| 8. To bring back certain memories |  |  |  |  |  |
| 9. To create, accentuate or change an emotion/mood |  |  |  |  |  |
| 10. For pleasure |  |  |  |  |  |
| 11. For other people's pleasure |  |  |  |  |  |
| 12. To help pass time |  |  |  |  |  |
| 13. To help me feel less alone |  |  |  |  |  |
| 14. Out of habit |  |  |  |  |  |
| 15. To help with tinnitus |  |  |  |  |  |

**About your hearing**

**This section will ask about the type and nature of your hearing. It has 5 questions.**

If you have your most recent audiogram and are happy to send a copy to us, that would be very helpful. Details of where to send it are included at the end of the survey.

17. Is your hearing loss in:

One ear

Both ears

18. Which of these best describes your experience listening **without your hearing aids**? (Please pick one)

1. When talking to one person in a quiet room, I can usually understand conversation. I sometimes have problems hearing speech in noisy environments (e.g. pub) and in group conversations.

2. I have difficulty understanding speech and often have to ask people to repeat what they have said when talking to them in person or on the telephone. In noisy environments, I find it difficult to follow conversations. I have to turn the TV and radio up to hear them clearly.

3. I cannot hear speech without using hearing aids. I use lip­reading to support my understanding. When I am wearing my hearing aids, it can still be difficult to follow conversation in a noisy environment or follow group conversations. It is also difficult to use the telephone.

4. Even when I am wearing my hearing aids, I cannot hear speech or shouted words clearly and I use lip­reading and/or BSL for communication.

19. What is your overall hearing loss as described by your audiologist?

Mild

Moderate

Severe

Profound

Don't know

20. When and how did your hearing loss start?

I’ve been deaf since birth

It was a sudden change

It happened gradually (over many months /years)

It happened as I got older

I don't remember

21. If you would like to tell us more about your hearing loss, please do so: For example: genetic (a history of deafness in your family); age­related hearing loss; noise-induced (machinery, professional musician); trauma ­ sudden acquired hearing loss; medication).

**Your current Hearing Aids**

This section asks about your current hearing aids. It has 10 questions.

22. Do you wear one or two hearing aids?

One

Two

23. How long have you worn hearing aids?

Less than 3 months

3­6 months

6­12 months

1­2 years

3­4 years

5 years or more

24. How long have you had your current hearing aids?

Less than 3 months

3­6 months

6-12 months

1-2 years

3-4 years

5 years or more


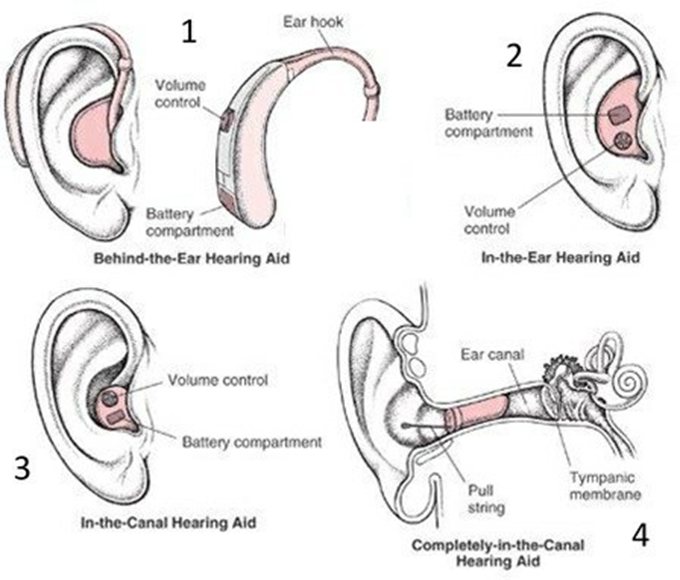


Picture 1: Behind the ear (BTE) (e.g. with earmould in the ear or with thin tubing and a soft dome)

Picture 2: Receiver in the ear (RITE) (sits in the ear canal and the shell of the ear)

Picture 3: In the canal (ITC) (the whole hearing aid fits inside the ear canal, but it can be seen)

Picture 4: Completely in the canal (CIC) (this fits further into your ear canal than an ITC aid, and is almost invisible)

Bone Conducted Hearing Instrument (BCHI) or Bone Anchored Hearing Aid (BAHA) Not sure / Don't know

25. Using the pictures above, what type of hearing aids do you use when you are listening to music?

26. What sort of dome or mould does your hearing aid have?


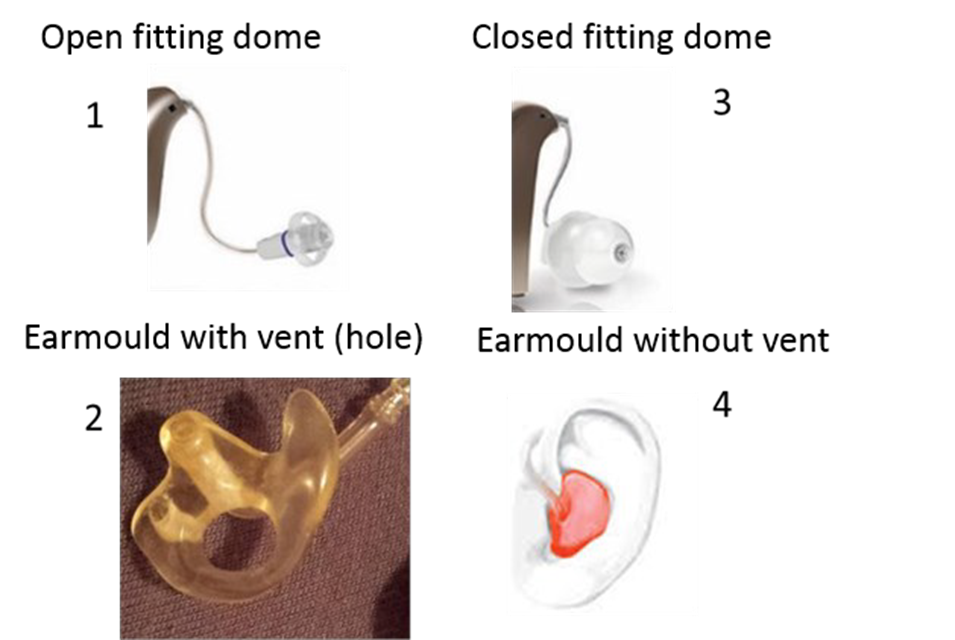


Picture 1: Open: soft dome with openings

Picture 2: Open: earmould with vent

Picture 3: Closed: sealed dome

Picture 4: Closed: earmould with no vent

Not applicable (e.g. in the canal hearing aid)

Don't know

27. If you know the make or model of your hearing aid please add it here (e.g. Phonak "Nathos" or Oticon "Synergy").

28. Who fitted your current hearing aids?

National Health Service (NHS)

Non­NHS

Not applicable (overseas respondent)

29. Do you use the volume control on your hearing aid(s)?

Yes

Sometimes

No

Not applicable: I don't have a volume control. My HAs do this automatically

30. Do you have a special program in your hearing aid(s) for listening to music?

Yes

No

Don't know

31. If yes, how frequently do you use your music program?

Never

Occasionally

Sometimes

Often

All the time

**Listening to music**

The following sections ask you about how and when you listen to music with your hearing aids, and whether you use any assistive listening devices.

Sections 9 and 10 ask about listening to recorded music. This might be at home, in the car, on a personal listening device (e.g. MP3 player, Ipod) the radio or the television. If you do not listen to recorded music, you will be taken to Section 11.

Sections 11 and 12 ask about attending live musical performances.

Please note: Some questions are repeated for Section 10 (recorded) and 12 (live). This gives us different information about each setting which is important to our research.

32. Do you listen to RECORDED music with your hearing aids?

Yes

Sometimes

No

Not applicable ­ I don't listen to recorded music

33. If you have answered 'no' or 'not applicable', please tell us more here:

**Listening to RECORDED music**

This section has 12 questions.

34. Roughly how many hours do you spend listening to RECORDED music in an average week?

None

1­2 hours

3­4 hours

5­10 hours

10­20 hours

More than 20 hours

35. How often do you listen to music in the following ways?

|  | Never | Occasionally | Sometimes | Often | All the time |
| --- | --- | --- | --- | --- | --- |
| 1. Through loudspeakers at home (e.g. recorded music / TV / Radio) |  |  |  |  |  |
| 2. Using headphones |  |  |  |  |  |
| 3. Car stereo |  |  |  |  |  |

36. Do you listen to music in these ways with or without hearing aids?

|  | With HAs | Sometimes with HAs, sometimes without HAs | Without HAs | Not applicable (I don’t listen in this setting) |
| --- | --- | --- | --- | --- |
| 1. Through loudspeakers at home (e.g. recorded music / TV / Radio) |  |  |  |  |
| 2. Using headphones |  |  |  |  |
| 3. Car stereo |  |  |  |  |

37. Do you listen to RECORDED music with your hearing aids and with any assistive listening devices (e.g. loops or streamer)?

Yes

No (Go to Q44)

38. How often do you listen to music **with your hearing aids** and one of the following assistive listening devices (ALDs)?

|  | Never | Occasionally | Sometimes | Often | All the time |
| --- | --- | --- | --- | --- | --- |
| 1. Loop (T setting) |  |  |  |  |  |
| 2.Inductive earhooks (T setting) |  |  |  |  |  |
| 3. Direct Audio Input (with a lead) |  |  |  |  |  |
| 4. With a radio aid system (e.g. Phonak Roger Pen) |  |  |  |  |  |
| 5. Streaming direct to the hearing aid(s) (e.g. Bluetooth, wireless) (no lead) |  |  |  |  |  |

39. When you are listening to RECORDED music how helpful are your hearing aid(s) for:

|  | Not at all helpful | Somewhat helpful | Medium | Fairly helpful | Very helpful | Don’t know |
| --- | --- | --- | --- | --- | --- | --- |
| 1. Hearing the melody |  |  |  |  |  |  |
| 2. Hearing the bassline |  |  |  |  |  |  |
| 3. Hearing the singer |  |  |  |  |  |  |
| 4. Hearing and understanding lyrics |  |  |  |  |  |  |
| 5. Picking out individual instruments (e.g. pick out guitar in band; pick out oboe in orchestra) |  |  |  |  |  |  |

40. When you are listening to RECORDED music with your hearing aids how often do you experience the following difficulties?

|  | Never | Occasionally | Sometimes | Often | All the time | Don’t know / Not applicable |
| --- | --- | --- | --- | --- | --- | --- |
| 1. Distortion |  |  |  |  |  |  |
| 2. Too much bass |  |  |  |  |  |  |
| 3. Too much treble |  |  |  |  |  |  |
| 4. Feedback (a whistling sound) |  |  |  |  |  |  |
| 5. Sudden changes in loudness |  |  |  |  |  |  |
| 6. Discomfort from loud sounds |  |  |  |  |  |  |

41. Please tell us about any other difficulties you experience when listening to RECORDED music with your hearing aids:

42. When you are listening to RECORDED music with your hearing aids how often do you:

|  | Never | Occasionally | Sometimes | Often | All the time | My HA does this automatically |
| --- | --- | --- | --- | --- | --- | --- |
| 1. Adjust the Volume control on your hearing aid(s) |  |  |  |  |  |  |
| 2. Change program (music or otherwise) on your hearing aid(s) |  |  |  |  |  |  |

43. When you are listening to RECORDED music with your hearing aids how often do you:

|  | Never | Occasionally | Sometimes | Often | All the time |
| --- | --- | --- | --- | --- | --- |
| Move in relation to the sound source (e.g. away from speakers) when possible |  |  |  |  |  |

44. When listening to RECORDED music with your hearing aids how easy is it to listen to the following styles of music?

|  | Very difficult | Sometimes difficult | Medium | Fairly easy | Very easy | Do not listen to this style |
| --- | --- | --- | --- | --- | --- | --- |
| 1. Blues |  |  |  |  |  |  |
| 2. Choral music |  |  |  |  |  |  |
| 3. Classical (chamber) |  |  |  |  |  |  |
| 4. Classical (orchestral) |  |  |  |  |  |  |
| 5. Country |  |  |  |  |  |  |
| 6. Electronic/dance music |  |  |  |  |  |  |
| 7. Folk |  |  |  |  |  |  |
| 8. Heavy metal |  |  |  |  |  |  |
| 9. Hip­hop/Rap |  |  |  |  |  |  |
| 10. Hymns/church music |  |  |  |  |  |  |
| 11. Indie |  |  |  |  |  |  |
| 12. Jazz |  |  |  |  |  |  |
| 13. Musical theatre |  |  |  |  |  |  |
| 14. Opera |  |  |  |  |  |  |
| 15. Pop |  |  |  |  |  |  |
| 16. Rock |  |  |  |  |  |  |
| 17. Soul/R&B |  |  |  |  |  |  |
| 18. Signed song |  |  |  |  |  |  |

45. On a scale of 1­10, how helpful are your hearing aid(s) when listening to RECORDED music? (1 'Not at all helpful, they make it more difficult to listen to music' through to 10 'Extremely helpful, they allow me to enjoy music as I would like')

| 1 | 2 | 3 | 4 | 5 | 6 | 7 | 8 | 9 | 10 |
| --- | --- | --- | --- | --- | --- | --- | --- | --- | --- |

**Listening to LIVE music with your hearing aids**

The next two sections ask about your experience listening to LIVE music with your hearing aids (e.g. at concerts or gigs).

46. When you attend LIVE music events, do you wear your hearing aids?

Yes

Sometimes

No

Not applicable ­ I don't attend live events

47. If you have answered 'no' or 'not applicable', please tell us more here:

**Listening to LIVE music (when attending events)**

This section has 10 questions.

48. How many live music events have you attended in the past twelve months?

0

1

2

3

4

6

7­10

11 or more

49. What type of LIVE events do you attend?

Acoustic music (e.g. uses instruments and no electronic devices (e.g. microphones, speakers) to make music louder)

Amplified (e.g. music is made louder through use of electronic devices e.g. microphones, speakers)

100% acoustic

75% acoustic (25% amplified)

50% acoustic (50% amplified)

25% acoustic (75% amplified)

100% amplified

50. Do you listen to live music with or without hearing aids?

|  | With HAs | Sometimes with HAs, sometimes without HAs | Without HAs | Not applicable |
| --- | --- | --- | --- | --- |
| 1. Live music: acoustic |  |  |  |  |
| 2. Live music: amplified |  |  |  |  |
| 3. When playing an instrument/ singing |  |  |  |  |

51. When listening to LIVE music, how helpful are your hearing aid(s) for:

|  | Not at all helpful | Somewhat helpful | Medium | Fairly helpful | Very helpful | Don’t know |
| --- | --- | --- | --- | --- | --- | --- |
| 1. Hearing the melody |  |  |  |  |  |  |
| 2. Hearing the bassline |  |  |  |  |  |  |
| 3. Hearing the singer |  |  |  |  |  |  |
| 4. Hearing and understanding lyrics |  |  |  |  |  |  |
| 5. Picking out individual instruments (e.g. pick out guitar in band; pick out oboe in orchestra) |  |  |  |  |  |  |

52. When listening to LIVE music with your hearing aids do you experience any of the following difficulties?

|  | Never | Occasionally | Sometimes | Often | All the time | Don’t know / Not applicable |
| --- | --- | --- | --- | --- | --- | --- |
| 1. Distortion |  |  |  |  |  |  |
| 2. Too much bass |  |  |  |  |  |  |
| 3. Too much treble |  |  |  |  |  |  |
| 4. Feedback (a whistling sound) |  |  |  |  |  |  |
| 5. Sudden changes in loudness |  |  |  |  |  |  |
| 6. Discomfort from loud sounds |  |  |  |  |  |  |

53. When you are listening to LIVE music with your hearing aids how often do you:

|  | Never | Occasionally | Sometimes | Often | All the time | My HA does this automatically |
| --- | --- | --- | --- | --- | --- | --- |
| 1. Adjust the Volume control on your hearing aid(s) |  |  |  |  |  |  |
| 2. Change program (music or otherwise) on your hearing aid(s) |  |  |  |  |  |  |

54. When you are listening to LIVE music with your hearing aids how often do you:

|  | Never | Occasionally | Sometimes | Often | All the time |
| --- | --- | --- | --- | --- | --- |
| Move in relation to the sound source (e.g. away from speakers) when possible |  |  |  |  |  |

55. When listening to LIVE music with your hearing aids how easy is it to listen to the following styles of music?

|  | Very difficult | Sometimes difficult | Medium | Fairly easy | Very easy | Do not listen to this style |
| --- | --- | --- | --- | --- | --- | --- |
| 1. Blues |  |  |  |  |  |  |
| 2. Choral music |  |  |  |  |  |  |
| 3. Classical (chamber) |  |  |  |  |  |  |
| 4. Classical (orchestral) |  |  |  |  |  |  |
| 5. Country |  |  |  |  |  |  |
| 6. Electronic/dance music |  |  |  |  |  |  |
| 7. Folk |  |  |  |  |  |  |
| 8. Heavy metal |  |  |  |  |  |  |
| 9. Hip­hop/Rap |  |  |  |  |  |  |
| 10. Hymns/church music |  |  |  |  |  |  |
| 11. Indie |  |  |  |  |  |  |
| 12. Jazz |  |  |  |  |  |  |
| 13. Musical theatre |  |  |  |  |  |  |
| 14. Opera |  |  |  |  |  |  |
| 15. Pop |  |  |  |  |  |  |
| 16. Rock |  |  |  |  |  |  |
| 17. Soul/R&B |  |  |  |  |  |  |
| 18. Signed song |  |  |  |  |  |  |

56. On a scale of 1­10, how helpful are your hearing aid(s) when listening to LIVE music? (1 'Not at all helpful, they make it more difficult to listen to music' through to 10 'Extremely helpful, they allow me to enjoy music as I would like')

| 1 | 2 | 3 | 4 | 5 | 6 | 7 | 8 | 9 | 10 |
| --- | --- | --- | --- | --- | --- | --- | --- | --- | --- |

57. Please tell us more about listening to LIVE music with your hearing aid(s).

**Discussing music with your audiologist**

This section asks about any discussions you may have had with your audiologist about music.

It has 5 questions.

58. When did you last visit your audiologist? Within the last...

3 months

6 months

1 year

2 years

3 years

4 years

5 years

More than five years ago

59. Have you ever talked with your audiologist about listening to music using your hearing aids?

Yes

No (Please skip to the next page)

60. Who raised the topic?

Me

My audiologist

I can't remember

61. Did the discussion(s) improve your music listening experiences?

Not at all

Not very much

Yes, a little

Yes, a lot

62. Please tell us more about this if you would like:

**Final details**

63. Would you like to be entered into the prize draw for the chance to win one of three £75 cash prizes?

Yes

No

64. Would you like information about future studies?

Yes

No

65. Did you use any of the BSL videos?

Yes

No

66. If you answered yes to either of the above questions, or will be sending us a copy of your audiogram, please leave your name and email address

Name (e.g. John Smith):

Email address:

If you have a copy of your most recent audiogram please either:

1. Scan it and email a copy to: [email redacted for blind review]

OR

2. Photocopy it and post it to

[Address redacted]

If you post it to us, please include your name and email address on the audiogram and

add your name and email address above.

### SM2. List of participating NHS Trusts (N=37)

Aintree University Hospital NHS Foundation Trust

Airedale NHS Foundation Trust

Blackpool Teaching Hospitals NHS Foundation Trust

Brighton and Sussex University Hospitals NHS Trust

Cambridge University Hospitals NHS Foundation Trust

Chesterfield Royal Hospital NHS Foundation Trust

City hospitals Sunderland NHS Foundation Trust

County Durham and Darlington NHS Foundation Trust

East Cheshire NHS Trust

East Sussex Healthcare Trust

Harrogate and District NHS Foundation Trust

Ipswich Hospital Healthcare Trust

Isle of Wight NHS Trust

James Paget University Hospitals NHS Foundation Trust

Kingston Hospital NHS Foundation Trust

Leeds Teaching Hospitals NHS Foundation Trust

Medway NHS Foundation Trust

Milton Keynes University Hospital NHS Foundation Trust

North West Anglia NHS Foundation Trust

Royal Cornwall Hospitals NHS Trust

Salford Royal NHS Foundation Trust

Sheffield Teaching Hospitals NHS Foundation Trust

Shrewsbury and Telford Hospital NHS Trust

Southend University Hospital NHS Foundation Trust

Stockport NHS Foundation Trust

Tameside and Glossop Integrated Care NHS Foundation Trust

The James Cook University Hospital

The Rotherham NHS Foundation Trust

University College London Hospitals NHS Trust

University Hospital of South Manchester NHS Foundation Trust

University Hospitals Birmingham NHS Foundation Trust

University Hospitals of Leicester NHS Trust

Walsall Healthcare NHS Trust

West Suffolk NHS Foundation Trust

Western Sussex Hospitals NHS Foundation Trust

Wrightington, Wigan and Leigh NHS Foundation Trust

Yeovil district NHS Foundation Trust

### SM3 Model fit statistics for cumulative link mixed models

**Table 1a: Model fit statistics for cumulative link mixed models (CLMM) predicting helpfulness ratings**

| **Model** | **Variables** | **AIC** | **BIC** | **R2**  **(cond.)** | **R2**  **(marg.)** |
| --- | --- | --- | --- | --- | --- |
| Model H1 | ~ Item + (1 \| ID) | 21171.13 | 21234.28 | .631 | .018 |
| Model H2 | ~ Item * HL + (1 \| ID) | 20932.31 | 21100.72 | .647 | .045 |
| **Model H3** | **~ Item * HL + Setting + (1 \| ID)** | **20865.05** | **21040.49** | **.652** | **.048** |
| Model H4 | ~ Item * HL + Setting + Age + (1 \| ID) | 20866.91 | 21049.37 | .652 | .048 |
| Model H5 | ~ Item * HL + Setting + Age + Gender + (1 \| ID) | 20867.33 | 21063.82 | .652 | .051 |
| Model H6 | ~ Item * HL + Setting + Age + Musical training + (1 \| ID) | 20867.3 | 21063.8 | .652 | .051 |

*Notes.* Total N = 879; Sigma = 3.24. Helpfulness ratings (“*When listening to music, how helpful are HAs for hearing ….?*”) implemented as ordinal [1 = Not at all helpful, 5 = Very helpful]; Items type (Item) as categorical [melody, bass, singer, lyrics, instrument] with ‘bass’ as the reference category; Hearing loss (HL) as ordinal [1 = mild HL, 4 = profound HL]; Music setting (Setting) [Live vs. Recorded] with ‘Recorded’ as the reference category; Gender as categorical [Female, Male, Prefer not to say] with ‘Female’ as reference category; Age as numerical [18–93]; Musical training as numerical [0–19]. The final model for analysis is highlighted in bold font.

**Table 1b: Model fit statistics for the final Model (H3) predicting helpfulness ratings**

|  | **Helpfulness rating** | | |
| --- | --- | --- | --- |
| *Predictors* | *Odds Ratios* | *CI* | *p* |
| 1\|2 | 0.02 | 0.02 – 0.04 | **<0.001** |
| 2\|3 | 0.19 | 0.13 – 0.27 | **<0.001** |
| 3\|4 | 0.78 | 0.54 – 1.13 | 0.189 |
| 4\|5 | 5.43 | 3.73 – 7.91 | **<0.001** |
| Item: Instruments | 0.47 | 0.36 – 0.61 | **<0.001** |
| Item: Lyrics | 0.38 | 0.29 – 0.50 | **<0.001** |
| Item: Melody | 1.48 | 1.14 – 1.91 | **0.003** |
| Item: Singer | 1.21 | 0.93 – 1.57 | 0.158 |
| HL: Linear trend | 1.46 | 0.56 – 3.80 | 0.435 |
| HL: Quadratic trend | 0.87 | 0.41 – 1.82 | 0.708 |
| HL: Cubic trend | 0.94 | 0.61 – 1.44 | 0.768 |
| Music setting: Live | 0.69 | 0.63 – 0.75 | **<0.001** |
| Instruments x HL: Linear | 0.10 | 0.05 – 0.19 | **<0.001** |
| Lyrics x HL: Linear | 0.06 | 0.03 – 0.13 | **<0.001** |
| Melody x HL: Linear | 0.43 | 0.22 – 0.84 | **0.013** |
| Singer x HL: Linear | 0.26 | 0.13 – 0.52 | **<0.001** |
| Instruments x HL: Quadratic | 0.61 | 0.36 – 1.04 | 0.068 |
| Lyrics x HL: Quadratic | 0.40 | 0.23 – 0.70 | **0.001** |
| Melody x HL: Quadratic | 0.55 | 0.33 – 0.93 | **0.026** |
| Singer x HL: Quadratic | 0.49 | 0.29 – 0.82 | **0.007** |
| Instruments x HL: Cubic | 1.36 | 0.99 – 1.85 | 0.054 |
| Lyrics x HL: Cubic | 1.46 | 1.06 – 2.01 | **0.020** |
| Melody x HL: Cubic | 1.04 | 0.77 – 1.42 | 0.791 |
| Singer x HL: Cubic | 1.24 | 0.91 – 1.68 | 0.167 |
|  |  |  |  |
| **Random Effects** |  |  |  |
| σ^2^ | 3.29 |  |  |
| τ_00_ _Respondent_ID_ | 5.70 |  |  |
| ICC | 0.63 |  |  |
| N _Respondent_ID_ | 877 |  |  |
| Observations | 8245 |  |  |
| Marginal R^2^ / Conditional R^2^ | 0.049 / 0.652 |  |  |

*Notes.* Odds ratios (OR) with 95% confidence intervals (CI) are reported. Predictors were dummy coded with “Bass” as the reference category for ‘Items’ and “Recorded” as the reference category for ‘Music setting’. Hearing loss (HL) severity was entered as orthogonal polynomial contrasts (linear, quadratic, cubic). A logit link was used. σ² = residual variance; τ₀₀ = random intercept variance at the respondent level; ICC = intraclass correlation coefficient. Bold font indicates significant predictors at *p* < .05.

**Table 2a: Model fit statistics for cumulative link mixed models (CLMM) predicting difficulty ratings**

| **Model** | **Variables** | **AIC** | **BIC** | **R2**  **(cond.)** | **R2**  **(marg.)** |
| --- | --- | --- | --- | --- | --- |
| Model D1 | ~ Item + (1 \| ID) | 25074.16 | 25146.00 | 0.457 | .039 |
| Model D2 | ~ Item * HL + (1 \| ID) | 24986.10 | 25187.24 | 0.464 | .059 |
| Model D3 | ~ Item * HL + Setting + (1 \| ID) | 24959.87 | 25168.19 | 0.466 | .061 |
| **Model D4** | **~ Item * HL + Setting + Age + (1 \| ID)** | **24934.71** | **25150.21** | **0.465** | **.075** |
| Model D5 | ~ Item * HL + Setting + Age + Gender + (1 \| ID) | 24934.10 | 25163.98 | 0.465 | .077 |
| Model D6 | ~ Item * HL + Setting + Age + Musical training + (1 \| ID) | 24936.49 | 25159.18 | 0.465 | .075 |

*Notes.* Total N = 879; σ² = 2.41. Difficulty ratings (“*When listening to music with HAs, how often do your experience?”)* were implemented as ordinal [1 = Never, 5 = All the time]; Item type (Item) was included as a categorical predictor [distortion, treble, discomfort, bass, feedback, volume changes] with bass as the reference category; Hearing loss (HL) severity was treated as ordinal [1 = mild HL, 4 = profound HL]; Music setting (Setting) [Live vs. Recorded] with Recorded as the reference category; Gender was included as categorical [Female, Male, Prefer not to say] with Female as reference category; Age as numerical [18–93]; Musical training as numerical [0–19]. The final model selected for analysis is highlighted in bold font.

**Table 2b: Model fit statistics for the Model (D4) predicting difficulty ratings**

|  | **Difficulty rating** | | | | |
| --- | --- | --- | --- | --- | --- |
| *Predictors* | *Odds Ratios* | *CI* | | *p* | |
| 1\|2 | 0.19 | 0.12 – 0.31 | | **<0.001** | |
| 2\|3 | 0.80 | 0.49 – 1.30 | | 0.362 | |
| 3\|4 | 4.24 | 2.59 – 6.94 | | **<0.001** | |
| 4\|5 | 27.52 | 16.68 – 45.38 | | **<0.001** | |
| Item: Discomfort | 1.27 | 0.97 – 1.67 | | 0.085 | |
| Item: Distortion | 2.65 | 2.01 – 3.50 | | **<0.001** | |
| Item: Feedback | 0.54 | 0.41 – 0.72 | | **<0.001** | |
| Item: Treble | 1.34 | 1.01 – 1.77 | | **0.045** | |
| Item: Volume Changes | 0.68 | 0.51 – 0.90 | | **0.007** | |
| HL: Linear trend | 2.36 | 1.12 – 5.00 | | **0.024** | |
| HL: Quadratic trend | 0.68 | 0.38 – 1.22 | | 0.194 | |
| HL: Cubic trend | 0.67 | 0.48 – 0.93 | | **0.018** | |
| Music setting: Live | 1.24 | 1.14 – 1.34 | | **<0.001** | |
| Age | 0.98 | 0.98 – 0.99 | | **<0.001** | |
| Discomfort x HL: Linear | 0.28 | 0.14 – 0.57 | | **<0.001** | |
| Distortion x HL: Linear | 0.66 | 0.32 – 1.35 | | 0.254 | |
| Feedback x HL: Linear | 0.23 | 0.11 – 0.49 | | **<0.001** | |
| Treble x HL: Linear | 0.39 | 0.19 – 0.81 | | **0.011** | |
| Volume Changes x HL: Linear | 0.52 | 0.25 – 1.08 | | 0.078 | |
| Discomfort x HL: Quadratic | 0.63 | 0.37 – 1.09 | | 0.098 | |
| Distortion x HL: Quadratic | 0.87 | 0.50 – 1.52 | | 0.633 | |
| Feedback x HL: Quadratic | 0.80 | 0.45 – 1.40 | | 0.431 | |
| Treble x HL: Quadratic | 0.86 | 0.49 – 1.51 | | 0.604 | |
| Volume Changes x HL: Quadratic | 0.73 | 0.42 – 1.28 | | 0.273 | |
| Discomfort x HL: Cubic | 1.37 | 1.01 – 1.87 | | **0.045** | |
| Distortion x HL: Cubic | 1.12 | 0.82 – 1.53 | | 0.488 | |
| Feedback x HL: Cubic | 1.43 | 1.03 – 1.97 | | **0.031** | |
| Treble x HL: Cubic | 1.33 | 0.97 – 1.83 | | 0.079 | |
| Volume Changes x HL: Cubic | 1.26 | 0.92 – 1.73 | | 0.156 | |
|  |  |  | |  | |
| **Random Effects** |  |  |  | |  |
| σ^2^ | 3.29 |  |  | |  |
| τ_00_ _Respondent_ID_ | 2.41 |  |  | |  |
| ICC | 0.42 |  |  | |  |
| N _Respondent_ID_ | 879 |  |  | |  |
| Observations | 9736 |  |  | |  |
| Marginal R^2^ / Conditional R^2^ | 0.075 / 0.466 |  |  | |  |

*Notes.* Odds ratios (OR) with 95% confidence intervals (CI) are reported. Predictors were dummy coded with “Bass” as the reference category for ‘Items’ and “Recorded” as the reference category for ‘Music setting’. Hearing loss (HL) severity was entered as orthogonal polynomial contrasts (linear, quadratic, cubic). A logit link was used. σ² = residual variance; τ₀₀ = random intercept variance at the respondent level; ICC = intraclass correlation coefficient. Bold font indicates significant predictors at *p* < .05.

**Table 3a: Model fit statistics for cumulative link mixed models (CLMM) predicting strategy use to improve music listening**

| **Model** | **Variables** | **AIC** | **BIC** | **R2**  **(cond.)** | **R2**  **(marg.)** |
| --- | --- | --- | --- | --- | --- |
| Model S1 | ~ Item + (1 \| ID) | 13348.44 | 13394.01 | 0.403 | 0.021 |
| Model S2 | ~ Item * HL + (1 \| ID) | 13323.25 | 13427.40 | 0.404 | 0.042 |
| Model S3 | ~ Item * HL + Setting + (1 \| ID) | 13309.74 | 13420.40 | 0.407 | 0.044 |
| Model S4 | ~ Item * HL + Setting + Age + (1 \| ID) | 13297.69 | 13414.86 | 0.407 | 0.052 |
| Model S5 | ~ Item * HL + Setting + Age + Gender + (1 \| ID) | 13301.50 | 13431.69 | 0.407 | 0.052 |
| **Model S6** | **~ Item * HL + Setting + Age + Musical training + (1 \| ID)** | **13266.60** | **13390.30** | **0.408** | **0.070** |

*Notes.* Total N = 884; σ² = 3.29. Strategy use (“*When listening to music with HAs, how often do you …?”)* was implemented as ordinal [1 = Never, 5 = All the time *excluding* ‘Hearing aid does this automatically’]; Strategy type (Item) was included as a categorical predictor [programme adjustments, volume adjustments, position adjustments] with position adjustments as the reference category; Hearing loss (HL) severity was treated as ordinal [1 = mild HL, 4 = profound HL]; Music setting (Setting) [Live vs. Recorded] with Recorded as the reference category; Gender was included as categorical [Female, Male, Prefer not to say] with Female as reference category; Age as numerical [18–93]; Musical training as numerical [0–19]. The final model selected for analysis is highlighted in bold font.

**Table 3b: Model fit statistics for the Model (S6) predicting strategy use to improve music listening**

|  | **Strategy use rating** | | | | |
| --- | --- | --- | --- | --- | --- |
| *Predictors* | *Odds Ratios* | *CI* | | *p* | |
| 1\|2 | 0.49 | 0.30 – 0.80 | | **0.005** | |
| 2\|3 | 1.58 | 0.97 – 2.57 | | 0.068 | |
| 3\|4 | 6.25 | 3.82 – 10.23 | | **<0.001** | |
| 4\|5 | 35.44 | 21.35 – 58.83 | | **<0.001** | |
| Item: Programme adjustment | 0.63 | 0.47 – 0.86 | | **0.003** | |
| Item: Volume adjustment | 1.83 | 1.39 – 2.41 | | **<0.001** | |
| HL: Linear trend | 1.75 | 0.86 – 3.57 | | 0.122 | |
| HL: Quadratic trend | 0.78 | 0.45 – 1.35 | | 0.374 | |
| HL: Cubic trend | 0.84 | 0.62 – 1.14 | | 0.269 | |
| Music setting: Live | 1.24 | 1.11 – 1.39 | | **<0.001** | |
| Age | 0.99 | 0.98 – 0.99 | | **<0.001** | |
| Musical training | 1.07 | 1.05 – 1.09 | | **<0.001** | |
| Programme adjustment x HL: Linear | 0.46 | 0.21 – 1.03 | | **0.058** | |
| Volume adjustment x HL: Linear | 1.10 | 0.54 – 2.26 | | 0.786 | |
| Programme adjustment x HL: Quadratic | 0.54 | 0.29 – 1.00 | | **0.050** | |
| Volume adjustment x HL: Quadratic | 0.87 | 0.50 – 1.51 | | 0.618 | |
| Programme adjustment x HL: Cubic | 0.82 | 0.58 – 1.14 | | 0.238 | |
| Volume adjustment x HL: Cubic | 0.92 | 0.68 – 1.26 | | 0.605 | |
| **Random Effects** |  |  |  | |  |
| σ^2^ | 3.29 |  |  | |  |
| τ_00_ _Respondent_ID_ | 1.87 |  |  | |  |
| ICC | 0.36 |  |  | |  |
| N _Respondent_ID_ | 884 |  |  | |  |
| Observations | 4936 |  |  | |  |
| Marginal R^2^ / Conditional R^2^ | 0.070 / 0.408 |  |  | |  |

*Notes.* Odds ratios (OR) with 95% confidence intervals (CI) are reported. Predictors were dummy coded with “Position adjustment” as the reference category for ‘Items’ and “Recorded” as the reference category for ‘Music setting’. Hearing loss (HL) severity was entered as orthogonal polynomial contrasts (linear, quadratic, cubic). A logit link was used. σ² = residual variance; τ₀₀ = random intercept variance at the respondent level; ICC = intraclass correlation coefficient. Bold font indicates significant predictors at *p* < .05.

### SM4 **List of themes and codes in NVivo stemming from thematic analysis of open-ended responses** (N=1,507. Code: Diffw = Difficulties with. Aud = Audiologist, HA = Hearing Aids, ALD = Assistive Listening Devices, HL = Hearing Loss, vln = violin, vc = violincello)

| **(1) Clinical observations and aspirations**  (1 1) Analogue HAs  (1 2) Desire control of HA settings  (1 3) Different HAs for musicians  (1 4) Hearing tests for music  (1 5) Listening to music in clinic  (1 6) Multiple follow-ups  (1 7) Music versus speech  **(2) Communication and networking**  (2 1) Contact with expert audiologist  (2 2) Contact with HA user organisations  (2 3) Contact with manufacturer  (2 4) Contact with musicians  (2 5) Contact with research  (2 6) Difficulties with communication  (2 7) Rare to discuss music  **(3) Environment-related difficulties**  (3 1) Loop system negative  (3 2) Source signals  (3 3) Background noise  (3 3 1) Diffw with car  (3 3 2) Diffw pub/restaurant  (3 4) Room effects  (3 4 1) Audiology booth  (3 4 2) Home  (3 4 3) Musical stages  (3 4 4) Religious buildings  (3 4 5) Teaching and rehearsal rooms  (3 5) Second amplification (HA tech)  **(4) HA-technology related difficulties**  (3 1) Diffw ALD use  (3 2) Diffw automatic functions for speech  (3 2 1) Feedback manager  (3 2 2) Microphone directionality  (3 2 3) Noise reduction  (3 3) Diffw dynamics, loudness, compression  (3 4) Diffw freq range or pitch perception  (3 5) Diffw gain  (3 6) Diffw headphones  (3 7) Diffw latency | (3 8) Diffw sonic artefacts  (3 8 1) Audible to others  (3 8 2) Distortion  (3 8 3) Feedback  (3 8 4) Other phantom sounds  (3 9) Diffw sound quality-tone-timbre  (3 9 1) Spectral balance  (3 9 2) Too little bass  (3 9 3) Too much treble  (3 10) General difficulties  **(5) HA-technology strategies**  (5 1) ALD use strategy  (5 2) Compression alterations  (5 3) Disabling automatic functions  (5 3 1) Feedback manager  (5 3 2) Microphone directionality  (5 3 3) Noise or wind reduction  (5 3 4) Speech processing functions  (5 4) Earplugs/hearing protection  (5 5) Gain alterations  (5 6) General HA adjustments  (5 7) HA programs implemented  (5 7 1) Loop program not on HA  (5 7 2) Loop program on HA  (5 7 3) Music program not on HA  (5 7 4) Music program on HA  (5 8) HA use or model selection  (5 9) Headphone use  (5 10) Mould alteration  (5 11) Remove HAs  (5 12) Volume control on HA  **(6) HA users’ reports of others**  (6 1) Aud helpful/interested  (6 2) Aud lacks knowledge  (6 3) Aud managing expectations  (6 4) Aud mixed helpfulness  (6 5) Aud questions usefulness  (6 6) Aud reached technical limit  (6 7) Aud unhelpful/uninterested  (6 8) Manufacturer unhelpful/uninterested | **(7) HA users’ reports of self**  (7 1) Deaf music lovers overlooked  (7 2) Deaf musicians stigma of HL  (7 3) Experience of HAs positive  (7 4) Experience of HAs mixed  (7 5) Experience of HAs negative  (7 6) Experience music positive  (7 7) Experience music mixed  (7 8) Experience music negative  (7 9) Financial affordances/constraints  (7 10) Impact of HL high  (7 11) Impact of HL low  (7 12) Negative mental states  (7 13) Passion for music  (7 14) Perseverance of HA user  (7 15) Rarely/Never listen to music  (7 16) Reduced emotional power of music  (7 17) Reduced pleasure/joy  **(8) Helpfulness of HAs for Music**  (8 1) Cannot hear music without HAs  (8 2) Generally helpful  (8 3) Hear out musical elements  (8 4) Helpful as long as music not too loud  (8 5) Helpful for performance  (8 6) Helpful if combined with T Loop  (8 7) Helpful is music is not complex  (8 8) Helpful with additional adjustments by audiologist  (8 9) Music programme helpful  (8 10) No difficulties  (8 11) Streaming and phone app  **(9) Hearing loss**  (9 1) Acoustic neuroma  (9 2) Asymmetrical HL  (9 3) Deaf during childhood  (9 4) Diffw HL and deteriorating hearing  (9 5) Meniere’s disease  (9 6) Music-induced HL | (9 7) Noise-induced HL  (9 8) Otosclerosis  (9 9) Residual hearing  (9 10) Tinnitus  **(10) Musical experience**  (10 1) Level – amateur  (10 2) Level – professional  (10 3) Level – semi-professional  (10 4) Role – compose, mix, produce  (10 5) Role – listener  (10 6) Role – perform  (10 7) Role – teach  (10 8) Role – undisclosed and other  **(11) Musical instruments**  (11 1) Brass  (11 2) Keyboards – organ  (11 3) Keyboards – piano  (11 4) Keyboards – unspecified  (11 5) Percussion  (11 6) Strings (bowed eg vln, vc)  (11 7) Strings (plucked: guitar, harp)  (11 8) Unspecified/grouped instruments  (11 9) Voice  (11 10) Woodwind  **(12) Music-related difficulties**  (12 1) Diffw aspects  (12 1 1) Frequency domain (spectral)  (12 1 2) Spatial  (12 1 3) Time domain (temporal)  (12 2) Diffw balance-mix-ensemble  (12 3) Diffw clarity  (12 4) Diffw dynamics  (12 4 1) Controlling volume  (12 4 2) Loud  (12 4 3) Range  (12 4 4) Soft  (12 4 5) Volume changes  (12 4 6) Live settings too loud  (12 5) Diffw fatigue-discomfort  (12 6) Diffw hearing lyrics | (12 7) Diffw hearing others’ sounds  (12 8) Diffw hearing own sound  (12 9) Diffw with speech at music event  (12 10) Diffw learning by ear  (12 11) Diffw live – crowds  (12 11) Diffw live – crowds  (12 12) Diffw live greater than recorded  (12 13) Diffw live less or same as recorded  (12 14) Diffw recognising/following music  (12 15) Diffw segregating instruments  (12 16) Sounds wrong-inaccurate-unnatural  (12 17) Uncategorised musical difficulties  (12 18) Uncategorised musical diffw non-HL  **(13) Music-related strategies**  (13 1) Allies  (13 2) Attend events for social aspects  (13 3) Avoid live music  (13 4) Avoid loud music  (13 5) Multi-modal cues  (13 6) Imagining sound, reading music  (13 7) Practice perseverance  (13 8) Select familiar music, remembering  (13 9) Select instrumentation-ensemble  (13 10) Select musical features-repertoire-genres  (13 11) Select rhythm-beat  (13 12) Select venue or room position  (13 12 1) Listen alone  (13 12 2) Listen in the car  (13 12 3) Select position  (13 12 4) Select venue  (13 13) Select vocal/lyrics  (13 14) Tone control in music environments  (13 15) Volume control in music environments  **(14) Strategy did not fix problem**  (no sub-themes) |
| --- | --- | --- | --- | --- |

### SM5a. Age distribution (N=1,507)

### SM5b. Age x Gender distribution (n=1,503, *removing ‘Prefer not to say’ n=4*)

### SM6. Musical training score (Min = 0, Max = 20, n = 1,507)(reflects total score where musical activities maximum score = 7, music educational qualifications maximum score = 6, and instrumental performance history maximum score = 7)

### SM7a. Musical engagement scores (N=1,507) showing rated agreement with seven engagement items.

### SM7b. Distribution of scores on a 6-item engagement scale (NB: using music streaming removed) where -12 indicates strong disagreement with all items, 0 is neutral, and +12 indicates strong agreement with all items (N=1,507).

### SM7c. Overall musical engagement score for each hearing loss level (n = 1,425, max score = 30, **p < 0.001).


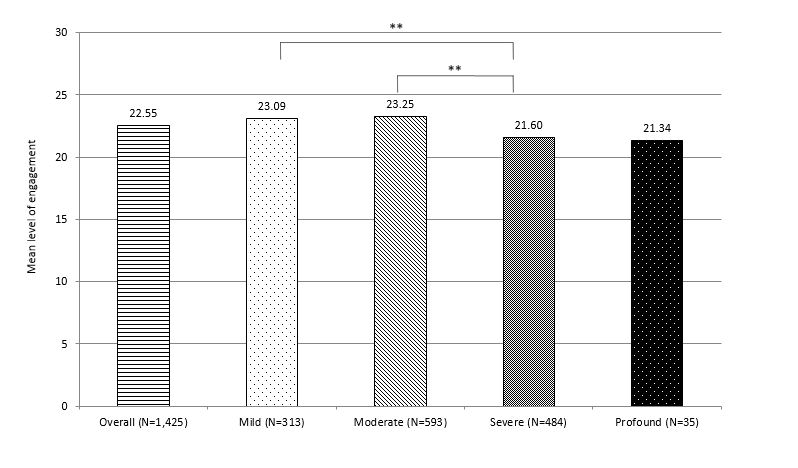


### SM7d. Likelihood of avoiding music listening for each hearing loss level (N=1,425)

### SM8a Preference ratings for 18 styles, where ‘0’ is neither disagree nor agree liking (NB: N varies across styles as participants were given the option not to rate styles they did not know or listen to, minimum score = -0.85, maximum score = 0.98).

### SM8b. Preference ratings for each HL level for four of the 18 styles, selected because those with more severe HL reported lower preferences (**p* < 0.05, ** *p*< 0.01).

### SM8c. Importance of musical features for music appreciation (n = 1,434) where ‘0’ is neither disagree nor agree liking (NB: N varies across styles as participants were given the option not to rate styles they did not know or listen to, minimum score = -0.16, maximum score = 1.09).

### SM9. Dome type statistics

**What type of dome or mould does your hearing aid have? (n = 1,409)**

### SM10. Music program statistics. A) Do you have a music program? B) How often do you use your music program?

| 1. Do you have a music program? (n = 1,409) | 1. How often do you use your music program? (n = 480) |
| --- | --- |

### SM11. Listening to recorded music descriptives overall and for each hearing loss level.

| Wear HAs for recorded music |  | Overall (n=1406) | Mild (n=307) | Moderate (n=585) | Severe (n=480) | Profound (n=34) |
| --- | --- | --- | --- | --- | --- | --- |
|  | Yes or Sometimes | 1175 (83.6%) | 266 (86.6%) | 505 (86.3%) | 376 (78.3%) | 28 (82.4%) |
|  | No or N/A | 231 (16.4%) | 41 (13.4%) | 80 (13.7%) | 104 (21.7%) | 6 (17.7%) |
| Hours listening to recorded music |  | Overall (n=1119) | Mild (n=248) | Moderate (n=483) | Severe (n=362) | Profound (n=26) |
|  | None or 1-2 hours | 314 (30.5%) | 63 (25.4%) | 135 (28.0%) | 133 (36.7%) | 10 (38.5%) |
|  | 3-4 hours | 279 (24.9%) | 71 (28.6%) | 109 (22.6%) | 94 (26.0%) | 5 (19.2%) |
|  | 5-10 hours | 256 (22.9%) | 49 (19.8%) | 134 (27.7%) | 71 (19.6%) | 2 (7.7%) |
|  | More than 10 hours | 243 (21.7%) | 65 (26.2%) | 105 (21.7%) | 64 (17.7%) | 9 (34.6) |
| Listen with hearing aids... |  |  |  |  |  |  |
| *... through loudspeakers* |  | Overall (n=1055) | Mild (n=241) | Moderate (n=461) | Severe (n=328) | Profound (n=25) |
|  | At least sometimes | 1022 (96.9%) | 225 (93.4%) | 451 (97.8%) | 321 (97.9%) | 25 (100%) |
|  | Without HAs | 33 (3.1%) | 16 (6.6%) | 10 (2.2%) | 7 (2.1%) | 0 (0%) |
| *... through headphones* |  | Overall (n=699) | Mild (n=164) | Moderate (n=313) | Severe (n=208) | Profound (n=14) |
|  | At least sometimes | 386 (55.2%) | 78 (47.6%) | 158 (50.5%) | 138 (66.4%) | 12 (85.7%) |
|  | Without HAs | 313 (44.8%) | 86 (52.4%) | 155 (49.5%) | 70 (33.7%) | 2 (14.3%) |
| *... through car stereo* |  | Overall (n=934) | Mild (n=215) | Moderate (n=407) | Severe (n=293) | Profound (n=19) |
|  | At least sometimes | 883 (94.5%) | 200 (93.0%) | 379 (93.1%) | 285 (97.3%) | 19 (100%) |
|  | Without HAs | 51 (5.5%) | 15 (7.0%) | 28 (6.9%) | 8 (2.7%) | 0 (0%) |

### SM12. Listening to live music descriptives overall and for each hearing loss level.

| Wear hearing aids at live events |  | Overall (n=1345) | Mild (n=287) | Moderate (n=560) | Severe (n=466) | Profound (n=32) |
| --- | --- | --- | --- | --- | --- | --- |
|  | Yes or Sometimes | 968 (72.0%) | 195 (67.9%) | 415 (74.1%) | 338 (72.5%) | 20 (62.5%) |
|  | No or N/A | 377 (28.0%) | 92 (32.1%) | 145 (25.9%) | 128 (27.5%) | 12 (37.5%) |
| Number live events in last 12 months |  | Overall (n=931) | Mild (n=188) | Moderate (n=398) | Severe (n=326) | Profound (n=19) |
|  | 0, 1 or 2 | 319 (34.3%) | 55 (29.3%) | 132 (33.2%) | 122 (37.4%) | 10 (52.6%) |
|  | 3 to 6 | 304 (32.7%) | 59 (31.4%) | 127 (31.9%) | 113 (34.7%) | 5 (26.3%) |
|  | 7 or more | 308 (33.1%) | 74 (39.4%) | 139 (34.9) | 91 (27.9%) | 4 (21.1%) |
| Type of live events |  | Overall (n=931) | Mild (n=188) | Moderate (n=398) | Severe (n=326) | Profound (n=19) |
|  | 100% acoustic | 231 (24.8%) | 44 (23.4%) | 106 (26.6%) | 77 (23.6%) | 4 (21.1%) |
|  | 100% amplified | 260 (27.9%) | 53 (28.2%) | 100 (25.1%) | 98 (30.1%) | 9 (47.4%) |
|  | Both | 440 (47.3%) | 91 (48.4%) | 192 (48.2%) | 151 (46.3%) | 6 (31.6%) |
| Listen with hearing aids... |  | Overall (n=837) | Mild (n=178) | Moderate (n=357) | Severe (n=287) | Profound (n=15) |
| to live acoustic music | At least sometimes | 823 (98.3%) | 174 (97.8%) | 350 (98.0%) | 284 (99.0%) | 15 (100%) |
|  | Without HAs | 14 (1.7%) | 4 (2.3%) | 7 (2.0%) | 3 (1.1%) | 0 (0%) |
|  |  | Overall (n=777) | Mild (n=161) | Moderate (n=326) | Severe (n=275) | Profound (n=15) |
| .. to live (amplified) music | At least sometimes | 726 (93.4%) | 151 (93.8%) | 294 (90.2%) | 266 (96.7%) | 15 (100%) |
|  | Without HAs | 51 (6.6%) | 10 (N=6.2%) | 32 (9.8%) | 9 (3.3%) | 0 (0%) |
|  |  | Overall (n=628) | Mild (n=122) | Moderate (n=284) | Severe (n=209) | Profound (n=13) |
| when playing an instrument/singing | At least sometimes | 585 (93.2%) | 113 (92.6%) | 254 (89.4%) | 205 (98.1%) | 13 (100%) |
|  | Without HAs | 43 (6.9%) | 9 (7.4%) | 30 (10.6%) | 4 (1.9%) | 0 (0%) |

### SM13. Descriptive data and paired comparisons for Helpfulness, Difficulties and Strategies in recorded and live music contexts.

|  |  |  | Recorded  (n=1,119)  Count (%)  Mean (SD) | Live  (n=931)  Count (%)  Mean (SD) | **Significant difference?** (NB: Those who answered both. Excludes ‘Don’t know’ and ‘My HA does this automatically’ responses) |
| --- | --- | --- | --- | --- | --- |
| **Helpfulness of HAs** | Hearing the melody | Not at all helpful  Somewhat helpful  Medium  Fairly helpful  Very helpful  Don't know | 78 (7.0)  176 (15.7)  174 (15.6)  266 (23.8)  362 (32.3)  63 (5.6) | 70 (7.5)  179 (19.2)  165 (17.8)  231 (24.8)  243 (26.1)  43 (4.6) |  |
|  |  |  | n=790  3.76 (1.25)  Median 4  Mode 5 | n=790  3.52 (1.25)  Median 4  Mode 5 | *W* = 24588.00  *Z* = -5.848  ***p* < 0.001****  *r = 0.21* |
|  | Hearing the bassline | Not at all helpful  Somewhat helpful  Medium  Fairly helpful  Very helpful  Don't know | 150 (13.4)  196 (17.5)  184 (16.4)  235 (21.0)  266 (23.8)  88 (7.9) | 124 (13.3)  163 (17.5)  186 (20.0)  203 (21.8)  182 (19.6)  73 (7.8) |  |
|  |  |  | n=758  3.39 (1.38)  Median 4  Mode 5 | n=758  3.25(1.32)  Median 3  Mode 4 | *W* = 26300.00  *Z* = -3.665  ***p*< 0.001****  *r = 0.13* |
|  | Hearing the singer | Not at all helpful  Somewhat helpful  Medium  Fairly helpful  Very helpful  Don't know | 70 (6.3)  176 (15.7)  202 (18.1)  262 (23.4)  355 (31.7)  54 (4.8) | 78 (8.4)  181 (19.4)  158 (16.9)  236 (25.4)  238 (25.6)  40 (4.3) |  |
|  |  |  | n=800  3.72 (1.23)  Median 4  Mode 5 | n=800  3.49 (1.28)  Median 4  Mode 5 | *W* = 26379.00  *Z* = -5.617  ***P* < 0.001****  *r=0.20* |
|  | Hearing lyrics | Not at all helpful  Somewhat helpful  Medium  Fairly helpful  Very helpful  Don't know | 178 (15.9)  202 (18.1)  185 (16.5)  231 (20.6)  272 (24.3)  51 (4.6) | 154 (16.5)  190 (20.4)  178 (19.1)  188 (20.2)  182 (19.6)  39 (4.2) |  |
|  |  |  | n=801  3.28 (1.4)  Median 3  Mode 5 | n=801  3.11(1.4)  Median 3  Mode 4 | *W* = 30139.00  *Z* = -4.327  ***p* < 0.001****  *r = 0.15* |
|  | Picking out instruments | Not at all helpful  Somewhat helpful  Medium  Fairly helpful  Very helpful  Don't know | 187 (16.7)  208 (18.6)  162 (14.5)  239 (21.4)  245 (21.9)  78 (6.9) | 143 (15.4)  190 (20.4)  166 (17.8)  184 (19.8)  186 (19.9)  62 (6.7) |  |
|  |  |  | n=772  3.26 (1.4)  Median 3  Mode 5 | n=772  3.16 (1.4)  Median 3  Mode 4 | *W* = 31138.00  *Z* = -2.477  ***p* = 0.013***  *r = 0.09* |
|  | Overall helpfulness  (Rating out of 10) |  | n=851  6.81 (2.4)  Median 7  Mode 8 | n=851  6.45 (2.4)  Median 7  Mode 8 | *t*(850) = 6.492  *p* < 0.001**  *Cohen’s d 0.22* |
|  |  |  |  |  |  |
|  |  |  | Recorded  (n=1,119)  Count (%) | Live  (n=931)  Count (%) |  |
| **Difficulties** | Distortion | Never  Occasionally  Sometimes  Often  All the time  Don't know | 207 (18.5)  234 (20.9)  294 (26.3)  213 (19.0)  101 (9.0)  70 (6.3) | 163 (17.5)  213 (22.9)  261 (28.0)  155 (16.6)  92 (9.9)  47 (5.1) |  |
|  |  | Never  At least occasionally | n=785  158 (20.1)  627 (79.9) | n=785  147 (18.7)  638 (81.3) | χ^2^ = 0.935  *p =* 0.334  *W = 0.03* |
|  | Too much bass | Never  Occasionally  Sometimes  Often  All the time  Don't know | 413 (36.9)  217 (19.4)  212 (18.9)  100 (8.9)  32 (2.9)  145 (13.0) | 288 (30.9)  191 (20.5)  214 (23.0)  96 (10.3)  40 (4.3)  102 (11.0) |  |
|  |  | Never  At least occasionally | n=729  310 (42.5)  419 (57.5) | n=729  252 (34.6)  477 (65.4) | χ^2^ = 23.543  ***p* < 0.001****  *W = 0.18* |
|  | Too much treble | Never  Occasionally  Sometimes  Often  All the time  Don't know | 282 (25.2)  235 (21.0)  248 (22.2)  145 (12.9)  58 (5.2)  151 (13.5) | 229 (24.6)  192 (20.6)  236 (25.4)  113 (12.1)  46 (4.9)  115 (12.4) |  |
|  |  | Never  At least occasionally | n=723  218 (30.2)  505 (69.8) | n=723  201 (27.8)  522 (72.2) | χ^2^ =2.306  *p = 0*.128  *W = 0.06* |
|  | Feedback | Never  Occasionally  Sometimes  Often  All the time  Don't know | 478 (42.7)  231 (20.6)  232 (20.7)  105 (9.4)  25 (2.3)  48 (4.3) | 402 (43.2)  191 (20.5)  156 (16.8)  101 (10.8)  28 (3.0)  53 (5.7) |  |
|  |  | Never  At least occasionally | n=799  368 (46.1)  431 ((53.9) | n=799  371 (46.4)  428 (53.6) | χ^2^ = 0.033  *p* = 0.857  *W = 0.01* |
|  | Sudden changes in loudness | Never  Occasionally  Sometimes  Often  All the time  Don't know | 485 (43.4)  214 (19.1)  204 (18.2)  121 (10.8)  38 (3.4)  57 (5.1) | 364 (39.1)  188 (20.2)  195 (20.9)  95 (10.2)  34 (3.7)  55 (5.9) |  |
|  |  | Never  At least occasionally | n=791  382 (48.3)  409 (51.7) | n=791  327 (41.3)  464 (58.7) | χ^2^ = 20.392  ***p* < 0.001****  *W = 0.16* |
|  | Discomfort from loud sounds | Never  Occasionally  Sometimes  Often  All the time  Don't know | 363 (32.4)  252 (22.5)  241 (21.6)  153 (13.7)  67 (6.0)  43 (3.8) | 228 (24.5)  213 (22.9)  215 (23.1)  170 (18.2)  69 (7.4)  36 (3.9) |  |
|  |  | Never  At least occasionally | n=808  285 (35.3)  523 (64.7) | n=808  210 (26.0)  598 (74.0) | χ^2^ = 32.023  ***p* < 0.001****  *W = 0.20* |
|  |  |  |  |  |  |
|  |  |  | Recorded  (n=1,119)  Count (%) | Live  (n=931)  Count (%) |  |
| **Strategies** | Adjust volume | Never  Occasionally  Sometimes  Often  All the time  HA automatically | 364 (32.5)  215 (19.2)  228 (20.4)  174 (15.6)  65 (5.8)  73 (6.5) | 246 (26.4)  185 (19.9)  180 (19.3)  170 (18.3)  76 (8.2)  74 (7.9) |  |
|  |  | Never  At least occasionally | n=771  250 (32.4)  521 (67.6) | n=771  220 (28.5)  551 (71.5) | χ^2^ = 7.250  ***p* = 0.007***  *W = 0.10* |
|  | Change programme | Never  Occasionally  Sometimes  Often  All the time  HA automatically | 591 (52.8)  142 (12.7)  158 (14.1)  112 (10.0)  63 (5.63)  53 (4.73) | 429 (46.1)  99 (10.6)  144 (15.5)  128 (13.7)  64 (6.9)  67 (7.2) |  |
|  |  | Never  At least occasionally | n=785  409 (52.1)  376 (47.9) | n=785  396 (50.4)  389 (49.6) | χ^2^ = 1.455  *p* = 0.228  *W = 0.04* |
|  | Move away from sound source | Never  Occasionally  Sometimes  Often  All the time | 438 (39.1)  287 (25.7)  256 (22.9)  109 (9.7)  29 (2.6) | 333 (35.8)  251 (26.9)  191 (20.5)  106 (11.4)  50 (5.4) |  |
|  |  | Never  At least occasionally | n=755  332 (44.0)  423 (56.0) | n=755  303 (40.1)  452 (59.9) | χ^2^ = 4.193  ***p* = 0.040***  *W = 0.075* |
|  |  |  |  |  |  |

### SM14 Helpfulness of hearing aids, difficulties experienced and strategies in recording and live music settings – inferential tests summary

|  | | **Sample** | **Average** | **Recorded** | **Live** | **Test** |
| --- | --- | --- | --- | --- | --- | --- |
| **Helpfulness** | Overall Helpfulness (1-10) | n=851 | Mean (SD) | 6.81 (2.35) | 6.45 (2.44) | *t*(850)= 6.492, ***p*<.001****, Cohen’s d 0.22 |
|  | Hearing the melody | n=790 | Mean (SD) | 3.76 (1.25) | 3.52 (1.25) | W = 24588.00, Z= -5.848, ***p*<.001****, r=0.21 |
|  | Hearing the bassline | n=758 | Mean (SD) | 3.39 (1.38) | 3.25 (1.32) | W= 26300.00, Z= -3.665, ***p*<.001****, r=0.13 |
|  | Hearing the singer | n=800 | Mean (SD) | 3.72 (1.23) | 3.49 (1.28) | W= 26379.00, Z= -5.617, ***p*<.001****, r=0.20 |
|  | Hearing the lyrics | n=801 | Mean (SD) | 3.28 (1.41) | 3.11 (1.37) | W= 30139.00, Z= -4.327, ***p*<.001****, r=0.15 |
|  | Picking out instruments | n=772 | Mean (SD) | 3.26 (1.42) | 3.16 (1.36) | W= 31138.00, Z= -2.477, ***p*=.013***, r=0.09 |
|  |  | **Sample** | **Count (%)** | **Recorded** | **Live** | **Test** |
| **Difficulties** | Distortion | n=785 | Never | 158 (20.1) | 147 (18.7) | χ^2^ = 0.935, *p* = 0.334, W = 0.03 |
|  |  |  | At least occasionally | 627 (79.9) | 638 (81.3) |  |
|  | Too much bass | n=729 | Never | 310 (42.5) | 252 (34.6) | χ^2^ = 23.543, ***p* < 0.001****, W = 0.18 |
|  |  |  | At least occasionally | 419 (57.5%) | 477 (65.4) |  |
|  | Too much treble | n=723 | Never | 218 (30.2) | 201 (27.8) | χ^2^ = 2.306, *p* = 0.128, W = 0.06 |
|  |  |  | At least occasionally | 505 (69.8) | 522 (72.2) |  |
|  | Feedback | n=799 | Never | 368 (46.1) | 371 (46.4) | χ^2^ = 0.033, *p* = 0.857, W = 0.01 |
|  |  |  | At least occasionally | 431 ((53.9) | 428 (53.6) |  |
|  | Sudden changes in loudness | n=791 | Never | 382 (48.3) | 327 (41.3) | χ^2^ = 20.392, ***p* < 0.001****, W = 0.16 |
|  |  |  | At least occasionally | 409 (51.7) | 464 (58.7) |  |
|  | Discomfort from loud sounds | n=808 | Never | 285 (35.3) | 210 (26.0) | χ^2^ = 32.023, ***p* < 0.001****, W = 0.20 |
|  |  |  | At least occasionally | 523 (64.7) | 598 (74.0) |  |
| **Strategies** | Adjust volume | n=771 | Never | 250 (32.4) | 220 (28.5) | χ^2^ = 7.250, ***p* = 0.007***, *W* = 0.10 |
|  |  |  | At least occasionally | 521 (67.6) | 551 (71.5) |  |
|  | Change program | n=785 | Never | 409 (52.1) | 396 (50.4) | χ^2^ = 1.455, *p* < 0.228, *W* = 0.04 |
|  |  |  | At least occasionally | 376 (47.9) | 389 (49.6) |  |
|  | Move away from sound source | n=755 | Never | 332 (44.0) | 303 (40.1) | χ^2^ = 4.193, ***p* = 0.040***, *W* = 0.075 |
|  |  |  |  |  |  |  |
